# A cotton miRNA is involved in regulation of plant response to salt stress

**DOI:** 10.1038/srep19736

**Published:** 2016-01-27

**Authors:** Shuai Gao, Lu Yang, Hou Qing Zeng, Zhao Sheng Zhou, Zhi Min Yang, Hua Li, Di Sun, Fuliang Xie, Baohong Zhang

**Affiliations:** 1 Department of Biochemistry and Molecular Biology, College of Life Science, Nanjing Agricultural University, Nanjing 210095, China; 2 College of Resources and Environmental Science, Nanjing Agricultural University, Nanjing, China; 3 Department of Plant Science, College of Life Science, Henan Agricultural University, Henan 450002, China; 4 Department of Biology, East Carolina University, Greenville, NC 27858, USA; 5 College of Life and Environmental Sciences, Hangzhou Normal University, Hangzhou 310036, China; 6 Department of Biochemistry and Biophysics, College of Agriculture and Life Sciences, Texas A&M University, TA 77843, USA

## Abstract

The present study functionally identified a new microRNA (microRNA ovual line 5, miRNVL5) with its target gene *GhCHR* from cotton (*Gossypium hirsutum*). The sequence of miRNVL5 precursor is 104 nt long, with a well developed secondary structure. GhCHR contains two DC1 and three PHD Cys/His-rich domains, suggesting that *GhCHR* encodes a zinc-finger domain-containing transcription factor. miRNVL5 and *GhCHR* express at various developmental stages of cotton. Under salt stress (50–400 mM NaCl), miRNVL5 expression was repressed, with concomitant high expression of *GhCHR* in cotton seedlings. Ectopic expression of *GhCHR* in *Arabidopsis* conferred salt stress tolerance by reducing Na^+^ accumulation in plants and improving primary root growth and biomass. Interestingly, *Arabidopsis* constitutively expressing miRNVL5 showed hypersensitivity to salt stress. A *GhCHR* orthorlous gene At2g44380 from *Arabidopsis* that can be cleaved by miRNVL5 was identified by degradome sequencing, but no confidential miRNVL5 homologs in *Arabidopsis* have been identified. Microarray analysis of miRNVL5 transgenic *Arabidopsis* showed six downstream genes (*CBF1*, *CBF2*, *CBF3*, *ERF4*, *AT3G22920*, and *AT3G49200*), which were induced by salt stress in wild-type but repressed in miRNVL5-expressing *Arabidopsis*. These results indicate that miRNVL5 is involved in regulation of plant response to salt stress.

Soil salinity is one of the major environmental and agronomical problems which seriously cause cellular dehydration and ionic toxicity, thus limiting the productivity of crops^1^. To survive the detrimental effect of salt stress, plants have developed various elaborate mechanisms to exclude excess salt from their cells or to tolerate salt within the cells^2^. Plant resistance to salt stress is controlled by multiple genes and is regulated at multiple levels. At the molecular level, various stress-responsive genes are involved^3^,^4^.

Recently, a large group of small RNAs, termed microRNAs (miRNAs), are reported as regulators in plant adaptation to abiotic stresses^5^,^6^,^7^,^8^. For examples, transgenic creeping bentgrass (*Agrostis stolonifera*) overexpressing rice miR319a exhibited enhanced salt and drought tolerance^9^; overexpressing miR396c and miR394 plants was hypersensitive to saline stress^10^,^11^. In cotton (*Gossypium hirsutum*), a group of miRNAs and their targets have been identified, and some of them respond to salt and drought stresses^12^,^13^,^14^,^15^,^16^,^17^. But to date, only a few of cotton miRNAs have been functionally characterized^18^.

Abiotic stress-responsive genes are largely regulated by complex networks of transcription factors (TFs)^19^,^20^,^21^. One of them is the zinc finger protein (ZFP) family, which bind to zinc ions through their Cystenine (Cys) and Histidine (His) and are involved in protein-DNA or protein-protein interactions in plants^22^. ZFP family proteins can be categorized based on the combinations of Cys and His for coordination divalent zinc ions, such as C2H2, C3HC4, C4, CCCH, C4HC3 or C2HC5^23^. Each ZFP protein may contain one or more finger zinc motifs, which is incorporated into diverse functional proteins. The PHD (plant homeodomain) finger protein genes encode another type of zinc finger proteins whose motifs comprise ∼60 amino acids and typically show a C4HC3 signature (four Cys, one His, and three Cys); with the PHD-finger domain, PHD proteins are able to bind to a common nuclear ligand chromatin and play an important role in regulating chromatin remodeling^24^. In *Arabidopsis*, PHD domain-containing protein genes regulate diverse processes of development^25^,^26^. Importantly, some of the genes were reported to regulate plant response to salt stress^27^,^28^. In this study, we functionally identified a miRNA (designed as miRNVL5), along with its target gene from cotton (*G. hirsutum*). miRNVL5 and GhCHR were originally isolated from cotton ovules from our previous study^13^. RACE-PCR analysis showed that miRNVL5 guides the cleavage of the target harboring two DC1 and three PHD domains (Due to its containing a Cys/His-rich DC1/PHD domains, here referred as *GhCHR*). Our data showed that salt stress repressed the miRNVL5 expression, whereas induced the *GhCHR* expression in cotton. Transgenic *Arabidopsis* constitutively expressing miRNVL5 showed sensitivity to salt stress, while expression of *GhCHR* improved salt tolerance in *Arabidopsis*. These results indicate that miRNVL5 and *GhCHR* play an important role in plant response to salt stress.

## Results

### miRNVL5 was a cotton miRNA targeting a zinc finger protein family gene *GhCHR*

**Figure 1:**
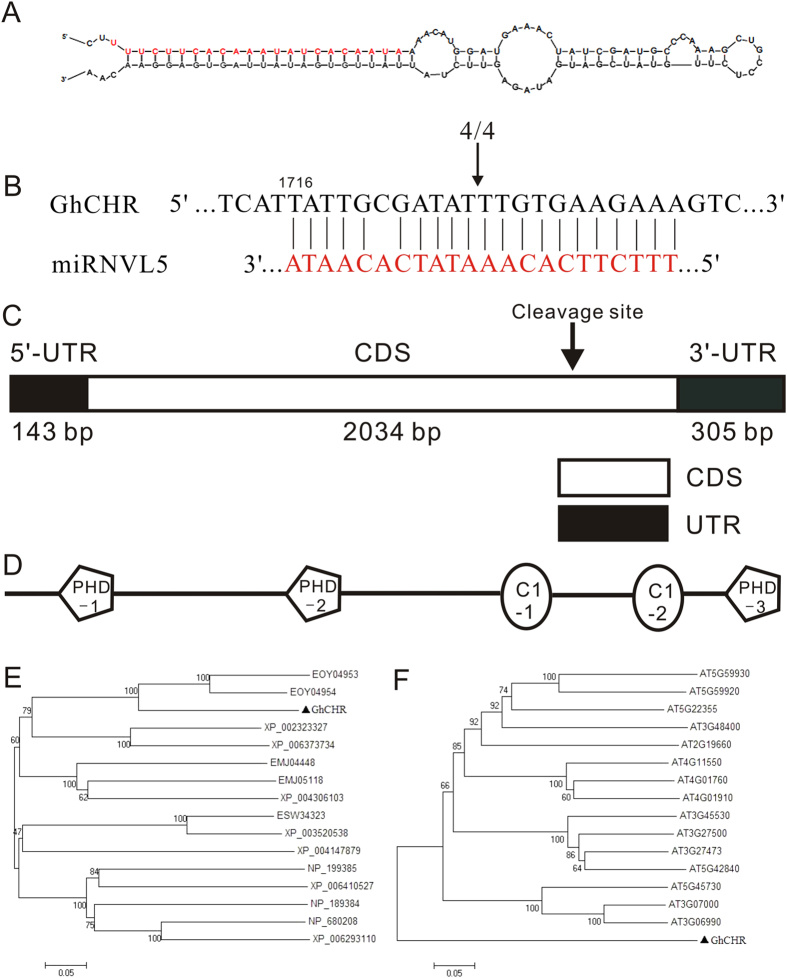
Basic information on miRNVL5 and *GhCHR* from cotton (*Gossypium hirsutum*). (**A**) Mature (in red) and precursor sequences and the stem-loop structure of miRNVL5. (**B**) The *GhCHR* sequence with nucleotides was cleaved by miRNVL5 using 5′-RACE. (**C**) cDNA structure of *GhCHR*. (**D**) Predicted *GhCHR* protein structure with PHD and C1 domains. (**E**) Dendrogram showing the relationship among genes encoding PHD-type proteins in cotton and other plant species. (**F**) Dendrogram showing PHD-type proteins between cotton and Arabidopsis only.

The sequence of miRNVL5 precursor is 104 nt in length, with a 22-nt mature sequence and well developed secondary structure (Fig. 1A; Data S1). Homologous search revealed that no sequences perfectly or near-perfectly match the mature miRNVL5 in *Arabidopsis* and other plant species. *GhCHR* was predicted to be the only target of miRNVL5 in *G. hirsutum*. 5′ complementary DNA ends (5′-RACE)-PCR analysis was performed to validate the cleavage site (Fig. 1B). The PCR products were cloned and sequenced. The four clones showed the same site of cleavage following the eleventh nucleotide from the 5′ end of miRNVL5, indicating that the *GhCHR* transcript is the real target of miRNVL5.

The cDNA of *GhCHR* was 2482 bp in length including a 2034-bp open reading frame, a 143-bp 5′ untranslated region (UTR), and a 305-bp 3′ UTR (Fig. 1C, Data S2A). The deduced *GhCHR* polypeptide consists of 677 residues of amino acids, with a predicted molecular weight of 23.7 kD and a pI of 6.54. A Blastp search showed that GhCHR contains three PHD (Plant Homeo Domain with Cys_4_-His-Cys_3_ motif) and two DC1 (Cys_5_-His) motifs capable of binding zinc (Fig. 1D, Data S2B), indicating that *GhCHR* transcripts encode a PHD-domain containing protein^25^. A phylogenetic analysis showed that *GhCHR* protein shared only 59% identity with a cysteine/histidine-C1 domain family protein from Cacao (*Theobroma cacao*; EOY04953; Fig. 1E,F). Multiple sequence alignments using BLASTx showed that the *GhCHR* protein also shared very low similarity with the proteins from other plant species except for PDH and C1 domains (Data S2C), suggesting that GhCHR is not a highly conserved CHC domain (Cys4-His-Cys3)-containing zinc finger protein.

### Expression patterns of miRNVL5 and *GhCHR* under salinity stress

**Figure 2:**
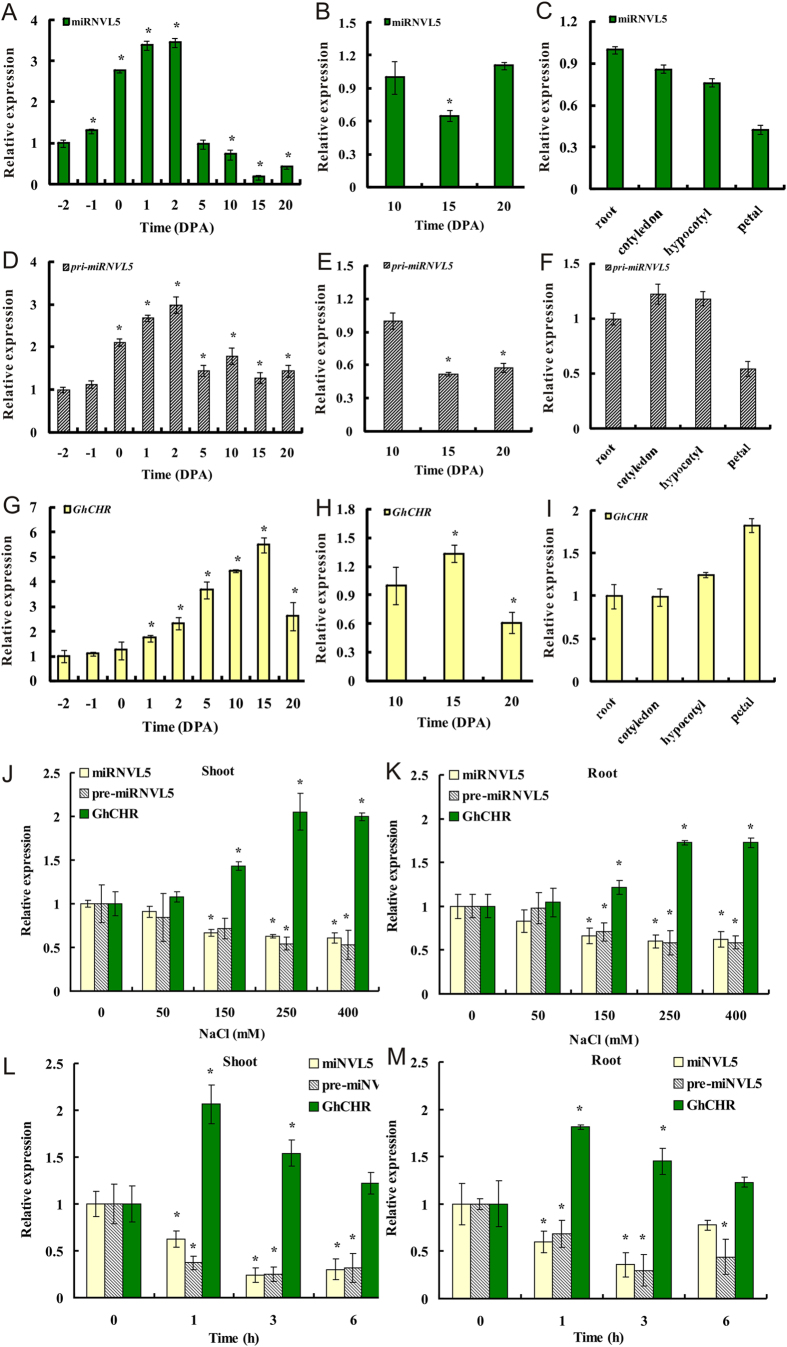
Expression of miRNVL5, *pri-miRNVL5* and *GhCHR* in different tissues of cotton with or without salt treatment. (**A,D,G**) Genes from ovules of −2–20 days post anthesis (DPA). (**B,E,H**) Genes from developing fibers of 10, 15 and 20 DPA. (**C,F,I**) genes from other tissues. (**J**–**M**) Two week-old cotton seedlings were treated with NaCl (0–400 mM) **(J,K**) for 1 h, or with 250 mM NaCl for 0–6 h (**L,M**). After that, total RNA was extracted and analyzed by real-time PCR. Vertical bars represent SD of the mean with three replicates. Asterisks indicate that mean values are significantly different between the treatment and control (*p* < 0.05).

Quantitative RT-PCR (qRT-PCR) was used to analyze expression patterns of miRNVL5 and pri-miRNVL5 as well as *GhCHR* at different developmental stages of cotton. Both miRNVL5/pri-miRNVL5 and *GhCHR* were ubiquitously expressed at various developmental stages, but the transcript levels changed considerably (Fig. 2A–I). In developing ovules, miRNVL5 and pri-miRNVL5 were highly expressed at 0-2 DPA. After that, expression of miRNVL5 was decreased. In contrast, expression of *GhCHR* was always repressed in correlation to miRNVL5. The inverse expression pattern of miRNVL5 and *GhCHR* suggests that *GhCHR* transcript was negatively regulated by miRNVL5.

Because recent reports indicate that PHD domain-containing protein genes are involved in plant response to abiotic stress^27^,^28^, this urged us to test the transcription of *GhCHR* under environmental stress. In shoots of cotton seedlings, treatment with 150–400 mM NaCl induced the expression of *GhCHR* but repressed the miRNVL5 expression (Fig. 2J). A similar result was found in roots (Fig. 2K). The time-course study with 250 mM NaCl also revealed that expression of miRNVL5 was decreased, whereas the expression of *GhCHR* was increased after 1 h treatment of NaCl (Fig. 2L,M). Compared to the control, treatment with 250 mM NaCl for 1, 3 and 6 h led to the decrease in transcripts of miRNVL5 by 37.3%, 75.6% and 70% in shoots and 39.3%, 64.3% and 22.4% in roots, respectively. These results confirmed the inverse expression pattern for miRNVL5/pri-miRNVL5 and *GhCHR*. The transcriptional responses of miRNVL5 and *GhCHR* were also tested with drought, cold and ABA, but no significant change in the transcriptional response was observed (Data S3).

### Transgenic *Arabidopsis* response to salt stress was regulated by constitutive expression of miRNVL5 and *GhCHR*

In order to identify a role of *GhCHR* in plant response to salt stress, full-length cDNA of *GhCHR* was transformed into *Arabidopsis* wild-type (Col-0 ecotype) with cauliflower mosaic virus 35S promoter. As *GhCHR* mRNA generated in *Arabidopsis* was possibly cleaved by a putative miRNA, we simultaneously constructed an miRNVL5 cleavage-resistant version (*35S::mGhCHR*) by introducing seven silent mutations in the miRNVL5 binding site but without changing the protein sequences (Data S4). Five independent transgenic lines were obtained, and two of them were used for study. The transgenic lines displayed no abnormal phenotypes under normal condition. qRT-PCR analysis showed that the levels of *GhCHR* mRNA in *35S::mGhCHR* and *35S::GhCHR* transgenic lines were 72–89 and 109–132 folds higher than the wide-type, respectively (Data S4A–C). We also generated transgenic *Arabidopsis* plants expressing miRNVL5, because blast search on *Arabidopsis* genome revealed five potential orthologs of *GhCHR* which are possibly targeted by miRNVL5 (data to be presented below). Thus, the pri-miRNVL5 sequence was fused to the CaMV 35S promoter and transformed into the *Arabidopsis* Col-0. Expression analysis showed that the selected transgenic lines of *35S::miRNVL5* had high expression of miRNVL5 (Data S4D).

**Figure 3:**
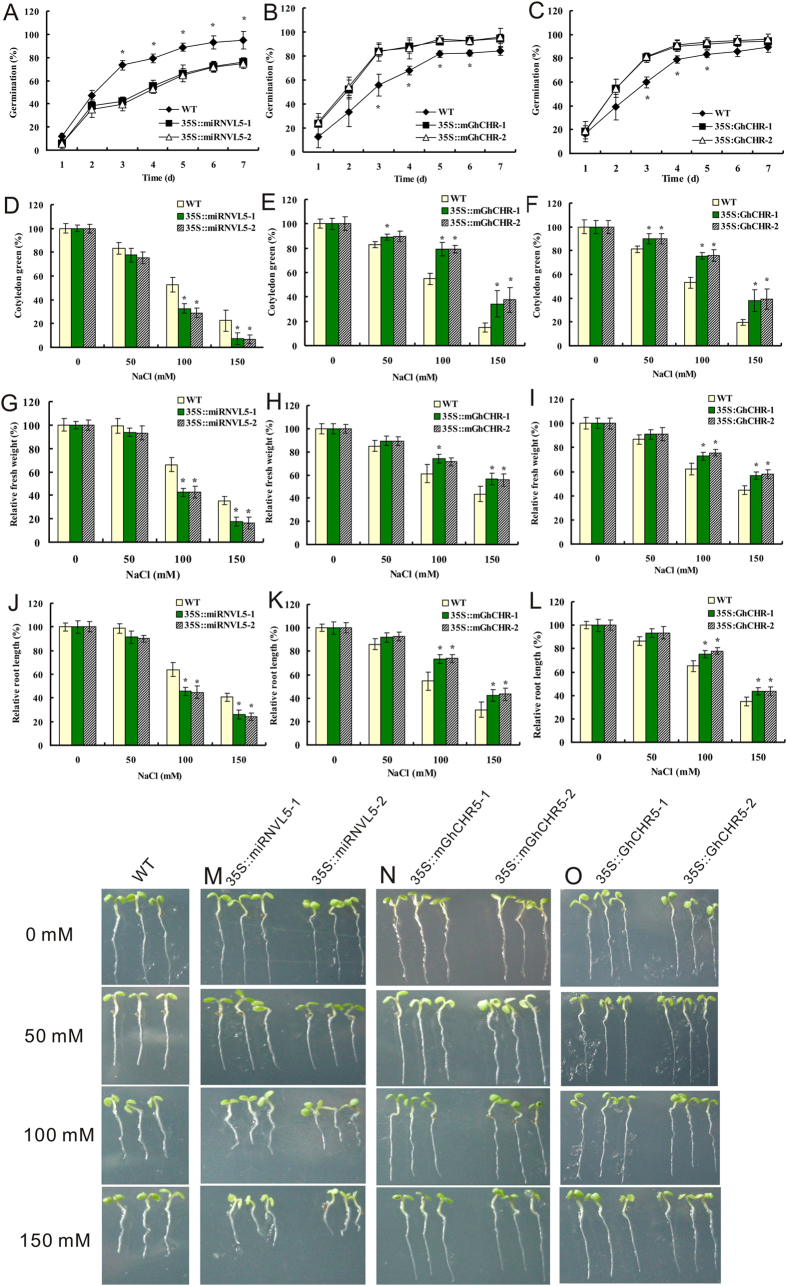
Seed germination and seedling growth of transgenic Arabidopsis over-expressing miRNVL5 (*35::miRNVL5*) (**A,D,G,J,M**), miRNVL5-resistant version (*35S::mGhCHR*)(**B,E,H,K,N**) and *GhCHR* (*35S::GhCHR*)(**C,F,I,L,O**) under the salt stress. (**A–C**) Seeds were germinated on MS medium containing 100 mM NaCl. Germination rates were measured during one week. (**D–F**) Cotyledon greening and expansion of 7 day-old seedlings with 50–150 mM NaCl. (**G–I**) Fresh weight of 7 day-old seedlings exposed to 50–150 mM NaCl. (**J**–**K**) The elongation of primary root of 7 day-old seedlings exposed to 50–150 mM NaCl. M-O: phenotype of primary root growth of 7 day-old seedlings exposed to 50–150 mM NaCl. Vertical bars represent SD of the mean with three replicates. Asterisks indicate that mean values are significantly different between the transgenic plants and wild-type (WT) (*p* < 0.05).

We then investigated the transgenic plants in regulating plant response to salt stress. *Arabidopsis* seeds were placed on the solid MS medium supplemented with 0–150 mM NaCl. The seed germination and post-germination growth responded differently to NaCl after a 1–7 d period of treatment. Following a 3-day treatment with 100 mM NaCl, the seed germination rate of *35S::miRNVL5* lines were only 53.4–57.5% of the wild-type (Fig. 3A), whereas seed germination rate of *35S::mGhCHR* and *35S::GhCHR* plants was 51.9–52.7% and 37.3–39.1% higher than the wild-type, respectively (Fig. 3B,C). Exposure to 100–150 mM NaCl also inhibit cotyledon greening in *35S::miRNVL5* seedlings (Fig. 3D). Under the same condition, the percentage of cotyledon greening in *35S::mGhCHR* and *35S::GhCHR* seedlings was higher than that of the wild-type (Fig. 3E,F). The biomass (fresh weight) of *35S::miRNVL5* and *35S::mGhCHR* or *35S::GhCHR* lines was also examined. Compared to wild-type, lower fresh weight of *35S::miRNVL5* seedlings was observed, whereas higher biomass of *35S::mGhCHR* and *35S::GhCHR* seedlings was determined (Fig. 3G–I). The root elongation of *35S::miRNVL5* lines was significantly inhibited by 100–150 mM NaCl; in contrast, the *35S::mGhCHR* and *35S::GhCHR* seedlings had higher tolerance to 100–150 mM NaCl stress in root growth (Fig. 3J–O). These results indicate that *35S::miRNVL5* plants were hypersensitive, whereas *35S::mGhCHR* or *35S::GhCHR* plants were tolerant to salt stress.

### *35S::miRNVL5 Arabidopsis* plants accumulated more Na^+^ than wild-type plants

**Figure 4:**
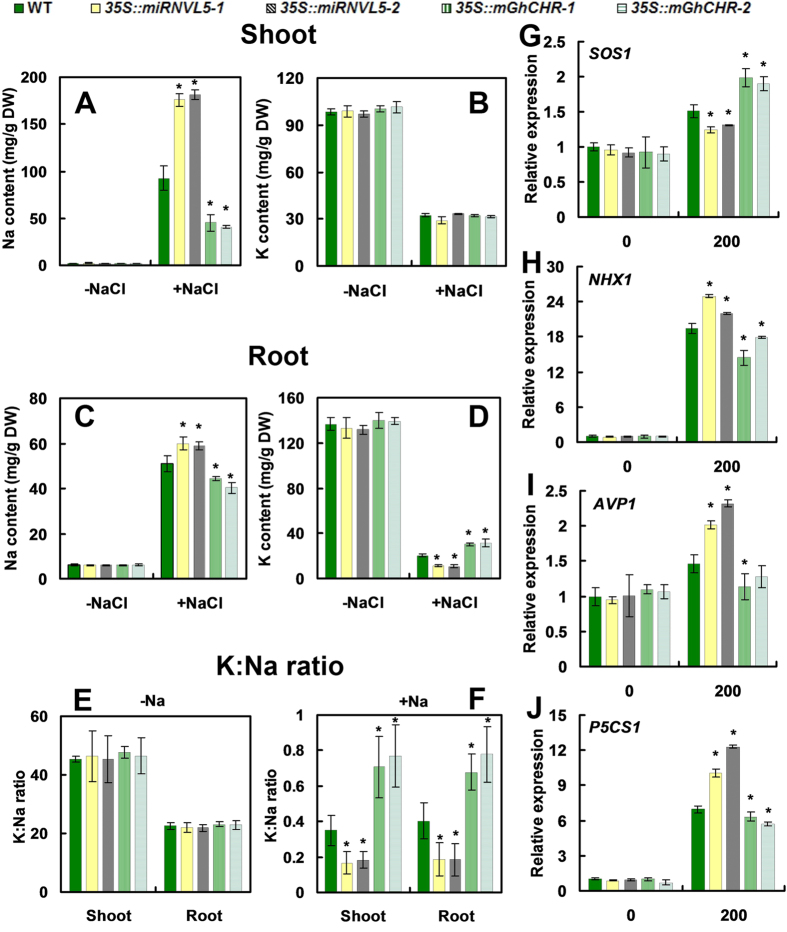
Sodium (Na^+^)/potassium (K^+^) accumulation and expression of *SOS1*, *NHX1*, *AVP1* and *P5CS1* in transgenic and wild-type (WT) plants exposed to NaCl. (**A,C**) Na^+^ in shoots and roots. (**B,D**) K^+^ in shoots and roots of three week-old WT, *35S::miRNVLU5* and *35S::mGhCHR* transgenic plants exposed to 200 mM NaCl exposure for 3 d. (**E,F**) K^+^/Na^+^ ratio in WT and the transgenic plants under −NaCl (**E**,**F**) and +NaCl (**F**) Condition. (**G**–**J**) qRT-PCR analysis of the indicated gene expression. Three week-old seedlings were exposed to 200 mM NaCl for 1 h. Vertical bars represent SD of the mean with three replicates. Asterisks indicate that mean values are significantly different between the transgenic plants and WT (*p* < 0.05).

Excess Na^+^ is toxic to plants, whereas K^+^ is antagonistic to Na^+^ under salt stress^3^. To identify the potential mechanism of transgenic plants in response to saline stress, concentrations of Na^+^ and K^+^ were assessed in both transgenic and WT plants. There was no difference of Na^+^ and K^+^ concentrations in wild-type and transgenic plants under normal condition (Fig. 4A–D). However, when exposed to 200 mM NaCl, the *35::miRNVL5* plants accumulated more Na^+^ in shoots and roots; conversely, the concentration of Na^+^ in the tissues was lower in *35S::mGhCHR* plants as compared to wild-type (Fig. 4A,C). In shoots, no difference of K^+^ concentration was observed between the transgenic plants and wild-type; but in roots, lower levels of K^+^ in *35::miRNVL5* plants and higher levels in *35S::mGhCHR* plants were determined. The imbalance of Na^+^ and K^+^ levels led to an increase in K^+^/Na^+^ ratio in *35S::mGhCHR* plants and decrease in K^+^/Na^+^ ratio in *35::miRNVL5* plants (Fig. 4E,F).

We further assessed transcripts of several genes responsible for plant tolerance to salt stress. *SOS1* (Salt Overly Sensitive 1) is a plasma membrane Na^+^/H^+^ antiporter and mediates Na^+^ efflux^29^. Compared to the control, expression of *SOS1* was lower in *35::miRNVL5* plants, whereas higher in *35S::mGhCHR* plants under salt stress (Fig. 4G). *NHX1* (Sodium Hydrogen Exchanger 1) encodes a vacuolar Na^+^/H^+^ antiporter involved in salt resistance and transports Na^+^ into the vacuole by using the electrochemical gradient of protons generated by the vacuolar H^+^ -translocating enzyme, H^+^ -adenosine triphosphatase and H^+^ -inorganic pyrophosphatase^30^. *AVP1* (Pyrophosphate-energized vacuolar membrane proton pump 1) encodes a H^+^ -translocating (pyrophosphate-energized) inorganic pyrophosphatase (H^+^ -PPase) located in the vacuolar membrane^31^. *P5CS1* (Delta-1-pyrroline-5-carboxylate synthase 1) is a rate-limiting enzyme in biosynthesis of proline, whose mRNA is induced by drought, salinity, and ABA^32^. qRT-PCR analysis showed that expression of *NHX1*, *AVP1* and *P5CS1* were highly expressed in *35::miRNVL5* plants, whereas those in *35S::mGhCHR* plants were inhibited (Fig. 4H–J).

### Identification of genes targeted by miRNVL5 in *Arabidopsis*

Because expression of miRNVL5 in *Arabidopsis* led to salt sensitive phenotypes, this allowed for hypothesis that some putative targets of miRNVL5 may exist in *Arabidopsis*. Based on the assumption, we predicted the target genes of miRNVL5 in *Arabidopsis*. According to the commonly accepted criteria, five putative target genes were identified. Interestingly, all these predicted genes belong to the PHD family protein genes (Table 1). We then analyzed their transcriptional abundance of the genes from our microarray datasets (WT + S/WT-S, miR5 − S/WT-S and miR5 + S/WT + S) and found all of them were repressed in the *35S::miRNVL5* plants under salt stress (Table 1; Data S5). To confirm the genes predicted, genome-wide high throughput degradome sequencing was performed based on the recently developed approach^33^,^34^. We sequenced three libraries including WT + S (wild-type with salt), MIR5 − S (*35S::miRNVL5* plants without salt treatment), and MIR5 + S (*35S::miRNVL5* plants treated with salt) from *Arabidopsis*. A total of 24.9–30.4 million short sequencing reads representing the 5’ ends, uncapped and polyadenylated RNAs were obtained (Data S6). After removal of low quality, error and redundant reads, 4.4–6.5 million unique reads were mapped to the *Arabidopsis* genome, which accounted for 70.6%, 83.6%, and 72.0% of the clean reads for the WT + S, MIR5 − S, and MIR + S libraries, respectively.

**Figure 5:**
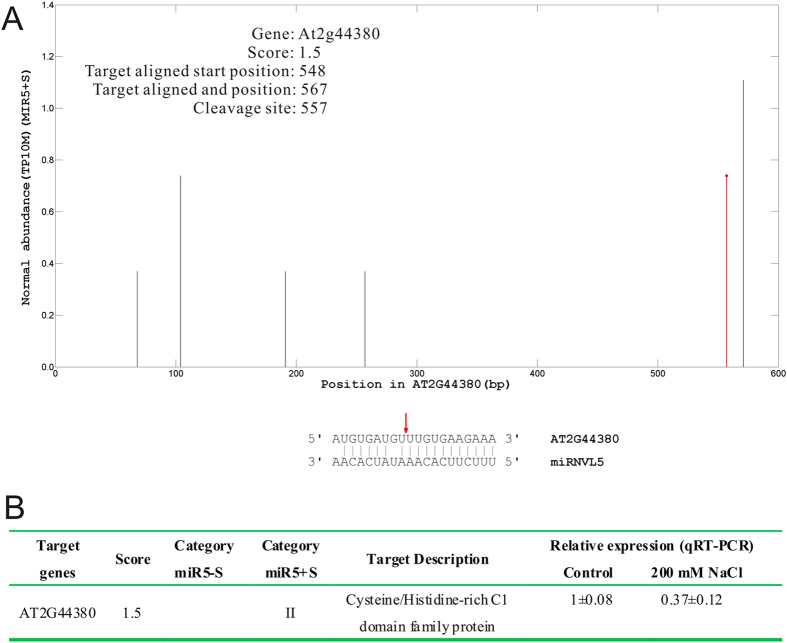
Identification of miRNVL5 target from Arabidopsis through degradome sequencing. (**A**) The red line with a dot indicates the significant signature. The arrow indicates the signature produced by miRNA-directed cleavage. (**B**) Target for miRNVL5 identified from degradome of *35S::miRNVL5* Arabidopsis exposed to minus salt (miR5 − S) and plus salt (miR5 + S). Three week-old Arabidopsis seedlings were treated with 0 (control) and 200 mM NaCl for 1 h and the treated seedlings were used for degradome sequencing and qRT-PCR.

miRNVL5 target was identified by using the CleaveLand pipeline^33^. According to the results, AT2G44380 has been identified as the target of miRNVL5 (Fig. 5A). The *t*-plot graphics showed that the target transcripts fall into miRNA-guided cleavage remnants; the alignment of the sequence manifested the signature that can be produced by miRNVL5-directed cleavage (Fig. 5B). Both microarray and qRT-PCR analyses showed that AT2G44380 in wild-type seedlings was induced by NaCl, but repressed by in *35S::miRNVL5* plants under salt stress (Table 1).

### Identification of downstream genes of AT2G44380 in *Arabidopsis*

**Figure 6:**
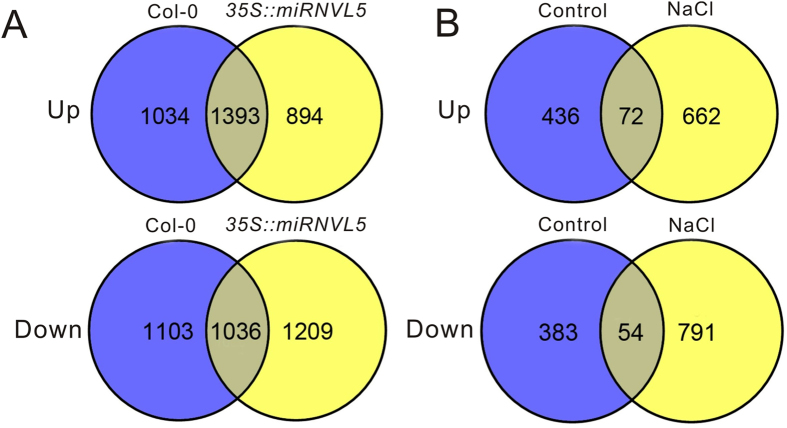
Differentially expressed genes in Arabidopsis wild-type (Col-0) and *35S::miRNVL5* seedlings under salt stress. Three weeks-old Arabidopsis seedlings were treated with 200 mM NaCl for 1 h. (**A**) Venn diagram showing up- and down-regulated genes in Col-0 and *35S::miRNVL5* seedlings under salt stress. (**B**) Venn diagram showing up- and down-regulated genes in *35S::miRNVL5* seedlings relative to Col-0 grown on the control (0 mM) and 200 mM NaCl media.

The expression of *miRNVL5* and *GhCHR* in *Arabidopsis* generating distinct phenotypes suggests that downstream genes responsible for salt stress tolerance could be regulated. To identify putative downstream genes in *Arabidopsis* under salt stress, we analyzed the global transcripts in *35S::miRNVL5* and wild-type plants under salt stress by generating an Agilent GeneChip with 43,803 distinct probes. By profiling the microarray, 1034 genes were induced and 1103 were repressed in wild-type, while 894 genes were induced and 1209 genes were repressed in *35S::miRNVL5* plants under salt stress (Fig. 6A; Data S5). Comparative analysis of transcripts between *35S::miRNVL5* and WT plants showed that 436 genes were induced and 383 genes were repressed under control condition, whereas 662 genes were induced and 791 genes were repressed under salt stress (Fig. 6B; Data S7).

The commonly repressed or induced genes were examined by screening the microarray datasets (WT + S/WT-S, miR5 − S/WT-S and miR5 + S/WT + S). Only 6 genes matched the criteria, including CBF1, CBF2, CBF3/DREB1A, ERF4, AT3G22920 and AT3G49200, and all of the genes were repressed (Table 2). CBF1/2/3 proteins are APETALA2/ethylene-responsive transcription factors that bind to DRE and activate the transcription of target stress-inducible genes to confer plant tolerance to abiotic stresses^35^. ERF4 encodes a ethylene-responsive transcription factor 4^36^. AT3G22920 and AT3G49200 encode peptidylprolyl isomerase and *O*-acyltransferase (WSD1-like) family protein, respectively. Our data showed that all six genes in WT were induced under NaCl exposure but repressed in *35S::miRNVL5* plants under either normal or NaCl stress condition. These results indicate that the genes may be directly or indirectly mediated by a putative component, which is in turn negatively regulated by miRNVL5 in *Arabidopsis*. Of the six genes, three were randomly selected for qRT-PCT validation, and all of them were well confirmed (Data S8).

### A group of salt-responsive genes were co-expressed with AT2G44380

**Figure 7:**
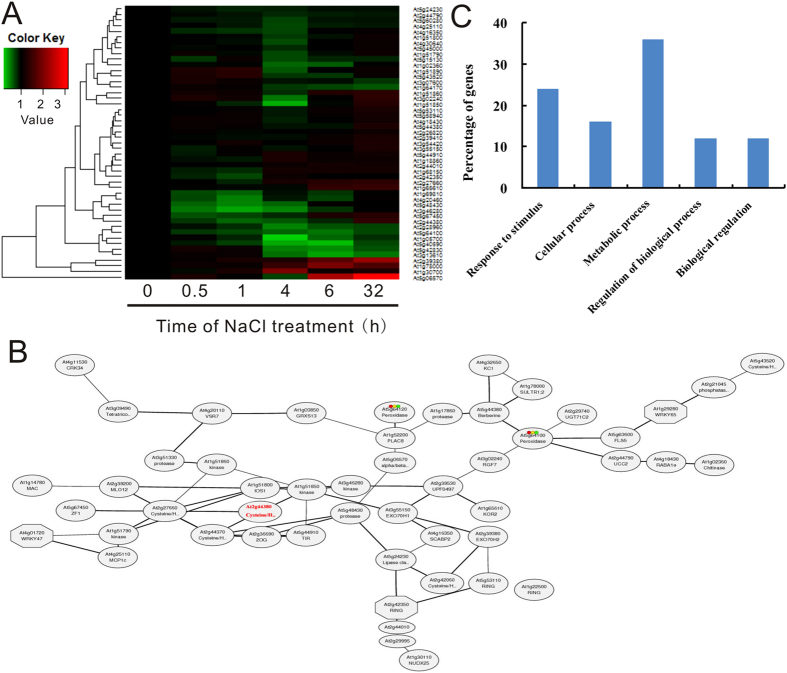
Co-expression, network and Gene Orthology (GO) analysis of salt-inducible genes in Arabidopsis. (**A**) Differentially expression profiles of salt-responsive genes that are co-expressed with AT2g44380 in Arabidopsis treated with 100 mM NaCl for 0–32 h. Hierarchial clustering display performed using Euclidean distance according to expression pattern with the MeV software. The intensities of the color represent the relative magnitude of fold changes in log values. The raw transcriptional data that the heatmap used were obtained from Li *et al.* (2009) and normalized using RMA algorithm (Dinneny, *et al.*, 2008) with Expression Console software (Affymetrix Technologies). (**B**) The hypothetic network under salt stress. (**C**) Gene Orthology analysis.

Recent studies have shown that many genes may have a similar expression pattern under a certain environmental stimuli, and in this case, they are usually working in a similar pathway^37^,^38^. By extraction of tightly co-expressed genes, one may find important or even novel genes associated with specific pathways. All salt-responsive genes were searched based on the Gene Orthology (GO) annotation in the *Arabidopsis* database (The Arabidopsis Information Resource, TAIR, http://www.arabidopsis.org/). A total of 49 genes that can co-express with AT2G44380 were identified (Data S9). These genes were further compared to the microarray datasets with salt stress^39^ (Fig. 7A). A strict screening criterion was set up, by which only genes with strong salt stress response could be considered as tightly co-expressed genes. Two genes (At2g42060 and At1g14780) were identified as core (or guide) genes that connect the remaining 56 genes. We took an advantage of the database ATTED-II, which used a unique co-expression measure, namely Mutual Rank (MR) of the Pearson’s correlation coefficient, to compare the co-expression strength for the guide gene AT2G44380^40^. With a strict cutoff (MR < 50), a gene network from the 50 co-expression genes was generated (Fig. 7B). These genes were found to be well associated. For instance, two genes At2g27660 and At2g44370 were predicted to interact with AT2G44380. The GO and KEGG (Kyoto Encyclopedia of Genes and Genomes) analyses revealed four diverse pathways with salt stress (Fig. 7C). Genes belonging to the Response to stimulus (24%) and metabolic process (36%) comprise the majority of the genes.

## Discussion

Although there are substantial miRNAs isolated from higher plants recently, only a few of them have been functionally characterized as regulators of plant response to salt stress^8^,^10^,^11^,^34^. In this study, we isolated and functionally characterized a new miRNA and its target from cotton. Sequence analysis revealed that the miRNVL5 precursor can fold into a perfect secondary hairpin structure, with higher negative minimal free energies (MFEs) and minimal free energy index (MFEIs)^41^. The target of miRNVL5 was predicted and experimentally confirmed by 5′-RACE analysis. *In silico* analysis revealed that *GhCHR* belongs to the Cys/His-rich DC1/PHD domains-containing protein gene family. Although miRNVL5 was isolated from cotton ovules, both mature and primary miRNVL5 are ubiquitously expressed in cotton plants. *GhCHR* expressed in the same tissues where miRNVL5 expressed, but its expression level was lower, indicating that *GhCHR* is constantly under the control of miRNVL5. miRNVL5-regulated *GhCHR* expression was also detected under salt stress, but both miRNVL5 and pri-miRNVL5 expression was repressed in cotton with concomitant higher expression levels of *GhCHR* in the same tissues. Such an expression pattern suggests that *GhCHR* may serve as a positive regulator of plant salt stress response.

Our data shows that ectopic expression of *GhCHR* in *Arabidopsis* promoted seed germination, seedling growth and primary root elongation under salt stress. Furthermore, *35S::mGhCHR* accumulated less Na^+^ in shoots as compared to wild-type. Although there was no change of K^+^ concentration in shoots, the K^+^/Na^+^ ratio was higher in *35S::mGhCHR* shoots. The higher ratio of K^+^/Na^+^ was maintained in shoots possibly owning to the exclusion mechanism, because *SOS1* responsible for Na^+^ pumping-out was activated in the *35S::mGhCHR* plants. In contrast, expression of *NHX1* which is encoding Na^+^/H^+^ antiporter in vacuoles was repressed in *35S::mGhCHR* plants, suggesting that sequestration of higher Na^+^ in vacuoles under the condition may not be the mechanism for salt tolerance. A similar expression pattern for *AVP1* was observed in the *35S::mGhCHR* plants. *P5CS1* can be considered as a marker gene responding to salt stress^42^. The lower level of *P5CS1* suggests that expression of *GhCHR* in *Arabidopsis* attenuated degree of salt stress.

One striking observation in this study is that the expression of miRNVL5 in *Arabidopsis* led to the sensitive phenotypes of *35S::miRNVL5* plants to sodium ions. Such an observation allowed for assumption that there are putative transcripts of genes possibly targeted by miRNVL5 in *Arabidopsis*. To identify the putative target of miRNVL5, degradome from wild-type and *35::miRNVL5* plants under control and salt stress was profiled. Degradome is a newly developed and highly efficient approach for identifying global authentic miRNA targets, which has been applied to many plant species^33^,^34^,^43^. Our analysis showed that among the several candidate targets, At2g44380 is most likely the target of miRNVL5. Several lines of evidence are given as follows: (1) there is a perfect target sit of miRNVL5 in At2g44380 mRNA; (2) At2g44380 encodes a cysteine/histidine-rich C1 domain family protein similar to GhCHR; and (3) Co-expression analysis showed that At2g44380 acts as a core gene capable of connecting several other genes in the networks.

We attempted to identify orthologs of miRNVL5 in *Arabidopsis*. A query search was made for all miRNAs from *Arabidopsis* and other plant species that have been so far discovered in miRNA database (http://www.mirbase.org/) and NCBI (http://www.ncbi.nlm.nih.gov/). Unfortunately, no confident miRNAs from *Arabidopsis* compatible to miRNVL5 have been detected. However, we cannot rule out the possibility of existing of putative miRNAs, because our studies have shown that although there was a similar phenotype of *35S::GhCHR* and *35S::mGhCHR* plants, and both transgenic plants have a similar level of *GhCHR* transcripts, the relative lower level of *GhCHR* transcripts was observed in *35S::GhCHR* plants. This suggests that some putative miRNAs may regulate the *GhCHR* abundance in a negative way. *Gossypium hirsutum* is a tetraploid crop with four copies of chromosomes. The extra copies of genetic materials and/or increased levels of heterozygosity in two or more sets of chromosome are predicted to be advantageous for selection, adaption and domestication^44^. Polyploidy induces non-additive expression of homologous genes through mechanisms of transcriptional regulation via chromatin modifications and DNA methylation^45^,^46^,^47^ or posttranscriptional regulation involving small RNA regulation^48^. The transcriptional or posttranscriptional regulation divergence between *GhCHR* and At2g44380 may be a good example of the miRNA-mediated mechanisms for evolutionary differences of duplicate genes in polyploids. Meanwhile, miRNVL5 in *Arabidopsis* might be lost during the course of evolution.

Our microarray analysis showed that miRNVL5 expression in *Arabidopsis* altered expression of many genes under normal and salt stress conditions. It was interesting to identify the downstream genes of At2g44380 in *Arabidopsis*. There were more down-regulated genes detected in *35S::miRNVL5* seedlings than those in wild-type. An array of genes could be repressed by miRNVL5 expression in *Arabidopsis* under salt stress. We further narrowed down the number of genes differentially expressed in the three datasets (WT + S/WT-S, miR5 − S/WT-S and miR5 + S/WT + S). Only 6 genes (*CBF1*, *CBF2*, *CBF3*, *ERF4*, AT3G22920 and AT3G49200) remained after most of genes were filtered out. All these genes are stress-responsive genes. *CBFs* encode transcriptional activators that control the expression of genes with the CRT/DRE-responsive element in their promoters and are rapidly induced in response to low temperature^49^. Evidence also demonstrates that the *CBFs-*family genes play an important role in activating gene expression during salt stress^35^,^50^. Overexpression of the DREB1/CBF family genes increased tolerance to freezing, drought, and high salinity in transgenic *Arabidopsis* plants^51^. Ethylene-responsive transcription factor 4 (ERF4) is known as one of ethylene-responsive element binding proteins (EREBPs) that interacts with the GCC-box sequence with the core GCCGCC motif 52. Expression of *AtERF4* can be induced by ethylene, jasmonic acid (JA), ABA and salt stress^36^,^39^. Overexpression of ERF4 from *Brissica rapa* increased tolerance to salt and drought stresses in *Arabidopsis*^53^. In this study, *CBF1*, *CBF2*, *CBF3* and *ERF4* were found to be repressed in the transgenic *Arabidopsis* plants expressing miRNVL5, which shows a phenotype of NaCl sensitivity. These results suggest that miRNVL5-regulated repression of *CBF1*, *CBF2*, *CBF3* and *ERF4* should be responsible for plant sensitivity to the salinity stress.

**Figure 8:**
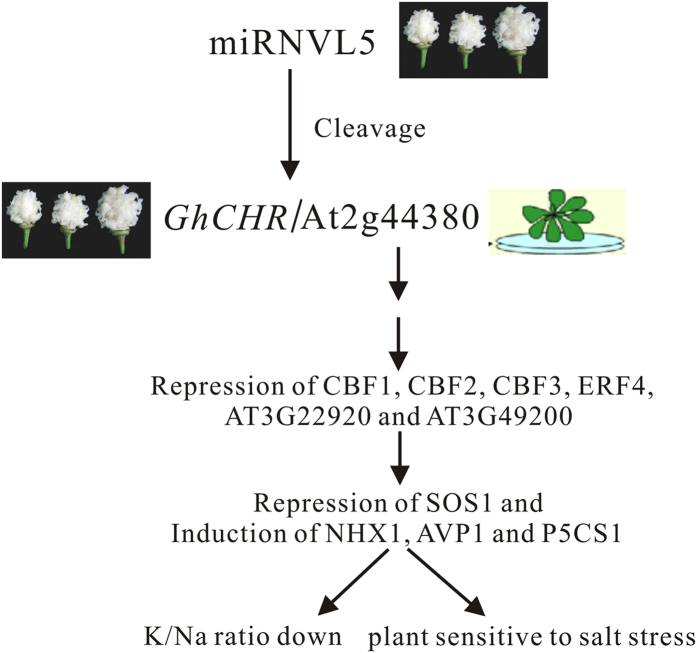
The model for cotton miRNVL5 regulating its target genes *GhCHR* (cotton) and At2g44380 (Arabidopsis). The schematic model reflects that transformation of miRNVL5 into Arabidopsis results in decrease in K/Na ratio and seedling sensitivity to salt stress possibly through repressing At2g44380, and several other downstream genes *CBF1*, *CBF2*, *CBF3*, *ERF4*, *AT3G22920*, *AT3G49200* and others.

Taken together, the present study showed that miRNVL5 and *GhCHR* were involved in plant response to salt stress. Ectopic expression of miRNVL5 resulted in NaCl sensitive phenotype in *Arabidopsis*, whereas a tolerance phenotype was observed in the transgenic *Arabidopsis* expressing *GhCHR*. By degradome analysis, a *GhCHR* orthorlous gene At2g44380 has been identified in *Arabidopsis* as a target of miRNVL5. Whether At2g44380 is involved in regulation of plant tolerance to salt stress remains to be further investigated. Based on our observation, a simple model could be proposed of how miRNVL5 regulates plant response to salt stress (Fig. 8).

## Materials and Methods

### Plant materials and stress treatment

Upland cotton (*Gossypium hirsutum,* cv. Xuzhou 142, wild type, WT) plants were grown in the field at the Nanjing Agricultural University Experimental Station. Flowers were tagged and ovules were sampled and dissected from −2 to 20 DPA (day of anthesis) ovaries in the early morning as described in our previous report^13^. The ovules were frozen immediately in liquid nitrogen and stored at −80 °C till to analysis.

For stress treatments, cotton seeds were germinated on 1/2 MS medium under a 16 h light/8 h dark cycle at 28 °C. Two or three week-old seedlings were treated by different concentrations of NaCl (0–400 mM) in 1/2 MS liquid medium for 0–6 h^54^. After treatment, shoots and roots of seedlings were separately harvested for RNA isolation. *Arabidopsis thaliana* ecotype Col-0 seeds were surface sterilized and germinated on 1/2 MS medium. Seeds were first stratified for 3d at 4 °C in the dark (to break dormancy) and then germinated in a growth chamber (22 °C, 16 h photoperiod). After 2 weeks, seedlings were used for NaCl treatment and analysis as described as the same for cotton treatments.

### *GhCHR* cloning and identification of miRNVL5 cleavage site in *GhCHR* sequence

Two pairs of specific primers were designed for *GhCHR* cDNA cloning and used for amplification based on the consensus sequence from cotton leaves (Data S10). The PCR thermocycling conditions were as follow: 94 °C for 5 min, followed by 35 cycles of 94 °C for 1 min, 57 °C 30s, and 72 °C for 2 min, and a final extension at 72 °C for 10 min. The PCR product was separated by electrophoresis on a 1% agarose gel stained with ethidium bromide, purified using the Quiquick gel extraction kit (BioDev Biotech, Beijing, China). The product was then cloned into the PBI121 vector, and transformed into *E. coli* in JM109. The predicted target *GhCHR* of miRNVL5 was validated with the 5′-RACE assay using the FirstChoice RLM-RACE kit (Ambion)^55^. RNAs were prepared from cotton ovules. The RNA was ligated with a 5′ RNA adapter and a RT-PCR was performed. The resulting cDNA was used as template for PCR amplification. The amplified products were gel purified, cloned and sequenced.

### RT-PCR analysis

RNA was extracted using the plant RNAout Kit (Tiandz) according to our previous reports^13^,^56^. All RNA samples were quantified and examined by a Nanodrop ND 1000 spectrophotometer. The first strand cDNA was synthesized from 1.0 μg total RNA by Moloney Murine Leukemia Virus Reverse Transcriptase (Promega) using oligo(dT) primers. The qRT-PCR was performed with a MyiQ Single Color Real-time PCR system (Bio-Rad) in a final volume of 20 μL containing 2 μL of a 1/10 dilution of cDNA in water, 10 μL of the 2 × SYBR Premix Ex Taq (TaKaRa) and 200 nM of forward and reverse primers. The thermal cycling conditions were 40 cycles of 95 °C for 5 s for denaturation and 60 °C for 30 s for annealing and extension. All reactions were run in triplicate. PCR efficiency was determined by a series of 2-fold dilutions of cDNAs. The calculated efficiency of all primers was 0.9 to 1.0. Cotton gene gbpolyubiquitin-2 (EE592464) was used as a normalizer and relative expression levels of genes were presented by 2^−△CT^ (Data S10).

Expression of *GhCHR* and *pri-miRNVL5* was detected by quantitative real-time reverse transcriptase RT-PCR (qRT-PCR) using the fluorescent intercalating dye SYBR-Green in a detection system (MJ Research, Opticon 2) according to a previous report^57^. A two-step qRT-PCR procedure was performed. First, total RNA samples (1.0 μg per reaction) from roots, cotyledons, hypocotyls, petals, ovules and fibers were reversely transcribed into cDNAs. Then, the cDNAs were used as templates in qRT-PCR with gene-specific primers (Data S10). Semi-quantitative RT-PCR was also used to analyze gene expression of *GhCHR*. The gene specific primer pairs (Data S10) were used for PCR reactions under the following conditions: pre-denaturation at 94 °C for 5 min, followed by 30 cycles of 30 s at 94 °C, 30 s at a specific annealing temperature (57 °C), and 30 s at 72 °C. Cotton *actin2*, an internal control for constitutive expression, was uniformly expressed in all tissues examined. As an internal control and to exclude genomic contamination, *Actin2* was amplified (same cycling conditions as above for 28 cycles) from the same cDNA samples.

For qRT-PCR analysis of mature miRNVL5, total RNA isolated by TRIzol reagents (Invitrogen) was pre-treated with DNase following thein manufacturer’s instructions. Mature miRNVL5 were detected by stem-loop qRT-PCR^58^. The precursor miRNVL5 (pri-miRNVL5) was analyzed by the methods of Song *et al.*^11^. Transcript analysis of its target gene was performed also using qRT-PCR. The primers used for qRT-PCR are presented in Supplementary Data S10.

### Transformation of *GhCHR* and *pri-miRNVL5* into *Arabidopsis*

The *GhCHR* and *Pri-miRNVL5* genomic sequences were inserted into downstream of the CaMV35s promoter in the binary vector pBI121^11^. The constructs were then transferred into *Agrobacterium tumefaciens* for *Arabidopsis* transformation by the floral dip method^11^. Transgenic lines were selected on the 1/2 MS medium with 50 mg/L kanamycin. Independent transgenic lines were obtained, and PCR was performed to verify the presence of *35S::(m)GhCHR* and *35S::miRNVL5* in these transgenic *Arabidopsis* plants.

### Degradome sequencing and data analysis in *35::miRNVL5 Arabidopsis*

Degradome libraries were constructed based on a previous method^34^. Briefly, poly (A) RNA was extracted from total RNA sample using the Oligotex kit (Qiagen). Polyadenylated transcripts possessing 5′ monophosphates were ligated to RNA adapters consisting of a MmeI recognition site at its 3′ end. After ligation, the first-strand cDNA was generated using oligo d(T) and amplified using five PCR cycles. The PCR product was purified and digested with MmeI. The digested PCR product was then ligated to a 3′ double DNA adapter, amplified by 20 PCR cycles, and gel-purified for Illumina sequencing.

Sequenced tags with 18–21 nucleotides long were normalized after trimming sequence adapters and filtering the low quality tags. The sliced miRNA targets were identified and classified based on the CleaveLand pipeline^33^. The unique reads were normalized to give reads per million and subsequently mapped to annotated cDNA sequences from the Gene Index database (ftp://occams.dfci.harvard.edu/pub/bio/tgi/data/Brassica_napus) (BnGI release 5.0).

### Microarray hybridization and data analysis

Three week-old *Arabidopsis* seedlings were treated with 200 mM NaCl for 1 h and harvested. Total RNA was extracted using mirVanaTM RNA Isolation Kit (Applied Biosystem p/n AM1556). The extracted RNA was quantified by NanoDrop ND-2000 (Thermo Scientific). RNA integrity was assessed using Agilent Bioanalyzer 2100 (Agilent Technologies). Total RNA was dephosphorylated, denaturated and labeled with Cyanine-3-CTP. After purification, the labeled RNA was hybridized onto the microarray (Agilent Technologies). The arrays were scanned with the Agilent Scanner G2505C (Agilent Technologies). Feature Extraction software (version10.7.1.1, Agilent Technologies) was used to analyze array images to get raw data. The Genspring software (version 12.5; Agilent Technologies) was employed to analyze raw data, which were normalized with the quantile algorithm. The probes that at least 1 condition out of 2 conditions has flags in “Detected” were chosen for further data analysis. Differentially expressed genes were identified through fold change as well as *P* value calculated using *t*-test. The threshold set for up- and down-regulated genes was a fold change ≥ 2.0 and a P value ≤ 0.05.

### Measurement of Na^+^ and K^+^ concentrations

Three week-old *Arabidopsis* seedlings of transgenic and wild-type plants grown on 1/2 MS medium containing 200 mM NaCl for 3 d were harvested and dried at 80 °C, and digested with the mixture of nitric acid and hydrogen peroxide using microwave system (MARS, CEM). The digested samples were used to quantify Na^+^ and K^+^ using inductively coupled plasma-atomic emission spectrometry (ICP-AES) (Optimal 2100DV, Perkin Elmer Instruments).

### Construction of *Arabidopsis* gene co-expression networks relevant to salt stress

Gene co-expression networks were constructed with the analytical tool of ATTED-II (http://atted.jp/), from which the relationship of genes from *Arabidopsis* was analyzed based on all microarray datasets^38^,^40^. Distance relationship was defined by MR (Mutual Rank) value. The co-expression of genes was analyzed with CoExSearch in ATTED-II. Usually, when MR is <50, the correlation of genes is considered as “close”; however, when MR is >1000, the relationship between two genes is very “weak”. In this study, genes with MR <1000 were considered as co-expression genes.

### Statistical analysis

Experiments were independently performed in triplicate. Each result shown in the figures was the mean of three biological replicates and each treatment contained 12–40 seedlings. All transgenic (T4 homozygous) seedlings were used in the study. The significant differences between treatments were statistically evaluated by standard deviation and one-way analysis of variance (ANOVA). The data between differently treated groups were compared statistically by ANOVA followed by the least significant difference (LSD) test (*P* < 0.05).

## Additional Information

**How to cite this article**: Gao, S. *et al.* A cotton miRNA is involved in regulation of plant response to salt stress. *Sci. Rep.* **6**, 19736; doi: 10.1038/srep19736 (2016).

## Supplementary Material

Supplementary Data

## Figures and Tables

**Table 1:** Prediction of miRNVL5 targeted genes from Arabidopsis and expression pattern in wild-type and *35S::miR5* (miR5) plants under salt stress.

Gene ID	Expectation value	Inhibition type	WT + S/WT-S Fold Change	miR5 − S/WT-S Fold Change	miR5 + S/WT + S Fold Change
AT2G44380	1.5	Cleavage	2.20	−1.07	−1.21
AT4G39070	2.5	Cleavage	1.31	−1.03	−1.01
AT4G01930	3	Cleavage	1.28	−1.06	−1.09
AT2G19260	3	Translation	1.13	1.12	−1.27
AT2G21850	3	Translation	-1.03	−1.11	−1.16

The prediction was run according to the online tool (http://plantgrn.noble.org/psRNATarget/). The threshold was set as 3.

**Table 2:** List of genes that are commonly induced in Arabidopsis Col-0 (wild-type, WT) and *35S::miRNVL5* (miR5) seedlings under salt (S, 200 mM NaCl) stress.

AGI ID	Description	WT + S/WT-S	miR5 − S/WT-S	miR5 + S/WT + S
CBF1	Dehydration-responsive element-binding protein 1B	2.26	−3.93	−1.07
CBF2	Dehydration-responsive element-binding protein 1C	2.37	−2.67	−1.10
CBF3 (DREB1A)	Dehydration-responsive element-binding protein 1A	20.75	−2.18	−1.04
ERF4	Ethylene-responsive transcription factor 4	3.18	−3.53	−1.32
AT3G22920	Peptidylprolyl isomerase	2.76	−2.47	−1.32
AT3G49200	O-acyltransferase (WSD1-like) family protein	2.97	−6.57	−1.23

Genes presented showed a fold change more than 2.0. Transcript abundance in Col-0 and 35S::miRNVL5 seedlings based on microarray analysis.

## References

[b1] Türkan I. & Demiral T. Recent developments in understanding salinity tolerance. Environ. Exp. Bot. 67, 2–9 (2009).

[b2] Munns R. & Tester M. Mechanisms of salinity tolerance. Annu. Rev. Plant Biol. 59, 651–681 (2008). 18444910 10.1146/annurev.arplant.59.032607.092911

[b3] Zhu J. K. Regulation of ion homeostasis under salt stress. Curr. Opin. Plant Biol. 6, 441–445 (2003). 12972044 10.1016/s1369-5266(03)00085-2

[b4] De Costa W., Zorb C., Hautung W. & Schubert S. Salt resistance is determined by osmotic adjustment and abscisic acid in newly developed maize hybrids in the first phase of salt stress. Physiol. Plant 131, 311–321 (2007). 18251902 10.1111/j.1399-3054.2007.00962.x

[b5] Jones-Rhoades M. W. & Bartel D. P. Computational identification of plant microRNAs and their targets, including a stress-induced miRNA. Mol. Cell, 14, 787–799 (2004). 15200956 10.1016/j.molcel.2004.05.027

[b6] Phillips J. R., Dalmay T. & Bartels D. The role of small RNAs in abiotic stress. FEBS Lett. 581, 3592–3597 (2007). 17451688 10.1016/j.febslet.2007.04.007

[b7] Khraiwesh B., Zhu J. K. & Zhu J. H. Role of miRNAs and siRNAs in biotic and abiotic stress responses of plants. Biochim. Biophys. Acta. 1819, 137–148 (2012). 21605713 10.1016/j.bbagrm.2011.05.001 PMC3175014

[b8] Zhang B. H. MicroRNA: a new target for improving plant tolerance to abiotic stress. J. Exp. Bot. 66, 1749–1761 (2015). 25697792 10.1093/jxb/erv013 PMC4669559

[b9] Zhou M. & Luo H. Role of microRNA319 in creeping bentgrass salinity and drought stress response. Plant Signal. Behav. 9, e28700 (2014). 10.4161/psb.28700 PMC4091478 24698809

[b10] Gao P. *et al.* Over-expression of osa-MIR396c decreases salt and alkali stress tolerance. Planta 231, 991–1001 (2010). 20135324 10.1007/s00425-010-1104-2

[b11] Song J. B. *et al.* miR394 and LCR are involved in Arabidopsis salt and drought stress responses in an abscisic acid-dependent manner. BMC Plant Biology 13, 210 (2013). 24330668 10.1186/1471-2229-13-210 PMC3870963

[b12] Abdurakhmonov I. Y. *et al.* Small RNA regulation of ovule development in the cotton plant, *G. hirsutum* L. BMC Plant Biol. 8, 93 (2008). 18793449 10.1186/1471-2229-8-93 PMC2564936

[b13] Kwak P. B., Wang Q. Q., Chen X. S., Qiu C. X. & Yang Z. M. Enrichment of a set of microRNAs during the cotton fiber development. BMC Genomics 10, 457 (2009). 19788742 10.1186/1471-2164-10-457 PMC2760587

[b14] Pang M. *et al.* Genome-wide analysis reveals rapid and dynamic changes in miRNA and siRNA sequence and expression during ovule and fiber development in allotetraploid cotton (*Gossypium hirsutum* L.). Genome Biol. 10, R122 (2009). 19889219 10.1186/gb-2009-10-11-r122 PMC3091316

[b15] Ruan M. B., Zhao Y. T., Meng Z. H., Wang X. H. & Yang W. C. Conserved miRNA analysis in *Gossypium hirsutum* through small RNA sequencing. Genomics 94, 263–268 (2009). 19628031 10.1016/j.ygeno.2009.07.002

[b16] Wang M., Wang Q. & Zhang B. Response of miRNAs and their targets to salt and drought stresses in cotton (*Gossypium hirsutum* L.). Gene 530, 26–32 (2013). 23948080 10.1016/j.gene.2013.08.009

[b17] Xie F., Jones D. C., Wang Q., Sun R. & Zhang B. Small RNA sequencing identifies miRNA roles in ovule and fibre development. Plant Biotechnol. J. 13, 355–369 (2015). 25572837 10.1111/pbi.12296

[b18] Guan X. *et al.* miR828 and miR858 regulate homoeologous *MYB2* gene functions in *Arabidopsis* trichome and cotton fibre development. Nat. Commun. 5, 3050 (2014). 24430011 10.1038/ncomms4050

[b19] Li G. *et al.* Two cotton Cys2/His2-type zinc-finger proteins, GhDi19-1 and GhDi19-2, are involved in plant response to salt/drought stress and abscisic acid signaling. Plant Mol. Biol. 74, 437–452 (2010). 20852918 10.1007/s11103-010-9684-6

[b20] Li C. *et al.* TaCHP: a wheat zinc finger protein gene down-regulated by absicisic acid and salinity stress plays a positive role in stress tolerance. Plant Physiol. 154, 211–221 (2012). 20639406 10.1104/pp.110.161182 PMC2938152

[b21] Devi S. J. S. R. *et al.* Identification of abiotic stress miRNA transcription factor binding motifs (TFBMs) in rice. Gene 531, 15–22 (2013). 23994683 10.1016/j.gene.2013.08.060

[b22] Takatsuji H. Zinc-finger protein, the classical zinc finger emerges in contemporary plant science. Plant Mol. Biol. 39, 1073–1078 (1999). 10380795 10.1023/a:1006184519697

[b23] Li W. T., He M., Wang J. & Wang Y. P. Zinc finger protein (ZFP) in plants-A review. Plant Omics 6, 474–480 (2013).

[b24] Bienz M. The PHD finger, a nuclear protein-interaction domain. Trends Biochem. Sci. 31, 35–40 (2006). 16297627 10.1016/j.tibs.2005.11.001

[b25] Sung S., Schmitz R. & Amasino R. M. A. PHD finger protein involved in both the vernalization and photoperiod pathways in Arabidopsis. Gene Dev. 20, 3244–3248 (2006). 17114575 10.1101/gad.1493306 PMC1686601

[b26] Greb T. *et al.* The PHD finger protein VRN5 functions in the epigenetic silencing of Arabidopsis FLC. Curr. Biol. 17, 73–78 (2007). 17174094 10.1016/j.cub.2006.11.052

[b27] Wei W. *et al.* Soybean GmPHD-type transcription regulators improve stress tolerance in transgenic Arabidopsis plants. PLoS One 4, e7209 (2009). 19789627 10.1371/journal.pone.0007209 PMC2747011

[b28] Wu T. *et al.* GmPHD5 acts as an important regulator for crosstalk between histone H3K4 di-methylation and H3K14 acetylation in response to salinity stress in soybean. BMC Plant Biol. 11, 178 (2011). 22168212 10.1186/1471-2229-11-178 PMC3288756

[b29] Shi H., Ishitani M., Kim C. & Zhu J. K. The Arabidopsis thalinia salt tolerance gene SOS1 encodes aputative Na^+^/H^+^ antiporter. Proc. Natl. Acad. Sci. USA 97, 6896–6901 (2000). 10823923 10.1073/pnas.120170197 PMC18772

[b30] Apse M. P., Aharon G. S., Snedden W. A. & Blmwald E. Salt tolerance conferred by overexpression of a vacuolar Na^+^/H^+^ antiport in Arabidopsis. Science 285, 1256–1258 (1999). 10455050 10.1126/science.285.5431.1256

[b31] Gaxiola R. A., Li J., Undurraga S., Dang L. M. & Allen G. J. Drought- and salt-tolerant plants result from overexpression of the AVP1 H^+^ -pump. Proc. Natl. Acad. Sci. USA 98 11444–11449 (2001). 11572991 10.1073/pnas.191389398 PMC58749

[b32] Zhu J. K. Cell signaling under salt, water and cold stresses. Curr. Opin. Plant Biol. 4, 401–406 (2001). 11597497 10.1016/s1369-5266(00)00192-8

[b33] Addo-Quaye C., Eshoo T. W., Bartel D. P. & Axtell M. J. Endogenous siRNA and miRNA targets identified by sequencing of the *Arabidopsis* degradome. Curr. Biol. 18, 758–762 (2008). 18472421 10.1016/j.cub.2008.04.042 PMC2583427

[b34] Zhou Z. S., Song J. B. & Yang Z. M. Genome-wide identification of *Brassica napus* microRNAs and their targets reveals their differential regulation cadmium. J. Exp. Bot. 59, 3443–3452 (2012). 10.1093/jxb/ers136 PMC3421990 22760473

[b35] Akhtar M. *et al.* DREB1/CBF transcription factors: their structure, function and role in abiotic stress tolerance in plants. J. Genet. 91, 385–395 (2012). 23271026 10.1007/s12041-012-0201-3

[b36] Yang Z., Tian L., Latoszek-Green M., Brown D. & Wu K. Arabidopsis ERF4 is a transcriptional repressor capable of modulating ethylene and abscisic acid responses. Plant Mol. Biol. 58, 585–596 (2005). 16021341 10.1007/s11103-005-7294-5

[b37] Fu F. F. & Xue H. W. Coexpression analysis identifies rice starch regulator1, a rice AP2/EREBP family transcription factor, as a novel rice starch biosynthesis regulator. Plant Physiol. 154, 927–938 (2010). 20713616 10.1104/pp.110.159517 PMC2949045

[b38] Li H., Wang L. & Yang Z. M. Co-expression analysis reveals a group of genes potentially involved in regulation of plant response to iron-deficiency. Gene 554, 16–24 (2015). 25300251 10.1016/j.gene.2014.10.004

[b39] Dinneny J. R. *et al.* Cell identity mediates the response of Arabidopsis roots to abiotic stress. Science 320, 942–945 (2008). 18436742 10.1126/science.1153795

[b40] Obayashi T. & Kinoshita K. Rank of correlation coefficient as a comparable measure for biological significance of gene coexpression. DNA Res. 16, 249–260 (2009). 19767600 10.1093/dnares/dsp016 PMC2762411

[b41] Zhang B. H., Pan X., Cannon C. H., Cobb G. P. & Anderson T. A. Conservation and divergence of plant microRNA genes. Plant J. 46, 243–259 (2006). 16623887 10.1111/j.1365-313X.2006.02697.x

[b42] Szekely G. *et al.* Duplicated P5CS genes of Arabidopsis play distinct roles in stress regulation and developmental control of proline biosynthesis. Plant J. 53, 11–28 (2008). 17971042 10.1111/j.1365-313X.2007.03318.x

[b43] Liu N. *et al.* Small RNA and degradome profiling reveals a role for miRNAs and their targets in the developing fibers of Gossypium barbadense. Plant J. 80, 331–344 (2014). 25131375 10.1111/tpj.12636

[b44] Leitch A. R. & Leitch I. J. Genomic plasticity and the diversity of polyploidy plants. Science 320, 481–483 (2008). 18436776 10.1126/science.1153585

[b45] Chen Z. J. Genetic and epigenetic mechanisms for gene expression and phenotypic variation in plant polyploids. Annu. Rev. Plant Biol. 58, 377–406 (2007). 17280525 10.1146/annurev.arplant.58.032806.103835 PMC1949485

[b46] Chen M., Ha M., Lackey E., Wang J. & Chen Z. J. RNAi of met1 reduces DNA methylation and induces genome-specific changes in gene expression and centromeric small RNA accumulation in *Arabidopsis* allopolyploids. Genetics 178, 1845–1858 (2008). 18430920 10.1534/genetics.107.086272 PMC2323781

[b47] Ha M., Ng D. W., Li W. H. & Chen Z. J. Coordinated histone modifications are associated with gene expression variation within and between species. Genome Res. 21, 590–598 (2011). 21324879 10.1101/gr.116467.110 PMC3065706

[b48] Ng D. W., Lu J. & Chen Z. J. Big roles for small RNAs in polyploidy, hybrid vigor, and hybrid incompatibility. Curr. Opin Plant Biol. 15, 154–161 (2012). 22326630 10.1016/j.pbi.2012.01.007

[b49] Stockinger E. J., Gilmour S. J. & Thomashow M. F. Arabidopsis thaliana CBF1 encodes an AP2 domain-containing transcriptional activator that binds to the C-repeat/DRE, a cis-acting DNA regulatory element that stimulates transcription in response to low temperature and water deficit. Proc. Natl. Acad. Sci. USA 94, 1035–1040 (1997). 9023378 10.1073/pnas.94.3.1035 PMC19635

[b50] Novillo F., Medina J., Rodriguez-Franco M., Neuhaus G. & Salinas J. Genetic analysis reveals a complex regulatory network modulating CBF gene expression and Arabidopsis to abiotic stress. J. Exp. Bot. 63, 293–304 (2012). 21940717 10.1093/jxb/err279 PMC3245470

[b51] Jaglo-Ottosen K. R., Gilmour S. J., Zarka D. G., Schabenberger O. & Thomsshow M. F. Arabidopsis CBF1 overexpression induceds COR genes and enhances freezing tolerance. Science 280, 104–106 (1998). 9525853 10.1126/science.280.5360.104

[b52] Ohme-Takagi M. & Shinshi H. Ethylene-inducible DNA binding proteins that interact with an ethylene-responsive element. Plant Cell 7, 173–182 (1995). 7756828 10.1105/tpc.7.2.173 PMC160773

[b53] Seo Y. J. *et al.* Overexpresion of the ethylene-responsive factor gene BrERF4 from Brassica rapa increases tolerance to salt and drought in Arabidopsis plants. Mol. Cell 30, 271–277 (2010). 10.1007/s10059-010-0114-z 20803085

[b54] Chen J. Y. & Dai X. F. Cloning and characterization of the *Gossypium hirsutum* major latex protein gene and functional analysis in *Arabidopsis thaliana*. Planta 231, 861–873 (2010). 20049612 10.1007/s00425-009-1092-2

[b55] Huang S. Q. *et al.* A set of miRNAs from *Brassica napus* in response to sulfate-deficiency and cadmium stress. Plant Biotechnol. J. 8, 887–899 (2010). 20444207 10.1111/j.1467-7652.2010.00517.x

[b56] Wang Q. Q. *et al.* Transcriptome profiling of early developing cotton fibre by deep-sequencing reveals significantly differential expression of genes in a fuzzless/lintless mutant. Genomics 96, 369–376 (2010). 20828606 10.1016/j.ygeno.2010.08.009

[b57] Li X. B., Fan X. P., Wang X. L., Cai L. & Yang W. C. The cotton ACTIN1 gene is functionally expressed in fibers and participates in fiber elongation. Plant Cell 17, 859–875 (2005). 15722467 10.1105/tpc.104.029629 PMC1069704

[b58] Varkonyi-Gasic E., Wu R. M., Wood M., Walton E. F. & Hellens R. P. Protocol: a highly sensitive RT-PCR method for detection and quantification of microRNAs. Plant Methods 3, 12 (2007). 17931426 10.1186/1746-4811-3-12 PMC2225395

