# Supplementary material for: A cotton miRNA is involved in regulation of plant response to salt stress
Source: Sci Rep. 2016 Jan 27;6:19736. doi: 10.1038/srep19736 (PMC4728436; doi:10.1038/srep19736)
Supplement: Supplementary Data [file srep19736-s1.pdf]

# Supplementary Data

**Title of article**: A cotton miRNA is involved in regulation of plant response to salt stress

**Running head:** miRNVL5 and *GhCHR* involved in plant salt stress response

**Full names of all authors:** [1*]Shuai Gao, [1*]Lu Yang, [5]Hou Qing Zeng, [2]Zhao Sheng Zhou, [1]Zhi Min Yang, [1,3]Hua Li, [1,6]Di Sun, [4]Fuliang Xie, [4]Baohong Zhang

**Supplementary Data S1. Sequences of mature miRNVL5 (red) and its precursor (yellow)**

Primary transcripts of miRNVL5

GATCTGGGCTTGGGTGCCTATAAATTCTTTAGGGGAAAAAGTAGGGCATCAGAGTGTATAGCCGAATATGCAAAAAGGCGAGGG
TGAGGGTGGAGTTTGAATCAAACAAGAAAATCATATTCTTACCTTCTTTTCTTCACAAATATCACAATAAAACATGGATGAAACT
ATCGATGCCCAAAGCTGCCTCTTGTATCGATGATAGAGTTCTATTATTGTGATATTAGTGAGGAACAAAAGGTATTGATATGAGG
TCGACGGCCATGACCAGATTCTGTCGGATATACACTCACCGTGTGCTGCGTACTGCTTTATTATCTTTGCCTC

Pre-miRNVL5

UUUCUUCACAAAUAUCACAAUAAAACAUGGAUGAAACUAUCGAUGCCCAAAGCUGCCUCUUGUAUCGAUGAUAGAGUUCUAU

UAUUGUGAUAUUAGUGAGGAAC

Mature miRNVL5
UUUCUUCACAAAUAUCACAAUA

## Supplementary Data S2. Sequences of *GhCHR* and deduced amino acids of GhCHR

## A: Sequences of *GhCHR* cDNA

ATGCTCTCGATCATTATATTTCTTCACTTGTAATCTTGCTTTTGGACTCTCTCTCTCTCTTTCCAATTTGTACTATACTTCAAACATTGAGTTTCAGTTTTTTTCTTCC
TACGTCAATGCAAAAAACCAAAGAGGAAGGAGAA**ATG**GAGGAGTCTAACAATTATGGCCACCAACATCCCCTGCTTCTTATCTTGAATCAAGACCAGCTGATC
CACAATCAAAGTGGTGTAACTCATTGCTCCAGGTGTGGGGAGAAGGTGTCTGCTCCATGTTTTTGCTGTGCGGAGCGCTGTGGGTTTTATGTCCACAAGGTATG
TGCCGAGGCACCTTTGGAGCTTAATCATCCTTTTCATACTCATCATCCTCTTCTTCTTATGCAAAATGCCCCTTATTCATCTGGACGGTACATTTGCAATTTTTGCG
ATAAAGACGGTAGTAAGTTTGTTTATCACTGCTCTTGCGGATTGGACTTTCATATTAAATGTGCTTTGTTTACATTTAATATTGCTGAGAATAATTTGAAAGAGCTT
GACCATGTTGCCCTTCAACATCCTTTGATTTCCACTGAAAACGGTGATGAAGAACTTGGAGACGTTAGTAAGTGCTTTGGATGTTGGGAACCATTAGCAAAGTA
TACACACTTTTCTCCTGACTGTGGATTTAATTTCCATGAGAAATGCGCTAAGCTTCCTTTCAAACTGAATCATGAGTGCCATCGCAAGCATCCTCTTGCTCTACAA
TTTAATAGCAAACGGCTCTCTTGCAAGATATGCAGAGAAACCAATCGAAAAACAGATCGCAGGCGTATAGGATTTGTTTATGGTTGTTCACCCTGTAAGTTTGCT
GTTCACATTGAATGTGTATCAGCATCTTTAGATCTTGTTGTTGAAGATAAAAGCCATGAACATCCTCTCAGTTTGTTTACAAGGAGATCATCATTCATTTGTGATG
CATGTGGTATGGAAGGAAGTTATGCTTCCTACATATGTTGTACATGCAACATTATGGTTCATAAAAAGTGCACTTCATTGCCGCGCATCATCAAAAGCAAGTGGC
ATGATCATCGCCTTTTTCACATATATTTCTTTCCGGATGAATTTACAAGTTCAGACTGCATGATTTGTCATGATGAAGTCGATCCAGAGCATGGTAGTTATTGCTGT
TCACATTGCAATATTACATTCCATGTGCGTTGTGTGACAGAGGATAAACGCTCATATTCAATAGTTTCACTAGAAAATGAAGATGAGATGCCCAATGAAAGTTCC
ATCATTGTTATTGAGAGCAACGATGCTGGAGAAGCTACAAAAATAAAGCATTTCAAGCATATGCATAATCTAATGTTAGGTCCCTTTGATGGAGGATACGAAAAT
AATTGCGACGGGTGTATGTTGCCAATCTCGGATCCATTTTACTACTGTTCAGAATGTGTTTTTTTTCTTCATAAAGCGTGTGCCGAGTTACCTAAGATGAAGAATG
TTTGGCATGAGTTGTGCCGAGAACCTCTTGCCCTTATTTCAGACAAAGTTTTTGAGTGTGCGAAATGTCGGCACATATCTAATACCTTTGCTTATGAATGTAGTG
AATGTGAGAGTAAGAGATGTCTCAGATGTGTGATTGTGCTTACCCCTGGTGCTCGAACATCTTTGAGACATGAGCACCCCCTCTTTTTCTACAAAGATTACCATG
GGCGCTGTGATGCTTGTGGTAATCTTACATTGGGAGCATTTTGTTGTAAGGATTGCAATTTTGTGCTACATTTTGGATGTTTTTCACTTCCAATTACAGCTCATCAC
AAATGTGATGAGCATCTTCTTTCACTCACTGCTCATAATGATAACAAATATTTAGAAAGTCATTATTGCGATATTTGTGAAGAAAGTCGAGATACAAATCGTTGGT
TTTATCATTGTGCAATATGCGATACTTCTGTTCATGTTAATTGTGTTCTTGGAAAATATCCATTCCTCAAACTTGGGAGCATATTTGAAGAAACAGATCATCCACAC
CCACTCACTATCGTGAAGAAGAAGTATTACTACCTTGATTGTAATAAATGCGGTAAGCCTTGTGAAGATTTATCTCTTGAGTGCTCAAAGTTGGAGTGTAAATATA
TTGTCCACTTGGATTGTGTAGTACACTACACTCTACGGTGTTTTTTGTGGTGGCGCATG**TAA**CAATTCAGATGTAATCATATTTGAAACCTATCACTTCATTTGTG
GGAAAACTAAAGAGTGGAGAACAGGGTTTGATCTTTGTCTTGTTGCTTGTGATGTTAGTGTTGTCATTGTAATTTGGTTTTTTTGACCTTTTAAGTTTTAAAGAA
ATGTCATGTAATAATCTTAAGTTGAAACTTGTGTGATTTGCATTGATGTGTGATTGTTGATTTTAGAATTTCTTGTAATATGGGAGTGTGGTTGTACCTACAACAAT
AAAGGTGTTGCTGGATGCTTCTCTTTGTTTCTTTATATAAGTTTTCTTG

**Yellow letters: cleavage cite**

**B: Deduced amino acids (677 aa) and domains of GhCHR**

MEESNNYGHQHPLLLILNQDQLIHNQSGVTHCSRCGEKVSAPCFCCAERCGFYVHKVCAEAPLELNHPFHTHHPLLLMQNAPYSSGRYICNFCDKDGSKFV
YHCSCGLDFHIKCALFTFNIAENNLKELDHVALQHPLISTENGDEELGDVSKCFGCWEPLAKYTHFSPDCGFNFHEKCAKLPFKLNHECHRKHPLALQFNSK
RLSCKICRETNRKTDRRRIGFVYGCSPCKFAVHIECVSASLDLVVEDKSHEHPLSLFTRRSSFICDACGMEGSYASYICCTCNIMVHKKCTSLPRIIKSKWHDH
RLFHIYFFPDEFTSSDCMICHDEVDPEHGSYCCSHCNITFHVRCVTEDKRSYSIVSLENEDEMPNESSIIVIESNDAGEATKIKHFKHMHNLMLGPFDGGYENN
CDGCMLPISDPFYYCSECVFFLHKACAELPKMKNVWHELCREPLALISDKVFECAKCRHISNTFAYECSECESKRCLRCVIVLTPGARTSLRHEHPLFFYKDY
HGRCDACGNLTLGAFCCKDCNFVLHFGCFSLPITAHHKCDEHLLSLTAHNDNKYLESHYCDICEESRDTNRWFYHCAICDTSVHVNCVLGKYPFLKLGSIFE
ETDHPHPLTIVKKKYYYLDCNKCGKPCEDLSLECSKLECKYIVHLDCVVHYTLRCFLWWRM

PHD-type zinc finger domain
C1 domain

**C: Comparison of amino acid sequence alignments between GhCHR and other species PHD (a), and GhCHR and other PHD-type finger proteins (b)**

**(a)**

```
PHD-1(GhCHR)    ----HCSRCGEKVSAP-------CFCCAER-------CGFYVHKVCAEAP--LELNHPFHTHHPLLLMQNAPYSSGRYI-CNFCD
PHD-2(GhCHR)    ----SCKICRETNRKTDRRRIGFVYGCS--------PCKFAVHIECVS-----ASLDLVVEDKSHEHPLSLFTRRSSFI-CDACG
PHD-3(GhCHR)    ----YCDICEES-RDTNR----WFYHCA--------ICDTSVHVNCVLGKYPFLKLGSIFEETDHPHPLTIVKKKYYYLDCNKCG
OsVIL1          CKRCSCCICHLFDDNKDP---SLWLVCSSE-TGDRDCCESSCHIECALQH----QKVGCVDLGQSIQ------LDGNYC-CAACGK
OsVIL2          CRRCSCCICFKYDDNKDP---SLWLFCSSDQPLQKDSCVFSCHLECALKD----GRTGIMQSGQCKK------LDGGYY-CTRCRK
OsVIL3          CKRCSCCICHQFDDNKDP---SLWLVCASE-NDDKNCCGSSCHIECALQH----KRVGCFNLGNIIQ------LDGSYS-CASCGK
OsVIL4          CKRCSCCICHKYDENKDP---SLWLVCSSDTPYSGYSCGTSCHLKCALKN----KKAGIFKNGCNKK------SDGSFY-CVWCGK
AtVIL1          CKRCSCCVCHNFDENKDP---SLWLVCEPEKSDDVEFCGLSCHIECAFRE----VKVGVIALGNLMK------LDGCFC-CYSCGK
AtVIL2          CRRCSCCICRKYDDNKDP---SLWLTCSSDPPFEGESCGFSCHLECAFNT----EKSGLGKDKQSE--------GCCFY-CVSCGK
AtVIL3          CKRCSCCICFKYDDNKDP---SLWLTCNSDSQFDGESCGLSCHLNCAFDS----EKSGLKEDTPSSD------IDGCFN-CVSCGK
TmVIL1          CKRCSCCICHLFDDNKDP---SLWLVCSSE-TGDTDCCESSCHVECALQR----RKAGRIDLGQSMH------LDGNYC-CAACGK
TmVIL2          CRRCSCCICFKYDDNKDP---TIWLSCSSDHPMQKDSCGLSCHLECALKD----GRTGILPSGQCKK------LDGAYY-CPNCRK
TmVIL3          CKRCSCCICHQFDDNKDP---SLWLVCASE-NDDKQCCGSSCHIECALQQ----KRVGCFDLQKIIH------LDGSYS-CASCGK
KAP1            ---TICRVCQKPGD---------LVMCN--------QCEFCFHLDCHLP--------ALQDVP-----------GEEWS-CSLCHV
WSTF            ---ARCKVCRKKGEDDK------LILCD--------ECNKAFHLFCLRP--------ALYEVP-----------DGEWQ-CPACQP
Pygopus         ---YPCGMCHKEVNDNDE-----AVECES-------GCNFFFHRTCVGLT-----EAAFQMLNKEVF--------AEWC-CDKCVS
Cys4-His-Cys3        *  *                 *          *    *  *                               :  *  *
                     C  C                 C          C    H  C                                  C  C
```

**(b)**

```
C1-1(GhCHR)       HNLMLGPFDGGYENNCDGCMLPIS---DPF------YYCSECVFFLHKACAELPKM----KNVWHELC
C1-2(GhCHR)       -HEHPLFFYKDYHGRCDACGNLT-----LGA-----FCCKDCNFVLHFGCFSLPITAH-HKC
Raf1              HNFARKTFL--KLAFCDICQKFL-----LNG-----FRCQTCGYKFHEHCSTKVPT----MC
DGK1              VRWTEGADQTDDSSFCSYCDESCSSSFLGGS---PIWCCLWCQRLVHVDCHSNMSNETGDIC
DGKD              HQWLEGNLP--VSAKCTVCDKTCGSVLRLQD-----WRCLWCKAMVHTSCKESLLT----KC
DGKQ              HHWREGNLP--SGARCEVCRKTCGSSDVLAG-----VRCEWCGVQAHSLCSAALAP----EC
DAGK5             HHWIRGNVP--LCSYCMVCKQQCGCQPKLCD-----YRCIWCQKTVHDECMKNSLKNE--KC
CIT               HRFNVGLNM--RATKCAVCLDTVHFGRQAS-------KCLECQVMCHPKCSTCLPA----TC
rdgA              HYWKPTSAS---GDLCCLCEECIK---SGQR-----MKCSACQLVAHHNCIPFVNEKSTLAC
Dgkz              HHWVHRRRQ---DGKCRHCGKGFQQKFTFHSKEIVAISCSWCKQAYHSKCSCFMLQQIEEPC
KPC1              HRFIPFSNL--TANWCCHCGYMLPIGSKKNS-----RKCSECALTAHAQCVHLVPD----FC
OSJNBb0024A20-2   -------------GWCDLCQGPIQ-PGRLV------YRCAECAFDVHPLCT
                                 *  *                   *  *    *  *
                                 C  C                   C  C    H  C
```

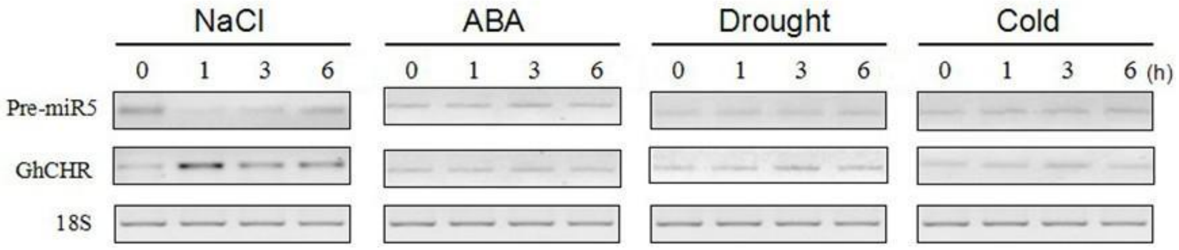


**Supplementary Data S3.** Effects of salinity, ABA, drought and cold stresses on expression of pre-miRNVL5 and *GhCHR* in seedlings of cotton (*Gossypium hirsutum*) plants. Two week-old cotton seedlings were treated with NaCl (0-400 mM) (A), ABA (100 μM), drought (20% polyethylene glycol 600) and cold (4 ℃) for 0, 1, 3 and 6 h. After that, total RNA was extracted and analyzed by semi-quantitative RT-PCR.

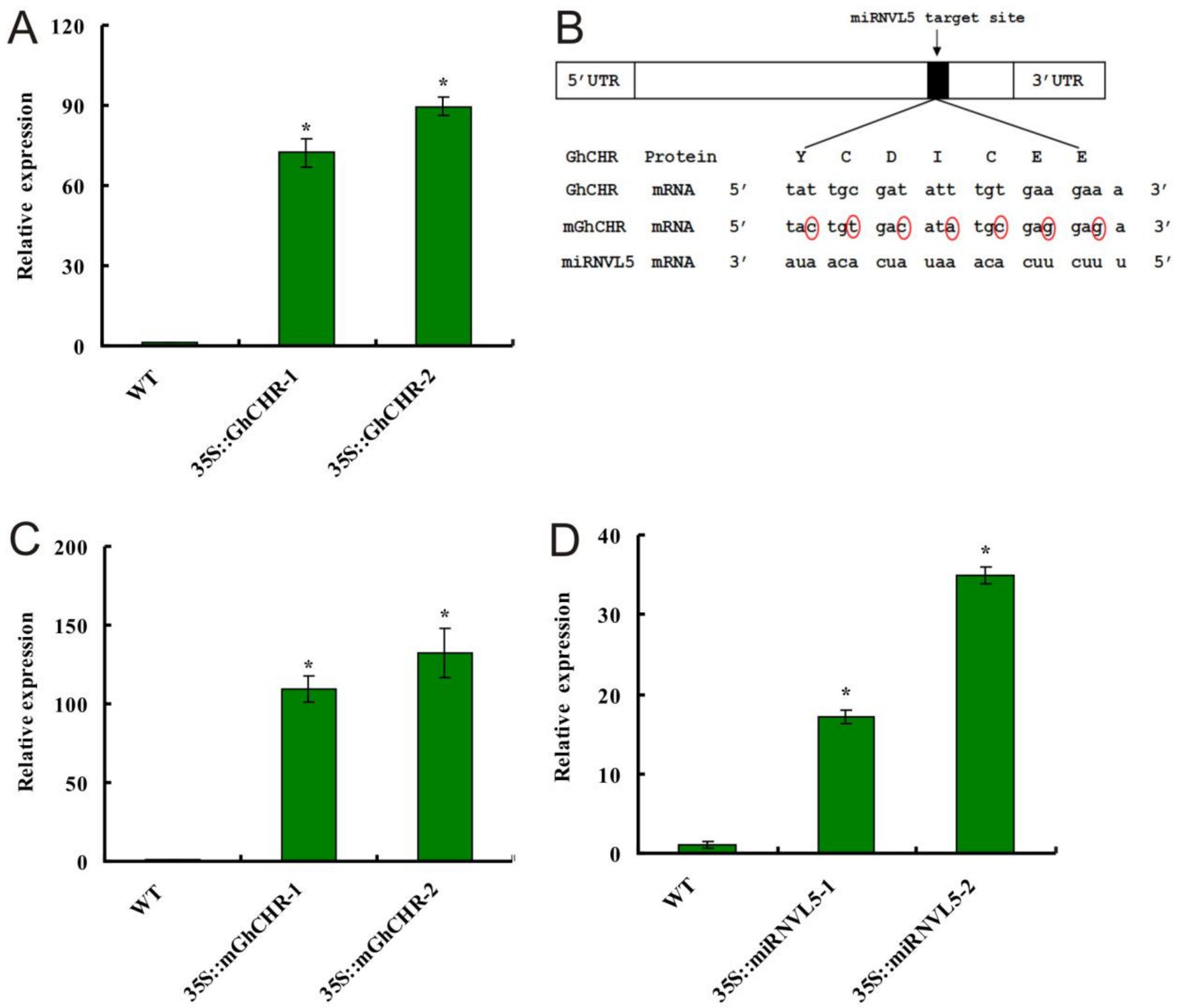


**Supplementary Data S4.** Expression of *GhCHR* and miRNVL5in transgenic Arabidopsis and wild type (WT). Transcripts in 4 week-old

seedlings were analyzed using qRT-PCR. Values are the means ± standard deviation (SD). Means followed by different letters are significantly different at $p < 0.05$. A: qRT-PCR analysis of *GhCHR* mRNA in transgenic lines overexpressing *GhCHR* (*35S::GhCHR*). B: Base paring of miRNVL5 with its corresponding complementary site of *GhCHR* and miRNVL5-resistan form of *mGhCHR*. C: qRT-PCR analysis of *GhCHR* mRNA in transgenic lines overexpressing miRNVL5-resistant form (*35::mGhCHR*). D: qRT-PCR analysis of miRNVL5 in *35S::miRNVL5* Arabidopsis lines.

## Supplementary Data S5 Differential expression of genes in WT and miRNVL5 under salt stress

| AGI ID | Description | WT+S/WT (Up) | miR5+S/WT (Up) | miR5+S/WT+S (Down) |
|---|---|---|---|---|
| AT1G02460 | Pectin lyase-like protein | 3.8511736 | 3.8511736 | 1 |
| PP2CA | protein phosphatase 2C 37 | 9.376997 | 9.376997 | 1 |
| COL1 | zinc finger protein CONSTANS-LIKE 1 | 2.462924 | 2.462924 | 1 |
| 42097 | 5'-adenylylsulfate reductase 3 | 2.8906884 | 2.8906884 | 1 |
| AT2G26610 | Transducin family protein / WD-40 repeat family protein | 12.228756 | 12.225176 | 1.0002929 |
| AT4G19200 | proline-rich family protein | 2.4270766 | 2.4259715 | 1.0004555 |
| CCA1 | protein CCA1 | 2.565155 | 2.563953 | 1.0004687 |
| AT1G75000 | GNS1/SUR4 membrane-like protein | 2.718698 | 2.7173057 | 1.0005125 |
| NAC053 | NAC domain containing protein 53 | 2.3932486 | 2.3912387 | 1.0008405 |
| AT4G26580 | RING/U-box domain-containing protein | 2.0456853 | 2.0437539 | 1.0009451 |
| ABF1 | abscisic acid-insensitive 5-like protein 4 | 3.1246445 | 3.121655 | 1.0009576 |
| AT1G29640 | hypothetical protein | 3.9133673 | 3.9087703 | 1.001176 |
| PIP2B | aquaporin PIP2-2 | 2.538842 | 2.5351675 | 1.0014493 |
| AT5G18340 | U-box domain-containing protein 48 | 2.4465628 | 2.442946 | 1.0014805 |
| AT4G01280 | myb family transcription factor | 2.525326 | 2.5215228 | 1.0015084 |
| AT2G42270 | U5 small nuclear ribonucleoprotein helicase | 2.3193634 | 2.3152368 | 1.0017824 |
| AT5G08139 | RING/U-box domain-containing protein | 2.0949938 | 2.090961 | 1.0019288 |
| AT1G24580 | RING/U-box domain-containing protein | 12.445006 | 12.416526 | 1.0022938 |
| AT1G73390 | Endosomal targeting BRO1-like domain-containing protein | 4.4853163 | 4.4744835 | 1.002421 |
| CK1 | choline kinase 1 | 2.7362354 | 2.7288456 | 1.002708 |
| EDA9 | D-3-phosphoglycerate dehydrogenase | 2.944129 | 2.9350524 | 1.0030925 |
| ENODL2 | early nodulin-like protein 2 | 3.8698719 | 3.857839 | 1.003119 |
| PPCK1 | phosphoenolpyruvate carboxylase kinase 1 | 2.0093555 | 2.0029452 | 1.0032005 |
| AT1G60750 | auxin-induced atb2-like protein | 2.4820645 | 2.473724 | 1.0033717 |
| AT4G29770 | hypothetical protein | 2.087976 | 2.0809488 | 1.003377 |
| AT4G33550 | bifunctional inhibitor/lipid-transfer protein/seed storage 2S albumin-like protein | 2.221798 | 2.21298 | 1.0039847 |

| | | | | |
|---|---|---|---|---|
| AT1G53210 | calcium-binding EF-hand domain-containing protein | 2.1517618 | 2.143158 | 1.0040146 |
| MPK19 | mitogen-activated protein kinase 19 | 3.1702876 | 3.156023 | 1.0045198 |
| AT3G04000 | 3-oxoacyl-[acyl-carrier protein] reductase | 3.0724297 | 3.0579948 | 1.0047203 |
| AT3G48990 | AMP-dependent synthetase and ligase-like protein | 2.0772448 | 2.0674515 | 1.0047369 |
| AT2G13960 | myb family transcription factor | 9.235979 | 9.191244 | 1.0048671 |
| AT4G19200 | proline-rich family protein | 2.1638145 | 2.1531277 | 1.0049634 |
| AT5G18636 | hypothetical protein | 2.7230318 | 2.7093375 | 1.0050545 |
| tic20-IV | translocon at the inner envelope membrane of chloroplasts 20-IV | 2.1836915 | 2.1717591 | 1.0054944 |
| AT5G17760 | AAA-type ATPase family protein | 2.7458513 | 2.730809 | 1.0055083 |
| AT3G09440 | protein heat shock protein 70-3 | 2.0231476 | 2.0119846 | 1.0055482 |
| NAC053 | NAC domain containing protein 53 | 2.3470216 | 2.3337862 | 1.0056711 |
| AT5G59490 | haloacid dehalogenase-like hydrolase domain-containing protein | 3.3904226 | 3.3711092 | 1.0057291 |
| ATGLR1.2 | glutamate receptor 1.2 | 2.3588674 | 2.3444586 | 1.006146 |
| UPL5 | E3 ubiquitin-protein ligase UPL5 | 2.1834302 | 2.168924 | 1.0066881 |
| AT5G02020 | hypothetical protein | 11.525537 | 11.445476 | 1.006995 |
| AT1G71110 | hypothetical protein | 2.0834332 | 2.0675335 | 1.0076902 |
| PP2CA | protein phosphatase 2C 37 | 7.3281417 | 7.2702947 | 1.0079566 |
| DHAR2 | glutathione S-transferase DHAR2 | 2.4653754 | 2.4451206 | 1.0082839 |
| AT1G30080 | glycosyl hydrolases family 17 domain-containing protein | 2.5611393 | 2.5400572 | 1.0082998 |
| AT3G59350 | protein kinase family protein | 2.2995615 | 2.2803025 | 1.0084459 |
| AT3G62260 | putative protein phosphatase 2C 49 | 4.2880135 | 4.2510805 | 1.0086879 |
| DAR6 | protein DA1-related 6 | 2.2634008 | 2.2436376 | 1.0088085 |
| AT1G02820 | late embryogenesis abundant 3-like protein | 2.1471086 | 2.1279118 | 1.0090213 |
| RD19 | cysteine proteinase RD19a | 2.0283031 | 2.0101342 | 1.0090387 |
| AT3G57540 | Remorin family protein | 7.1788316 | 7.1140885 | 1.0091007 |
| AT2G35990 | cytokinin riboside 5'-monophosphate phosphoribohydrolase LOG2 | 2.5287726 | 2.505227 | 1.0093986 |
| AT1G59860 | HSP20-like chaperone | 3.7089167 | 3.6736968 | 1.009587 |
| AT3G25870 | hypothetical protein | 2.93942 | 2.9114687 | 1.0096004 |
| VAMP725 | vesicle-associated membrane protein 725 | 2.133735 | 2.1132956 | 1.0096718 |

| | | | | |
|---|---|---|---|---|
| AT1G22985 | ethylene-responsive transcription factor ERF069 | 2.787973 | 2.7606752 | 1.009888 |
| SHM7 | serine hydroxymethyltransferase 7 | 3.0827909 | 3.0515614 | 1.010234 |
| AT4G35720 | hypothetical protein | 2.086973 | 2.0638304 | 1.0112134 |
| ATSAC1B | Phosphoinositide phosphatase-like protein | 7.908695 | 7.816103 | 1.0118463 |
| NAM | NAC domain-containing protein 18 | 2.25321 | 2.2268207 | 1.0118507 |
| ADR1-L1 | putative disease resistance protein ADR1-like 1 | 2.4015775 | 2.3734019 | 1.0118715 |
| CRK15 | cysteine-rich receptor-like protein kinase 15 | 2.1189244 | 2.0931818 | 1.0122982 |
| MSS1 | sugar transport protein 13 | 2.305659 | 2.277624 | 1.012309 |
| AT2G39350 | ABC transporter G family member 1 | 2.7211144 | 2.687994 | 1.0123217 |
| AT4G31196 | oxidoreductase | 2.030236 | 2.0051348 | 1.0125184 |
| AT2G38410 | VHS and GAT domain-containing protein | 2.4555843 | 2.4244428 | 1.0128448 |
| EDA12 | embryo sac development arrest 12 protein | 7.1828237 | 7.0903234 | 1.013046 |
| PIF7 | transcription factor PIF7 | 2.2016892 | 2.1721625 | 1.0135932 |
| BCB | blue copper protein | 2.5548491 | 2.5201647 | 1.0137627 |
| RHA2B | putative E3 ubiquitin-protein ligase RHA2B | 2.2795956 | 2.2479763 | 1.0140657 |
| RLP53 | receptor like protein 53 | 7.283559 | 7.1805067 | 1.0143516 |
| SAG13 | senescence-associated protein 13 | 2.3223643 | 2.2894979 | 1.0143553 |
| UGT76E11 | UDP-glucosyl transferase 76E11 | 5.1533446 | 5.0795536 | 1.014527 |
| AT1G10585 | basic helix-loop-helix domain-containing protein | 3.3822453 | 3.3334074 | 1.0146511 |
| AT3G19240 | Vacuolar import/degradation, Vid27-related protein | 3.3131528 | 3.2649066 | 1.0147772 |
| HAT22 | homeobox-leucine zipper protein HAT22 | 2.389207 | 2.354345 | 1.0148073 |
| AT2G38340 | dehydration-responsive element-binding protein 2E | 3.9656396 | 3.9065282 | 1.0151315 |
| UGT73B5 | UDP-glucosyl transferase 73B5 | 2.1340735 | 2.1016448 | 1.0154301 |
| CK1 | choline kinase 1 | 2.6714456 | 2.630302 | 1.0156422 |
| AT4G38060 | hypothetical protein | 2.4016402 | 2.3627048 | 1.0164791 |
| ZF2 | zinc-finger protein 2 | 2.64291 | 2.5994618 | 1.0167143 |
| AT3G24420 | hydrolase, alpha/beta fold family protein | 2.436751 | 2.396174 | 1.0169342 |
| PUB18 | U-box domain-containing protein 18 | 2.3034623 | 2.2643697 | 1.0172642 |
| AT1G27200 | hypothetical protein | 2.1434767 | 2.1067092 | 1.0174525 |

| | | | | |
|---|---|---|---|---|
| AT2G32050 | hypothetical protein | 2.2732198 | 2.2334871 | 1.0177895 |
| AT1G51440 | phospholipase A1-Igamma3 | 3.06474 | 3.01049 | 1.0180203 |
| NF-YC3 | nuclear transcription factor Y subunit C-3 | 2.1136992 | 2.075388 | 1.0184599 |
| AT3G11420 | hypothetical protein | 4.1061177 | 4.0280066 | 1.019392 |
| ATAF1 | NAC domain-containing protein 2 | 7.803936 | 7.6455226 | 1.0207198 |
| AFP3 | Ninja-family protein AFP3 | 7.935931 | 7.774674 | 1.0207413 |
| EDL3 | EID1-like F-box protein 3 | 19.152058 | 18.751087 | 1.0213839 |
| AT1G07400 | class I heat shock protein | 4.0619917 | 3.9766335 | 1.021465 |
| WIN2 | putative protein phosphatase 2C 59 | 2.1175857 | 2.072628 | 1.0216911 |
| AT4G11570 | haloacid dehalogenase-like hydrolase domain-containing protein | 2.1456683 | 2.0999494 | 1.0217716 |
| EXLA3 | expansin-like A3 | 2.2884552 | 2.2396479 | 1.0217924 |
| AT1G54050 | HSP20-like chaperone | 3.875778 | 3.7923393 | 1.0220019 |
| AT4G10510 | Subtilase family protein | 2.2687035 | 2.217894 | 1.0229089 |
| RAP2.10 | ethylene-responsive transcription factor RAP2-10 | 3.0793612 | 3.0101774 | 1.0229833 |
| AT5G05220 | hypothetical protein | 4.3875866 | 4.2886567 | 1.0230678 |
| AT1G01500 | Erythronate-4-phosphate dehydrogenase-like protein | 2.3210428 | 2.2678173 | 1.0234699 |
| AT1G07500 | hypothetical protein | 3.0693867 | 2.9987595 | 1.0235522 |
| UGT73B2 | UDP-glucosyltransferase 73B2 | 4.6087947 | 4.5008173 | 1.0239906 |
| AT1G47980 | hypothetical protein | 2.8899672 | 2.8211362 | 1.0243982 |
| HSP18.2 | heat shock protein 18.2 | 4.087278 | 3.9885936 | 1.0247416 |
| ATMS1 | 5-methyltetrahydropteroyltriglutamate--homocysteine methyltransferase | 2.1730587 | 2.1205006 | 1.0247856 |
| AT4G27560 | UDP-glycosyltransferase-like protein | 2.9700515 | 2.8962984 | 1.0254647 |
| MYB96 | myb domain protein 96 | 3.9733384 | 3.872034 | 1.0261631 |
| AT3G28600 | AAA-type ATPase family protein | 9.98129 | 9.7150345 | 1.0274066 |
| ARSK1 | root-specific kinase 1 | 2.7108495 | 2.638081 | 1.0275838 |
| AT4G20320 | putative CTP synthase | 5.2255983 | 5.0851007 | 1.0276294 |
| AT2G44970 | alpha/beta-hydrolase domain-containing protein | 2.2804449 | 2.2186573 | 1.0278491 |
| RLP7 | receptor like protein 7 | 2.444479 | 2.378092 | 1.0279161 |
| AT4G20880 | ethylene-responsive/regulated nuclear protein | 2.1235487 | 2.0651104 | 1.028298 |

| | | | | |
|---|---|---|---|---|
| AT1G17745 | D-3-phosphoglycerate dehydrogenase | 2.1295612 | 2.0708272 | 1.0283625 |
| TIP2;2 | putative aquaporin TIP2-2 | 3.8160532 | 3.7107217 | 1.0283856 |
| AT5G59760 | hypothetical protein | 4.4959984 | 4.3718934 | 1.0283871 |
| AT1G52560 | HSP20-like chaperone | 3.9490964 | 3.8399632 | 1.0284203 |
| AT5G52660 | myb family transcription factor | 3.3128285 | 3.2210765 | 1.028485 |
| LEA | dehydrin LEA | 5.4011555 | 5.251152 | 1.0285659 |
| AT3G11580 | AP2/B3 domain-containing protein | 2.4127853 | 2.3455932 | 1.0286461 |
| AT2G17500 | auxin efflux carrier-like protein | 3.1851935 | 3.0961902 | 1.028746 |
| MYB34 | myb domain protein 34 | 2.3322704 | 2.2660527 | 1.0292215 |
| UBQ10 | polyubiquitin 10 | 2.786512 | 2.706367 | 1.0296135 |
| PGP11 | P-glycoprotein 11 | 3.0322697 | 2.9405646 | 1.0311862 |
| ACHT4 | thioredoxin-like 1-1 | 2.567963 | 2.490058 | 1.0312864 |
| AP4.3A | putative protein kinase | 2.085008 | 2.0217278 | 1.0313001 |
| ZF14 | MATE efflux family protein | 3.392985 | 3.289575 | 1.0314357 |
| NAC004 | NAC domain-containing protein 4 | 3.138707 | 3.0428414 | 1.0315052 |
| AT4G30730 | hypothetical protein | 3.4569237 | 3.3512847 | 1.0315219 |
| AT2G02870 | F-box/kelch-repeat protein SKIP11 | 2.485128 | 2.408779 | 1.0316962 |
| AT5G02950 | PWWP domain-containing protein | 2.3923345 | 2.3181143 | 1.0320175 |
| ERD14 | dehydrin ERD14 | 3.0535753 | 2.9585083 | 1.0321335 |
| LEA4-5 | late embryogenesis abundant protein 4-5 | 70.16928 | 67.976746 | 1.0322542 |
| AT3G52310 | ABC transporter G family member 27 | 4.669054 | 4.5207534 | 1.0328044 |
| AT3G24090 | putative glucosamine-fructose-6-phosphate aminotransferase | 2.6950414 | 2.6086745 | 1.0331075 |
| MTHSC70-2 | mitochondrial HSO70 2 | 2.273379 | 2.199183 | 1.033738 |
| RXW8 | lipase/hydrolase | 4.537028 | 4.3880157 | 1.0339588 |
| 42095 | 5'-adenylylsulfate reductase 1 | 2.3134131 | 2.2371266 | 1.0341003 |
| AT3G54410 | hypothetical protein | 3.028638 | 2.9286673 | 1.0341351 |
| AT1G69360 | hypothetical protein | 2.4258575 | 2.3456955 | 1.0341741 |
| DAD1 | putative phospholipase A1 | 40.62535 | 39.259502 | 1.0347903 |
| AT2G45820 | Remorin | 2.2337618 | 2.1562598 | 1.0359428 |

| | | | | |
|---|---|---|---|---|
| SHM7 | serine hydroxymethyltransferase 7 | 2.9380777 | 2.8361108 | 1.035953 |
| AT5G15660 | putative F-box protein | 31.801104 | 30.683235 | 1.0364325 |
| DREB1A | dehydration-responsive element-binding protein 1A | 20.748535 | 20.01548 | 1.0366244 |
| AT3G61390 | U-box domain-containing protein 36 | 2.822859 | 2.7223542 | 1.0369184 |
| ERD14 | dehydrin ERD14 | 2.9464467 | 2.8405256 | 1.0372893 |
| AZG1 | adenine/guanine permease AZG1 | 2.1107266 | 2.0344384 | 1.0374985 |
| AT1G51820 | putative LRR receptor-like serine/threonine-protein kinase | 2.153038 | 2.0751793 | 1.037519 |
| ERD10 | dehydrin ERD10 | 7.434529 | 7.161176 | 1.0381714 |
| TPD1 | tapetum determinant 1 | 2.1945825 | 2.11272 | 1.0387474 |
| AT4G22758 | hypothetical protein | 3.7654164 | 3.6248004 | 1.0387927 |
| RAP2.4 | ethylene-responsive transcription factor RAP2-4 | 2.290185 | 2.2045991 | 1.0388216 |
| ZFHD1 | zinc finger homeodomain 1 | 5.742815 | 5.527686 | 1.0389184 |
| PDE149 | TerC integral membrane domain-containing protein | 2.206762 | 2.1238978 | 1.0390152 |
| AT1G76600 | hypothetical protein | 2.5434597 | 2.4468005 | 1.0395044 |
| AT4G39130 | Dehydrin family protein | 2.8365617 | 2.7270586 | 1.0401542 |
| ERD14 | dehydrin ERD14 | 2.794719 | 2.6866918 | 1.0402082 |
| NAC019 | NAC domain-containing protein 19 | 18.601969 | 17.882786 | 1.0402164 |
| AT4G38060 | hypothetical protein | 3.2814481 | 3.1544776 | 1.0402509 |
| TRANS11 | phospho-N-acetylmuramoyl-pentapeptide-transferase-like protein | 2.9707701 | 2.8545206 | 1.0407248 |
| AT2G25625 | hypothetical protein | 3.4894059 | 3.3510377 | 1.0412911 |
| SVL4 | protein SEUSS-like 4 | 9.069902 | 8.709818 | 1.0413424 |
| AT3G54390 | sequence-specific DNA binding transcription factor | 2.3508694 | 2.2574918 | 1.0413634 |
| AT5G42570 | B-cell receptor-associated 31-like protein | 2.5386002 | 2.4376693 | 1.0414047 |
| DOF1 | Dof zinc finger protein DOF1.7 | 2.5306518 | 2.4293928 | 1.0416808 |
| AT1G66510 | A1 cistron-splicing factor AAR2 | 2.4360616 | 2.3384948 | 1.041722 |
| AT2G25625 | hypothetical protein | 3.126975 | 3.0007167 | 1.0420761 |
| AT4G12430 | haloacid dehalogenase-like hydrolase domain-containing protein | 3.6481054 | 3.498471 | 1.0427713 |
| AT3G17770 | Dihydroxyacetone kinase | 2.557847 | 2.451113 | 1.0435451 |
| AT2G39050 | hydroxyproline-rich glycoprotein-like protein | 2.6351573 | 2.524984 | 1.0436333 |

| | | | | |
|---|---|---|---|---|
| MYB7 | myb domain protein 7 | 2.6653318 | 2.5530968 | 1.0439605 |
| NAC096 | NAC domain containing protein 96 | 2.2527797 | 2.157145 | 1.0443338 |
| GID1B | putative gibberellin receptor GID1L2 | 2.5090659 | 2.402426 | 1.0443884 |
| AT1G03070 | transmembrane BAX inhibitor motif-containing protein | 4.3146095 | 4.1302834 | 1.044628 |
| FLS6 | flavonol synthase 6 | 9.640937 | 9.228193 | 1.0447264 |
| AT1G30740 | FAD-binding and BBE domain-containing protein | 2.2573636 | 2.1606066 | 1.0447823 |
| AGO2 | Argonaute family protein | 2.8815155 | 2.7564082 | 1.0453879 |
| AT5G51845 | putative defensin-like protein 270 | 4.399197 | 4.206629 | 1.0457773 |
| AT2G43500 | RWP-RK domain-containing protein | 2.5931306 | 2.4795654 | 1.0458004 |
| AT4G27560 | UDP-glycosyltransferase-like protein | 2.5244465 | 2.4135478 | 1.0459484 |
| CER1 | CER1 protein | 11.520266 | 11.011364 | 1.046216 |
| MYB49 | myb domain protein 49 | 4.884303 | 4.6664867 | 1.0466768 |
| SRC2 | hypothetical protein | 2.7642708 | 2.6404443 | 1.0468961 |
| AT1G05870 | hypothetical protein | 2.7281206 | 2.6048303 | 1.0473315 |
| AT2G11891 | hypothetical protein | 2.1433945 | 2.0463588 | 1.0474187 |
| SQE3 | squalene epoxidase 3 | 2.4253316 | 2.3154004 | 1.0474782 |
| AER | 2-alkenal reductase | 3.6252341 | 3.4606345 | 1.0475634 |
| UGT71B1 | UDP-glucosyl transferase 71B1 | 2.3226805 | 2.2160804 | 1.048103 |
| SULTR1;2 | sulfate transporter 1.2 | 2.5374677 | 2.4203074 | 1.0484072 |
| HSC70-1 | heat shock 70kDa protein 1/8 | 2.6663823 | 2.542868 | 1.0485729 |
| AT1G04770 | male sterility MS5 family protein | 2.356081 | 2.246075 | 1.048977 |
| IDD16 | ribonuclease P subunit RPR2 | 2.1817899 | 2.0798197 | 1.0490284 |
| MSL4 | mechanosensitive channel of small conductance-like 4 | 5.788264 | 5.5171256 | 1.0491449 |
| AT1G64890 | integral membrane transporter family protein | 2.601712 | 2.4792342 | 1.0494015 |
| CYP707A4 | abscisic acid 8'-hydroxylase 4 | 2.132547 | 2.0312784 | 1.0498545 |
| RPS6 | TIR-NBS-LRR class disease resistance protein | 2.1023562 | 2.0016375 | 1.0503182 |
| HB6 | homeobox-leucine zipper protein ATHB-6 | 2.7082658 | 2.578129 | 1.0504773 |
| MPK7 | mitogen-activated protein kinase 7 | 2.1140723 | 2.0122347 | 1.0506092 |
| AT5G62570 | calmodulin binding protein-like protein | 2.5676963 | 2.4438956 | 1.0506572 |

| | | | | |
|---|---|---|---|---|
| PIP1B | aquaporin PIP1-2 | 2.3180606 | 2.2059562 | 1.0508189 |
| AT1G52565 | hypothetical protein | 2.3055844 | 2.19376 | 1.0509739 |
| CSLG2 | cellulose synthase-like protein G2 | 3.0838733 | 2.9338973 | 1.0511184 |
| AT5G64170 | dentin sialophosphoprotein-like protein | 2.2130706 | 2.1051278 | 1.0512761 |
| AT2G18340 | late embryogenesis abundant domain-containing protein | 2.1147006 | 2.0111508 | 1.0514878 |
| AT1G76980 | hypothetical protein | 2.8123887 | 2.6741426 | 1.0516974 |
| ZIF1 | zinc induced facilitator 1 proetin | 3.110975 | 2.9576938 | 1.0518246 |
| AT3G57380 | Glycosyltransferase family 61 protein | 2.409892 | 2.2910678 | 1.0518641 |
| MAPKKK18 | mitogen-activated protein kinase kinase kinase 18 | 25.252958 | 24.005972 | 1.0519449 |
| AT1G78070 | transducin/WD-40 repeat-containing protein | 10.444754 | 9.928743 | 1.0519713 |
| AT2G21640 | hypothetical protein | 2.6731732 | 2.5386388 | 1.0529947 |
| AT1G23910 | Polyketide cyclase/dehydrase and lipid transport superfamily protein | 2.27979 | 2.163984 | 1.0535152 |
| UGT73B1 | UDP-glucosyl transferase 73B1 | 2.1177523 | 2.010077 | 1.0535678 |
| APX2 | L-ascorbate peroxidase | 3.634633 | 3.446116 | 1.0547042 |
| AT4G36010 | pathogenesis-related thaumatin family protein | 5.419246 | 5.1367607 | 1.0549929 |
| AT4G38140 | RING-H2 finger protein ATL18 | 6.913354 | 6.550641 | 1.0553706 |
| PIP1A | aquaporin PIP1-1 | 2.2187233 | 2.1022868 | 1.0553856 |
| AT1G25422 | hypothetical protein | 2.987772 | 2.8304152 | 1.0555949 |
| CLPB3 | casein lytic proteinase B3 | 2.1982732 | 2.082 | 1.0558468 |
| KNAT4 | homeobox protein knotted-1-like 4 | 2.9940197 | 2.8347874 | 1.0561708 |
| AT3G17800 | hypothetical protein | 4.501618 | 4.25926 | 1.0569013 |
| SYN1 | Sister chromatid cohesion 1 protein 1 | 3.9127076 | 3.7018232 | 1.0569677 |
| DIN2 | beta-glucosidase 30 | 6.061409 | 5.7337604 | 1.0571438 |
| AT2G18150 | peroxidase 15 | 2.7225018 | 2.574692 | 1.0574087 |
| AT1G80470 | F-box/RNI-like/FBD-like domain-containing protein | 2.8756359 | 2.7182093 | 1.0579156 |
| PAP9 | putative inactive purple acid phosphatase 9 | 3.4527707 | 3.2636433 | 1.0579498 |
| CLPB4 | casein lytic proteinase B4 | 2.6313922 | 2.4866662 | 1.0582008 |
| RPS2 | disease resistance protein RPS2 | 3.2098176 | 3.0331578 | 1.0582429 |
| AT2G34930 | disease resistance-like protein/LRR domain-containing protein | 2.700568 | 2.5516407 | 1.0583653 |

| | | | | |
|---|---|---|---|---|
| AT3G15534 | hypothetical protein | 4.965611 | 4.6887016 | 1.0590588 |
| MYB7 | myb domain protein 7 | 2.4312901 | 2.2953107 | 1.0592422 |
| ECT8 | hypothetical protein | 4.0277934 | 3.8013422 | 1.0595714 |
| AT5G45350 | proline-rich family protein | 2.5937426 | 2.4461017 | 1.0603576 |
| TIP2;2 | putative aquaporin TIP2-2 | 3.3208447 | 3.131174 | 1.0605749 |
| AT2G30766 | hypothetical protein | 3.0792818 | 2.902798 | 1.0607978 |
| AT3G59940 | F-box/kelch-repeat protein SKIP20 | 2.2889652 | 2.1575902 | 1.0608897 |
| AT5G05220 | hypothetical protein | 4.0794907 | 3.844507 | 1.0611218 |
| AT3G57010 | strictosidine synthase family protein | 2.7464938 | 2.5874424 | 1.0614705 |
| MEE23 | FAD-binding and BBE domain-containing protein | 2.1637416 | 2.0374355 | 1.0619928 |
| AT1G67360 | Rubber elongation factor protein (REF) | 4.6976347 | 4.423023 | 1.0620868 |
| UBQ3 | polyubiquitin 3 | 2.264334 | 2.1313214 | 1.0624084 |
| AT1G22510 | C3HC4-type RING finger protein | 2.1807818 | 2.0501864 | 1.0636992 |
| AT1G73920 | alpha/beta-hydrolase domain-containing protein | 2.3202865 | 2.1811295 | 1.0638006 |
| RD21 | cysteine proteinase RD21a | 2.8973975 | 2.7222426 | 1.0643421 |
| AOC2 | allene oxide cyclase 2 | 3.2929387 | 3.093674 | 1.0644104 |
| AT4G37900 | hypothetical protein | 2.3272543 | 2.1861815 | 1.0645293 |
| AT2G33760 | pentatricopeptide repeat-containing protein | 5.4422894 | 5.110927 | 1.0648341 |
| EYE | golgi complex component-related protein | 2.134752 | 2.0045729 | 1.064941 |
| AT1G07590 | pentatricopeptide repeat-containing protein | 3.039896 | 2.8531473 | 1.0654536 |
| GSTU5 | glutathione S-transferase 103-1A | 2.619459 | 2.4583452 | 1.0655375 |
| AT5G19875 | hypothetical protein | 4.5227294 | 4.243556 | 1.0657876 |
| LOX3 | lipoxygenase 3 | 3.768945 | 3.5356529 | 1.0659827 |
| AT1G48100 | polygalacturonase | 13.797035 | 12.939905 | 1.0662392 |
| AT1G29770 | haloacid dehalogenase-like hydrolase | 7.267861 | 6.8153076 | 1.0664024 |
| NAC13 | NAC domain protein 13 | 3.9953833 | 3.7463064 | 1.066486 |
| ABF3 | abscisic acid-insensitive 5-like protein 6 | 10.514151 | 9.854732 | 1.066914 |
| AT2G45820 | Remorin | 2.2073405 | 2.0685737 | 1.0670832 |
| TPS11 | putative alpha,alpha-trehalose-phosphate synthase [UDP-forming] 11 | 4.985386 | 4.6685877 | 1.0678574 |

| | | | | |
|---|---|---|---|---|
| ATHSP22.0 | heat shock protein 22 | 3.4126785 | 3.192949 | 1.0688171 |
| ATAF1 | NAC domain-containing protein 2 | 6.087477 | 5.69482 | 1.0689499 |
| AT5G15290 | UPF0497 membrane protein | 7.7149644 | 7.2065654 | 1.0705466 |
| AT3G55880 | Alpha/beta hydrolase related protein | 2.7766178 | 2.591679 | 1.0713587 |
| HB-12 | homeobox-leucine zipper protein ATHB-12 | 13.968727 | 13.026286 | 1.0723492 |
| CPuORF12 | uncharacerized protein | 2.8700905 | 2.675215 | 1.0728447 |
| AT2G38010 | neutral ceramidase | 2.2595103 | 2.1058626 | 1.0729618 |
| LOX3 | lipoxygenase 3 | 3.8746355 | 3.6052625 | 1.0747166 |
| BAM5 | beta-amylase 5 | 2.6059222 | 2.4236841 | 1.0751905 |
| AT5G39520 | hypothetical protein | 5.4412827 | 5.060069 | 1.0753376 |
| AT1G52565 | hypothetical protein | 2.226828 | 2.0704837 | 1.0755111 |
| AT3G10420 | P-loop containing nucleoside triphosphate hydrolase family protein | 2.4418359 | 2.269671 | 1.0758545 |
| UGT84A3 | UDP-glycosyltransferase-like protein | 3.7606702 | 3.494789 | 1.0760794 |
| PIP1A | aquaporin PIP1-1 | 3.027691 | 2.8127542 | 1.0764151 |
| bZIP48 | basic leucine-zipper 48 | 3.1331875 | 2.9102142 | 1.0766176 |
| AT1G22190 | ethylene-responsive transcription factor ERF058 | 2.1818144 | 2.0263586 | 1.0767169 |
| GCN2 | ABC transporter F family member 2 | 3.823038 | 3.5499985 | 1.0769126 |
| KIN2 | stress-induced protein KIN2 | 3.9331732 | 3.6516497 | 1.0770949 |
| AT1G66060 | hypothetical protein | 2.6194832 | 2.431941 | 1.0771163 |
| AT4G15248 | B-box type zinc finger-containing protein | 3.619552 | 3.3600433 | 1.0772337 |
| AT2G23120 | Late embryogenesis abundant protein, group 6 | 4.2996163 | 3.9904923 | 1.0774652 |
| AT4G18920 | hypothetical protein | 2.256587 | 2.093631 | 1.0778341 |
| AT4G32208 | heat shock protein 70 (Hsp 70) family protein | 2.8377366 | 2.6323247 | 1.0780344 |
| AT1G04445 | C2H2-like zinc finger protein | 9.641439 | 8.940872 | 1.0783554 |
| SULTR3;1 | sulfate transporter 3.1 | 2.3150775 | 2.146839 | 1.0783658 |
| CSLB06 | cellulose synthase-like protein B6 | 5.8481655 | 5.420438 | 1.0789102 |
| AT3G60920 | hypothetical protein | 3.8684766 | 3.5839863 | 1.0793781 |
| ERD14 | dehydrin ERD14 | 2.8049111 | 2.5981648 | 1.079574 |
| AT3G55880 | Alpha/beta hydrolase related protein | 3.1726565 | 2.9385495 | 1.0796676 |

| | | | | |
|---|---|---|---|---|
| AT1G60190 | U-box domain-containing protein 19 | 96.19778 | 89.055695 | 1.0801979 |
| AT1G69610 | hypothetical protein | 3.7890317 | 3.507356 | 1.0803101 |
| AT1G54590 | splicing factor Prp18 domain-containing protein | 7.586597 | 7.022058 | 1.080395 |
| AT5G35380 | Protein kinase protein with adenine nucleotide alpha hydrolases-like domain | 2.8038387 | 2.5948436 | 1.0805426 |
| AT2G21780 | hypothetical protein | 2.193757 | 2.0293975 | 1.0809894 |
| MBF1C | multiprotein-bridging factor 1c | 2.6308706 | 2.4329991 | 1.0813282 |
| PLA IIIA | patatin-like protein 6 | 2.6280684 | 2.430138 | 1.0814482 |
| AT3G15300 | VQ motif-containing protein | 2.2456489 | 2.0757844 | 1.0818315 |
| AT1G12890 | ethylene-responsive transcription factor ERF088 | 2.5051978 | 2.315633 | 1.0818629 |
| COR27 | cold regulated protein 27 | 3.7497182 | 3.4657764 | 1.0819273 |
| AT2G42150 | DNA-binding bromodomain-containing protein | 5.098594 | 4.7124705 | 1.0819366 |
| AT5G02020 | hypothetical protein | 13.475785 | 12.449713 | 1.0824174 |
| AT4G18450 | ethylene-responsive transcription factor ERF091 | 2.218155 | 2.0480103 | 1.0830779 |
| AT3G62590 | lipase class 3 family protein | 2.9065497 | 2.682126 | 1.0836738 |
| SULTR1;2 | sulfate transporter 1.2 | 3.5805254 | 3.303942 | 1.0837132 |
| LTI30 | dehydrin Xero 2 | 6.7289324 | 6.208312 | 1.0838586 |
| HB-7 | homeobox-leucine zipper protein ATHB-7 | 10.941657 | 10.094676 | 1.0839038 |
| AT3G27809 | hypothetical protein | 3.775923 | 3.4822826 | 1.0843241 |
| PIP2;5 | putative aquaporin PIP2-5 | 2.4176579 | 2.228294 | 1.0849816 |
| KCS7 | 3-ketoacyl-CoA synthase 7 | 8.498386 | 7.83232 | 1.0850407 |
| AT5G64820 | hypothetical protein | 3.3860774 | 3.1200974 | 1.0852473 |
| AT5G61820 | hypothetical protein | 4.684759 | 4.315303 | 1.0856154 |
| HVA22D | HVA22-like protein d | 9.4661875 | 8.717894 | 1.0858343 |
| AT3G11340 | UDP-glycosyltransferase-like protein | 2.2768667 | 2.0968046 | 1.0858746 |
| ABF2 | abscisic acid-insensitive 5-like protein 5 | 3.0393274 | 2.7985919 | 1.0860202 |
| CPuORF17 | hypothetical protein | 2.8224933 | 2.5987694 | 1.0860884 |
| AT3G04000 | 3-oxoacyl-[acyl-carrier protein] reductase | 2.369836 | 2.1819413 | 1.0861136 |
| BGLU31 | beta glucosidase 31 | 2.633631 | 2.4239068 | 1.0865233 |
| GAMMA-TIP | aquaporin TIP1-1 | 2.3673809 | 2.178098 | 1.0869029 |

| | | | | |
|---|---|---|---|---|
| TIL | outer membrane lipoprotein Blc | 2.2303681 | 2.051126 | 1.0873872 |
| AT4G09760 | protein kinase family protein | 2.3508086 | 2.161883 | 1.0873895 |
| AT4G15420 | ubiquitin fusion degradation UFD1 family protein | 2.408962 | 2.214969 | 1.0875828 |
| AT1G67930 | Golgi transport complex-related protein | 2.2906756 | 2.1057236 | 1.087833 |
| ELIP1 | chlorophyll A-B binding, early light-inducible protein | 3.1024942 | 2.8519838 | 1.0878373 |
| HSC70-1 | heat shock 70kDa protein 1/8 | 2.6500301 | 2.4343956 | 1.0885782 |
| AT5G10695 | hypothetical protein | 3.486533 | 3.2022347 | 1.0887812 |
| AT2G47485 | hypothetical protein | 2.770049 | 2.5441642 | 1.0887855 |
| AT5G04250 | OTU-like cysteine protease family protein | 3.8621643 | 3.5465868 | 1.0889806 |
| LEA14 | putative desiccation-related protein LEA14 | 4.545418 | 4.1724725 | 1.0893823 |
| HSFA1E | heat stress transcription factor A-1e | 2.6318514 | 2.414831 | 1.08987 |
| AT4G09500 | glycosyltransferase family protein | 3.407868 | 3.1263695 | 1.09004 |
| GGPS2 | geranylgeranyl pyrophosphate synthase 4 | 2.385812 | 2.1885247 | 1.0901463 |
| AT4G12000 | SNARE associated Golgi family protein | 7.142668 | 6.551284 | 1.0902699 |
| AT4G27530 | hypothetical protein | 3.67228 | 3.365454 | 1.0911694 |
| NSP5 | nitrile specifier protein 5 | 3.9678423 | 3.6357746 | 1.0913334 |
| AT1G62580 | flavin-containing monooxygenase-like protein | 2.4706762 | 2.2629018 | 1.0918176 |
| AT5G37540 | aspartyl protease family protein | 2.2797387 | 2.0873702 | 1.0921583 |
| AT5G54165 | hypothetical protein | 3.9274912 | 3.5951982 | 1.0924269 |
| FER3 | ferritin 3 | 2.383022 | 2.1813817 | 1.092437 |
| AT4G03360 | Ubiquitin family protein | 15.917436 | 14.568823 | 1.0925685 |
| AT4G05010 | F-box protein | 2.289121 | 2.094541 | 1.0928986 |
| EGY3 | ethylene-dependent gravitropism-deficient and yellow-green-like 3 protein | 3.9212704 | 3.587466 | 1.0930474 |
| PIA1 | putative protein phosphatase 2C 20 | 33.434525 | 30.567135 | 1.0938064 |
| RD19 | cysteine proteinase RD19a | 3.275001 | 2.993046 | 1.0942034 |
| ddf2 | dehydration-responsive element-binding protein 1E | 10.690187 | 9.765122 | 1.0947316 |
| WRKY67 | putative WRKY transcription factor 67 | 4.573186 | 4.175745 | 1.0951785 |
| AT3G21890 | B-box type zinc finger-containing protein | 11.611959 | 10.602055 | 1.0952556 |
| CBF2 | dehydration-responsive element-binding protein 1C | 2.3685188 | 2.1622603 | 1.0953902 |

| | | | | |
|---|---|---|---|---|
| RCAR1 | abscisic acid receptor PYL9 | 2.6494012 | 2.4186425 | 1.0954083 |
| HAB1 | protein phosphatase 2C 16 | 5.294006 | 4.8278966 | 1.0965451 |
| AT2G03230 | GCK domain-containing protein | 2.4653292 | 2.2480142 | 1.0966698 |
| AT4G21570 | hypothetical protein | 2.89608 | 2.640715 | 1.096703 |
| AT1G11210 | hypothetical protein | 9.156326 | 8.34798 | 1.0968314 |
| ABI1 | protein phosphatase 2C 56 | 4.680163 | 4.2661414 | 1.0970482 |
| AT3G16940 | calmodulin-binding transcription activator | 2.5954406 | 2.3657775 | 1.0970773 |
| AT1G48060 | F-box protein | 2.643996 | 2.4080546 | 1.0979801 |
| AT1G19400 | Erythronate-4-phosphate dehydrogenase family protein | 2.2089067 | 2.0115206 | 1.0981278 |
| AT5G41460 | hypothetical protein | 2.3711069 | 2.1587813 | 1.0983543 |
| AT5G09530 | hydroxyproline-rich glycoprotein family protein | 2.254214 | 2.0509565 | 1.0991039 |
| BTI2 | reticulon-like protein B2 | 2.5167036 | 2.287466 | 1.1002146 |
| GA2OX6 | gibberellin 2-oxidase 6 | 2.986143 | 2.7135289 | 1.1004648 |
| SYP124 | syntaxin-124 | 8.979209 | 8.152331 | 1.1014284 |
| AT5G04760 | duplicated SANT DNA-binding domain-containing protein | 2.491902 | 2.2623425 | 1.1014699 |
| CPUORF47 | conserved peptide upstream open reading frame 47 | 3.4561515 | 3.1360335 | 1.1020772 |
| AT1G51980 | putative mitochondrial-processing peptidase subunit alpha-1 | 2.283489 | 2.070517 | 1.1028593 |
| AT5G42570 | B-cell receptor-associated 31-like protein | 2.5675826 | 2.3276696 | 1.10307 |
| AT5G58770 | dehydrodolichyl diphosphate synthase 2 | 4.6569552 | 4.220825 | 1.1033281 |
| AT4G30470 | Rossmann-fold NAD(P)-binding domain-containing protein | 2.3506145 | 2.1293979 | 1.103887 |
| AT5G24870 | RING/U-box domain-containing protein | 2.70553 | 2.4488199 | 1.1048301 |
| AT2G38905 | Low temperature and salt responsive protein | 2.7985535 | 2.5326588 | 1.1049864 |
| AT1G07500 | hypothetical protein | 3.4931538 | 3.1610363 | 1.1050661 |
| AT1G30320 | Remorin family protein | 2.9679046 | 2.68571 | 1.1050726 |
| AT1G60590 | Pectin lyase-like protein | 3.0379233 | 2.747705 | 1.105622 |
| AT1G73390 | Endosomal targeting BRO1-like domain-containing protein | 8.379757 | 7.579133 | 1.1056353 |
| AT1G71000 | chaperone DnaJ-domain containing protein | 10.471182 | 9.4704 | 1.1056747 |
| AGL91 | protein agamous-like 91 | 4.339707 | 3.924932 | 1.1056769 |
| AT3G27600 | SWAP (Suppressor-of-White-APricot)/surp RNA-binding domain-containing | 3.905098 | 3.531612 | 1.1057551 |

| | | | | |
|---|---|---|---|---|
| | protein | | | |
| AT5G60380 | hypothetical protein | 8.594811 | 7.770823 | 1.1060362 |
| NCED2 | 9-cis-epoxycarotenoid dioxygenase NCED2 | 3.9880927 | 3.6055593 | 1.1060954 |
| ASL9 | LOB domain-containing protein 3 | 4.565558 | 4.1264467 | 1.106414 |
| NF-YA5 | nuclear transcription factor Y subunit A-5 | 3.0310512 | 2.7381244 | 1.1069809 |
| ASY1 | DNA-binding HORMA-like protein | 2.7109363 | 2.4475882 | 1.107595 |
| TRANS11 | phospho-N-acetylmuramoyl-pentapeptide-transferase-like protein | 2.6336493 | 2.3772244 | 1.1078674 |
| CPK29 | calcium-dependent protein kinase 29 | 3.6078553 | 3.2564929 | 1.107896 |
| HSP90.1 | heat shock protein 81-1 | 3.8554122 | 3.479851 | 1.1079245 |
| SQP1 | Squalene monooxygenase 1,1 | 2.6402576 | 2.3828425 | 1.1080285 |
| AT1G70800 | Calcium-dependent lipid-binding domain-containing protein | 3.7590098 | 3.3919537 | 1.1082138 |
| AT1G19970 | ER lumen protein retaining receptor-like protein | 2.6913168 | 2.428256 | 1.1083332 |
| EGY3 | ethylene-dependent gravitropism-deficient and yellow-green-like 3 protein | 2.7199779 | 2.4530742 | 1.1088037 |
| NAC032 | NAC domain containing protein 32 | 9.1350155 | 8.234615 | 1.1093433 |
| AT1G44414 | hypothetical protein | 3.1287968 | 2.820164 | 1.109438 |
| AT5G52050 | mate efflux domain-containing protein | 4.49507 | 4.047531 | 1.1105708 |
| AT5G40810 | ubiquinol-cytochrome c reductase cytochrome c1 subunit | 2.2238765 | 2.0017896 | 1.1109442 |
| AT1G72660 | P-loop containing nucleoside triphosphate hydrolase-like protein | 4.7109413 | 4.239331 | 1.1112465 |
| YSL7 | putative metal-nicotianamine transporter YSL7 | 2.5842853 | 2.3253152 | 1.1113698 |
| AT5G43420 | RING-H2 finger protein ATL16 | 2.7108505 | 2.4379287 | 1.1119481 |
| AT4G12400 | putative stress-inducible protein | 5.180419 | 4.654038 | 1.1131021 |
| UGE2 | UDP-D-glucose/UDP-D-galactose 4-epimerase 2 | 3.1685214 | 2.8459103 | 1.1133596 |
| AT5G49525 | hypothetical protein | 2.654994 | 2.383753 | 1.1137873 |
| AT2G47780 | Rubber elongation factor protein (REF) | 3.8944383 | 3.4942553 | 1.114526 |
| NRT1.7 | nitrate transporter 1.7 | 2.5847192 | 2.3187318 | 1.1147124 |
| MSP2 | microspore-specific promoter 2 | 3.9010472 | 3.4988353 | 1.1149559 |
| AT5G27944 | agamous-like MADS-box protein | 2.4411774 | 2.1894739 | 1.1149608 |
| AT5G64230 | hypothetical protein | 2.502667 | 2.2436116 | 1.1154635 |
| AT1G30974 | Plant thionin family protein | 2.5117724 | 2.2517583 | 1.1154716 |

| | | | | |
|---|---|---|---|---|
| AT1G30810 | transcription factor jumonji and C5HC2 type zinc finger domain-containing protein | 9.998489 | 8.962871 | 1.1155454 |
| AT1G21520 | hypothetical protein | 2.4652028 | 2.2086177 | 1.1161746 |
| SSP4 | SCP1-like small phosphatase 4 | 2.4435904 | 2.1890607 | 1.1162734 |
| AFP1 | Ninja-family protein AFP1 | 14.038125 | 12.572991 | 1.1165303 |
| TAT3 | tyrosine aminotransferase 3 | 2.4418836 | 2.186417 | 1.1168425 |
| AT4G07965 | hypothetical protein | 2.9938674 | 2.6803935 | 1.1169506 |
| PAP18 | purple acid phosphatase 18 | 2.4045472 | 2.1508474 | 1.1179534 |
| IAGLU | indole-3-acetate beta-D-glucosyltransferase | 2.8249722 | 2.5258436 | 1.1184272 |
| AT1G79520 | Cation efflux family protein | 10.307439 | 9.212973 | 1.1187962 |
| MYB3 | transcription factor MYB3 | 2.7521787 | 2.4588003 | 1.1193177 |
| AT1G32560 | Late embryogenesis abundant protein, group 1 protein | 3.4346051 | 3.066657 | 1.1199834 |
| AT5G43150 | hypothetical protein | 2.9843948 | 2.6646748 | 1.1199845 |
| UPS4 | ureide permease 4 | 6.8674507 | 6.131287 | 1.1200668 |
| AT2G27950 | RING/U-box domain-containing protein | 2.4102888 | 2.1511004 | 1.120491 |
| SPDS3 | Spermine synthase | 3.8041651 | 3.3943894 | 1.1207215 |
| AT1G80120 | protein LURP-one-related 5 | 3.4245472 | 3.0549684 | 1.1209763 |
| AT2G02700 | cysteine/histidine-rich C1 domain-containing protein | 60.0329 | 53.547535 | 1.1211141 |
| KIN1 | stress-induced protein KIN1 | 4.1745033 | 3.7232132 | 1.1212097 |
| AT1G64107 | defensin-like protein 269 | 6.753621 | 6.0194573 | 1.121965 |
| UKL5 | putative uracil phosphoribosyltransferase | 3.0219214 | 2.693337 | 1.121999 |
| CYP71B19 | cytochrome P450 71B19 | 2.2874086 | 2.0385659 | 1.1220676 |
| AT3G17720 | Pyridoxal phosphate-dependent transferase-like protein | 2.6100678 | 2.325095 | 1.122564 |
| AT1G73210 | hypothetical protein | 2.4479249 | 2.1790571 | 1.1233872 |
| AT1G76530 | auxin efflux carrier-like protein | 3.3195662 | 2.9534101 | 1.1239773 |
| AT2G20970 | putative lipid binding protein | 16.926514 | 15.056073 | 1.1242318 |
| AT5G56980 | hypothetical protein | 2.546966 | 2.263982 | 1.1249938 |
| ATSDI1 | tetratricopeptide repeat domain-containing protein | 11.46599 | 10.188228 | 1.1254156 |
| AT5G43150 | hypothetical protein | 3.5275614 | 3.1269152 | 1.1281283 |

| | | | | |
|---|---|---|---|---|
| AT4G26701 | DNA binding;DNA topoisomerase type Is | 5.470082 | 4.8486066 | 1.128176 |
| Fes1A | protein Fes1A | 3.7226646 | 3.2995365 | 1.1282387 |
| AT4G39290 | putative F-box/kelch-repeat protein | 5.49779 | 4.8712378 | 1.1286228 |
| AT3G22910 | Ca2+-transporting ATPase | 2.6373618 | 2.3366885 | 1.128675 |
| AT1G66760 | MATE efflux family protein | 3.1780279 | 2.8138998 | 1.1294035 |
| RAP2.1 | ethylene-responsive transcription factor RAP2-1 | 2.7282631 | 2.4153736 | 1.1295408 |
| BGLU24 | beta glucosidase 24 | 3.670711 | 3.2494323 | 1.1296469 |
| PIRL7 | plant intracellular ras group-related LRR 7 | 6.3823175 | 5.649216 | 1.1297705 |
| AT5G64510 | hypothetical protein | 3.6039567 | 3.1899512 | 1.1297842 |
| NF-YB2 | nuclear transcription factor Y subunit B-2 | 5.5685863 | 4.9279227 | 1.1300069 |
| RAP2.10 | ethylene-responsive transcription factor RAP2-10 | 3.6580691 | 3.2363367 | 1.1303116 |
| AT3G03440 | armadillo/beta-catenin-like repeat-containing protein | 2.6294067 | 2.32516 | 1.1308497 |
| AT1G78230 | Outer arm dynein light chain 1 protein | 2.4190967 | 2.1385598 | 1.1311802 |
| ELI3-2 | cinnamyl alcohol dehydrogenase 8 | 4.4111104 | 3.8994024 | 1.1312274 |
| UGT73C7 | UDP-glucosyl transferase 73C7 | 3.97081 | 3.5094244 | 1.1314703 |
| AT3G11420 | hypothetical protein | 2.3142252 | 2.0447903 | 1.1317666 |
| AT3G04010 | O-Glycosyl hydrolases family 17 protein | 2.8786142 | 2.5432227 | 1.1318766 |
| CPuORF17 | hypothetical protein | 2.9853773 | 2.6341498 | 1.1333362 |
| MYB3 | transcription factor MYB3 | 2.7550583 | 2.4307325 | 1.1334271 |
| EP3 | chitinase | 3.6524053 | 3.2219176 | 1.1336123 |
| NRT1.7 | nitrate transporter 1.7 | 3.2098112 | 2.831233 | 1.1337149 |
| AT4G30650 | putative low temperature and salt responsive protein | 2.7693224 | 2.4423711 | 1.1338663 |
| LCR41 | putative defensin-like protein 188 | 3.163851 | 2.7901034 | 1.1339548 |
| KCS2 | 3-ketoacyl-CoA synthase 17 | 4.567396 | 4.0259647 | 1.1344849 |
| KUOX1 | KAR-UP oxidoreductase 1 | 2.5404394 | 2.238407 | 1.1349319 |
| CYP707A2 | abscisic acid 8'-hydroxylase 2 | 3.0646873 | 2.6995077 | 1.1352763 |
| CT-BMY | beta-amylase 3 | 2.515654 | 2.2152412 | 1.1356118 |
| AT5G61390 | exonuclease-like protein | 3.0060136 | 2.6459358 | 1.1360871 |
| BAC2 | Mitochondrial substrate carrier family protein | 2.757571 | 2.4271889 | 1.1361171 |

| | | | | |
|---|---|---|---|---|
| SCL3 | scarecrow-like protein 3 | 3.0417817 | 2.6770654 | 1.1362373 |
| GSL09 | callose synthase | 2.3569984 | 2.0739677 | 1.1364683 |
| RHA3A | RING-H2 finger protein ATL44 | 2.9229753 | 2.570964 | 1.136918 |
| AT5G56050 | hypothetical protein | 2.9884002 | 2.6284368 | 1.1369495 |
| AT5G55970 | RING/U-box domain-containing protein | 2.4136324 | 2.122509 | 1.1371601 |
| AT4G01670 | hypothetical protein | 3.1676323 | 2.785141 | 1.1373329 |
| AT2G16890 | UDP-glycosyltransferase-like protein | 2.9915586 | 2.629995 | 1.1374769 |
| AT5G66460 | mannan endo-1,4-beta-mannosidase 7 | 2.289298 | 2.0119407 | 1.1378555 |
| ROF2 | peptidylprolyl isomerase | 3.9892845 | 3.5055943 | 1.1379766 |
| AT4G14368 | regulator of chromosome condensation repeat-containing protein | 6.8302183 | 6.001751 | 1.1380376 |
| FTSH6 | cell division protease ftsH-6 | 5.19824 | 4.567351 | 1.1381302 |
| TMAC2 | Ninja-family protein AFP4 | 2.388018 | 2.097366 | 1.1385794 |
| AT5G18130 | hypothetical protein | 2.4156818 | 2.119171 | 1.1399183 |
| AT1G10400 | UDP-glycosyltransferase-like protein | 3.3557737 | 2.9413724 | 1.140887 |
| AT5G35688 | hypothetical protein | 8.405684 | 7.367275 | 1.1409489 |
| AT1G01240 | hypothetical protein | 2.3385165 | 2.0496173 | 1.1409527 |
| OBP1 | Dof zinc finger protein DOF3.4 | 2.393413 | 2.0965247 | 1.1416098 |
| GolS3 | galactinol synthase 3 | 3.146311 | 2.7558854 | 1.1416698 |
| ERD4 | Early-responsive to dehydration stress protein (ERD4) | 2.3009179 | 2.015283 | 1.1417344 |
| AT1G78070 | transducin/WD-40 repeat-containing protein | 9.190788 | 8.049211 | 1.1418248 |
| AT3G28007 | nodulin MtN3-like protein | 5.447927 | 4.768594 | 1.1424599 |
| HSP17.4 | heat shock protein 17.4 | 7.0573945 | 6.1768003 | 1.1425648 |
| AT1G05894 | hypothetical protein | 3.388619 | 2.965552 | 1.1426604 |
| HAI2 | protein phosphatase 2C 3 | 34.537853 | 30.197636 | 1.1437271 |
| AT1G21790 | TRAM, LAG1 and CLN8 (TLC) lipid-sensing domain containing protein | 5.582898 | 4.879698 | 1.1441073 |
| AT1G64890 | integral membrane transporter family protein | 2.8663251 | 2.5047789 | 1.1443427 |
| GSTU2 | glutathione S-transferase | 2.4722152 | 2.1597533 | 1.1446748 |
| MTM1 | manganese tracking factor for mitochondrial SOD2 | 3.016761 | 2.634924 | 1.1449139 |
| AT5G41900 | hydrolase, alpha/beta fold family protein | 3.0837631 | 2.6906037 | 1.1461232 |

| | | | | |
|---|---|---|---|---|
| UGT73C1 | cytokinin-O-glucosyltransferase 1 | 9.295618 | 8.108049 | 1.1464679 |
| AT4G28140 | ethylene-responsive transcription factor ERF054 | 17.248396 | 15.043074 | 1.1466006 |
| FIB | putative plastid-lipid-associated protein 1 | 2.8548055 | 2.4893782 | 1.1467947 |
| EXL2 | protein EXORDIUM like 2 | 2.4644418 | 2.1482143 | 1.1472048 |
| SKP2A | F-box protein SKP2A | 3.0304663 | 2.6412368 | 1.1473664 |
| AT3G18950 | transducin/WD40 domain-containing protein | 2.4769785 | 2.1587086 | 1.1474354 |
| AT1G55530 | RING/U-box domain-containing protein | 2.3396761 | 2.038066 | 1.1479884 |
| Fes1A | protein Fes1A | 4.011439 | 3.4894564 | 1.1495885 |
| AT5G20370 | serine-rich protein-like protein | 4.0062213 | 3.4840517 | 1.1498742 |
| AT3G22090 | hypothetical protein | 3.1283014 | 2.720359 | 1.149959 |
| JAZ5 | protein TIFY 11A | 2.682268 | 2.33031 | 1.1510347 |
| AT1G47610 | WD40 domain-containing protein | 4.53364 | 3.9386642 | 1.1510602 |
| AT5G51105 | hypothetical protein | 4.193425 | 3.6429195 | 1.1511166 |
| CPuORF12 | uncharacerized protein | 2.6636357 | 2.3132586 | 1.1514647 |
| AT3G20340 | hypothetical protein | 4.125132 | 3.5815918 | 1.1517594 |
| RAP2.4 | ethylene-responsive transcription factor RAP2-4 | 2.5177004 | 2.1854417 | 1.1520327 |
| AT2G25460 | hypothetical protein | 3.412322 | 2.9572568 | 1.153881 |
| AT3G55090 | ABC transporter G family member 16 | 11.496925 | 9.956196 | 1.1547508 |
| ANK | ankyrin repeat family protein | 2.4409895 | 2.1135623 | 1.1549172 |
| NTM1 | NAC domain-containing protein 68 | 2.9816558 | 2.580267 | 1.155561 |
| AT5G22545 | hypothetical protein | 2.614693 | 2.262353 | 1.1557405 |
| AT4G05140 | Nucleoside transporter family protein | 2.7154884 | 2.349308 | 1.1558673 |
| AT2G03460 | galactose oxidase/kelch-like protein | 4.3624864 | 3.77273 | 1.1563209 |
| FLA3 | fasciclin-like arabinogalactan protein 3 | 2.6533325 | 2.2942808 | 1.1564986 |
| LCR18 | putative defensin-like protein 184 | 2.8078706 | 2.4274669 | 1.1567081 |
| WRKY48 | putative WRKY transcription factor 48 | 2.6301003 | 2.2732198 | 1.1569934 |
| ERD9 | glutathione S-transferase | 3.2355237 | 2.7964063 | 1.1570293 |
| EDL3 | EID1-like F-box protein 3 | 28.205553 | 24.375242 | 1.1571394 |
| PP2-A10 | phloem protein 2-A10 | 2.9851031 | 2.5793035 | 1.1573291 |

| | | | | |
|---|---|---|---|---|
| AT2G23120 | Late embryogenesis abundant protein, group 6 | 3.494842 | 3.0162187 | 1.1586833 |
| SR1 | CBL-interacting serine/threonine-protein kinase 14 | 2.3380404 | 2.017549 | 1.1588517 |
| AT2G33580 | protein kinase family protein | 2.9104564 | 2.5108294 | 1.1591613 |
| AT1G63080 | pentatricopeptide repeat-containing protein | 4.5246625 | 3.903329 | 1.1591805 |
| ALDH2B7 | aldehyde dehydrogenase 2B7 | 2.8040714 | 2.4186425 | 1.1593575 |
| AT5G56160 | sec14p-like phosphatidylinositol transfer-like protein | 2.525246 | 2.1775882 | 1.1596526 |
| AT4G28150 | hypothetical protein | 3.1197178 | 2.6889803 | 1.1601863 |
| AT2G03740 | late embryogenesis abundant domain-containing protein | 2.5336673 | 2.1835651 | 1.1603351 |
| AT5G16380 | hypothetical protein | 2.4409008 | 2.1031597 | 1.1605874 |
| NAP | NAC domain-containing protein 29 | 2.9324236 | 2.522935 | 1.1623064 |
| MYB32 | transcription factor MYB32 | 2.717108 | 2.3373356 | 1.1624808 |
| ICS2 | Isochorismate synthase 2 | 4.163741 | 3.5755196 | 1.1645136 |
| AT2G41870 | remorin-like protein | 2.7869558 | 2.389041 | 1.1665584 |
| ATS1 | caleosin 1 | 7.1638966 | 6.1407948 | 1.1666074 |
| AT1G76590 | PLATZ transcription factor domain-containing protein | 4.13343 | 3.541941 | 1.1669958 |
| ERD9 | glutathione S-transferase | 2.834679 | 2.4289224 | 1.167052 |
| AT5G41320 | hypothetical protein | 2.6564834 | 2.2758992 | 1.1672237 |
| AT4G12000 | SNARE associated Golgi family protein | 6.2684655 | 5.3694634 | 1.1674286 |
| IQD22 | protein IQ-domain 22 | 2.4226606 | 2.0750697 | 1.1675081 |
| AT1G24600 | hypothetical protein | 9.039624 | 7.741478 | 1.1676872 |
| AT3G56790 | RNA splicing factor-related protein | 2.400503 | 2.0552382 | 1.1679925 |
| AT4G16750 | ethylene-responsive transcription factor ERF039 | 2.5704 | 2.2000365 | 1.1683443 |
| AT1G19970 | ER lumen protein retaining receptor-like protein | 2.554572 | 2.1857524 | 1.1687381 |
| AT3G60110 | DNA-binding bromodomain-containing protein | 2.814867 | 2.407082 | 1.1694105 |
| AT4G22620 | SAUR-like auxin-responsive protein | 7.040787 | 6.0132275 | 1.1708833 |
| GolS2 | galactinol synthase 2 | 26.17591 | 22.323755 | 1.1725587 |
| AT3G18490 | aspartyl protease family protein | 3.4763792 | 2.9635837 | 1.1730323 |
| AT3G49130 | SWAP (Suppressor-of-White-APricot)/surp RNA-binding domain-containing protein | 16.063055 | 13.689824 | 1.1733572 |

| | | | | |
|---|---|---|---|---|
| CYP722A1 | cytochrome P450, family 722, subfamily A, polypeptide 1 | 9.0365715 | 7.696871 | 1.1740578 |
| TSPO | tryptophan-rich sensory protein-like protein | 31.560072 | 26.87062 | 1.1745197 |
| AT4G14225 | putative zinc finger A20 and AN1 domain-containing stress-associated protein 8 | 7.8847184 | 6.710889 | 1.1749142 |
| PKT3 | 3-ketoacyl-CoA thiolase 2 | 2.7685745 | 2.355512 | 1.1753601 |
| AT1G20320 | haloacid dehalogenase-like hydrolase | 5.896794 | 5.0164056 | 1.1755018 |
| NAC3 | NAC domain-containing protein 55 | 2.585483 | 2.199372 | 1.1755551 |
| AT3G03170 | hypothetical protein | 5.0182776 | 4.264526 | 1.1767493 |
| AT3G15670 | late embryogenesis abundant domain-containing protein | 91.59765 | 77.83951 | 1.1767501 |
| AT3G11325 | acyltransferase | 12.622323 | 10.726061 | 1.1767901 |
| AT1G53540 | HSP20-like chaperone | 4.3957415 | 3.7338781 | 1.177259 |
| AT5G41590 | hypothetical protein | 9.160335 | 7.7711697 | 1.1787587 |
| AT2G24560 | GDSL esterase/lipase | 5.917697 | 5.0189447 | 1.179072 |
| AT4G23493 | hypothetical protein | 2.8660429 | 2.4289641 | 1.1799445 |
| AT3G63360 | defensin-like protein 11 | 3.311968 | 2.8059034 | 1.1803572 |
| AT1G05894 | hypothetical protein | 4.349819 | 3.6836681 | 1.1808391 |
| AT5G01520 | RING/U-box domain-containing protein | 3.4943593 | 2.9582481 | 1.1812259 |
| AT1G64380 | ethylene-responsive transcription factor ERF061 | 9.978354 | 8.4469595 | 1.1812954 |
| HVA22B | HVA22-like protein b | 4.1888466 | 3.5449507 | 1.1816375 |
| AT4G11570 | haloacid dehalogenase-like hydrolase domain-containing protein | 2.5987797 | 2.1988807 | 1.1818649 |
| MYB112 | myb domain protein 112 | 3.5083878 | 2.9673278 | 1.1823392 |
| RPS2 | disease resistance protein RPS2 | 3.745633 | 3.165398 | 1.1833055 |
| DIN2 | beta-glucosidase 30 | 7.4110255 | 6.262936 | 1.1833149 |
| AT4G16146 | cAMP-regulated phosphoprotein 19-related protein | 2.8592908 | 2.4151773 | 1.1838845 |
| COR413-PM1 | cold regulated 413 plasma membrane 1 | 4.290205 | 3.620758 | 1.1848913 |
| DGK1 | diacylglycerol kinase1 | 3.280598 | 2.768559 | 1.1849477 |
| ERD10 | dehydrin ERD10 | 6.694911 | 5.6494923 | 1.1850464 |
| AT1G55230 | hypothetical protein | 2.7906263 | 2.3547063 | 1.1851271 |
| DREB2A | dehydration-responsive element-binding protein 2A | 11.521972 | 9.719184 | 1.1854876 |
| HSP70T-2 | heat-shock protein 70T-2 | 3.89698 | 3.2847185 | 1.186397 |

| | | | | |
|---|---|---|---|---|
| AT3G51750 | hypothetical protein | 7.004666 | 5.9037676 | 1.1864738 |
| AT5G35660 | glycine-rich protein | 2.874675 | 2.4203956 | 1.1876881 |
| AT1G70130 | concanavalin A-like lectin protein kinase | 4.7565393 | 4.0023513 | 1.1884362 |
| AT3G50390 | transducin/WD40 domain-containing protein | 2.6714907 | 2.2472682 | 1.1887724 |
| BHLH92 | transcription factor bHLH92 | 3.8179176 | 3.2106175 | 1.1891537 |
| AT5G01880 | RING-H2 finger protein ATL74 | 5.2359476 | 4.4027963 | 1.1892322 |
| AT2G38240 | 2-oxoglutarate (2OG) and Fe(II)-dependent oxygenase-like protein | 5.7097354 | 4.799627 | 1.1896207 |
| AT5G15180 | peroxidase 56 | 3.0272627 | 2.542478 | 1.1906741 |
| RKP | ubiquitination-promoting complex protein 1 | 2.8211074 | 2.3687906 | 1.1909485 |
| AT5G40645 | RPM1-interacting protein 4 (RIN4) | 2.9687355 | 2.4923 | 1.191163 |
| AT3G17770 | Dihydroxyacetone kinase | 4.526143 | 3.7982903 | 1.1916264 |
| AT1G80320 | 2-oxoglutarate (2OG) and Fe(II)-dependent oxygenase-like protein | 2.6596525 | 2.2317133 | 1.1917537 |
| AT2G27270 | hypothetical protein | 15.074888 | 12.642724 | 1.1923766 |
| AT3G53820 | C2H2 and C2HC zinc finger-containing protein | 3.1731305 | 2.6602802 | 1.1927806 |
| AT5G07610 | F-box protein | 2.4658613 | 2.0668488 | 1.1930535 |
| AT1G62840 | hypothetical protein | 2.580371 | 2.1609244 | 1.1941051 |
| AT1G74010 | strictosidine synthase | 2.5293808 | 2.118021 | 1.1942189 |
| UGT73B3 | UDP-glucosyl transferase 73B3 | 2.5483115 | 2.1306427 | 1.1960295 |
| DGL | galactolipase DONGLE | 3.177112 | 2.6549973 | 1.1966536 |
| HFR1 | transcription factor HFR1 | 3.320805 | 2.7745426 | 1.1968838 |
| AT3G56260 | hypothetical protein | 6.1358604 | 5.1262045 | 1.1969597 |
| ASY1 | DNA-binding HORMA-like protein | 2.6026511 | 2.1743505 | 1.1969787 |
| AT1G54070 | Dormancy/auxin associated family protein | 2.8301773 | 2.3640933 | 1.1971512 |
| AT1G62975 | transcription factor bHLH125 | 2.5699496 | 2.1465564 | 1.197243 |
| HAI1 | protein phosphatase | 66.85151 | 55.809277 | 1.1978565 |
| AT4G35985 | senescence/dehydration-associated protein | 5.5125723 | 4.601352 | 1.1980331 |
| 42282 | organic cation/carnitine transporter5 | 2.530406 | 2.1105773 | 1.1989164 |
| CID11 | CTC-interacting domain 11 protein | 2.6687126 | 2.2241845 | 1.1998612 |
| AT5G17460 | hypothetical protein | 15.283972 | 12.736639 | 1.2000003 |

| | | | | |
|---|---|---|---|---|
| HSFA6B | heat stress transcription factor A-6b | 101.43073 | 84.51273 | 1.2001828 |
| RAP2.4 | ethylene-responsive transcription factor RAP2-4 | 2.423894 | 2.0164971 | 1.202032 |
| SCL14 | scarecrow-like protein 14 | 2.7286906 | 2.2689471 | 1.2026241 |
| AT5G52390 | PAR1 protein | 4.3659196 | 3.6251192 | 1.204352 |
| TET4 | tetraspanin4 | 3.390391 | 2.8134413 | 1.2050692 |
| HSFC1 | heat stress transcription factor C-1 | 3.6190183 | 3.0010679 | 1.2059102 |
| NAC089 | NAC domain containing protein 89 | 2.56097 | 2.1230688 | 1.2062587 |
| PMZ | zinc finger AN1 domain-containing stress-associated protein 12 | 3.311699 | 2.7452433 | 1.2063408 |
| AT5G25450 | ubiquinol-cytochrome c reductase subunit 7 | 3.0824528 | 2.5542884 | 1.2067754 |
| AT4G15450 | Senescence/dehydration-associated protein-like protein | 2.4628475 | 2.0390902 | 1.207817 |
| AT2G32235 | hypothetical protein | 3.6846604 | 3.049575 | 1.2082537 |
| AT3G17770 | Dihydroxyacetone kinase | 3.0525408 | 2.5252383 | 1.208813 |
| AT3G22275 | hypothetical protein | 6.5429745 | 5.412353 | 1.2088965 |
| AT1G24330 | U-box domain-containing protein 6 | 2.640151 | 2.1839197 | 1.2089049 |
| DEAR3 | ethylene-responsive transcription factor ERF008 | 2.5251458 | 2.0884163 | 1.2091198 |
| AT4G16146 | cAMP-regulated phosphoprotein 19-related protein | 2.7725148 | 2.2923796 | 1.2094483 |
| AT5G10410 | putative clathrin assembly protein | 3.0024548 | 2.4820235 | 1.2096803 |
| AT3G29670 | HXXXD-type acyl-transferase-like protein | 6.8557076 | 5.6668735 | 1.2097867 |
| AFP2 | Ninja-family protein AFP2 | 3.103686 | 2.5640886 | 1.2104442 |
| LCR3 | defensin-like protein 141 | 6.9223948 | 5.7170963 | 1.2108235 |
| AT1G33500 | hypothetical protein | 2.5536125 | 2.1060994 | 1.2124842 |
| AT5G64230 | hypothetical protein | 2.6892807 | 2.215682 | 1.2137485 |
| PP2-B1 | F-box protein PP2-B1 | 2.7210875 | 2.2414415 | 1.21399 |
| ERD7 | senescence/dehydration related protein | 9.682555 | 7.9756317 | 1.2140173 |
| NSP5 | nitrile specifier protein 5 | 2.6620235 | 2.192145 | 1.2143464 |
| AT5G04170 | putative calcium-binding protein CML50 | 2.8870714 | 2.3764293 | 1.2148778 |
| GEX3 | gamete-expressed 3 | 2.8451996 | 2.341688 | 1.2150209 |
| MYB305 | myb domain protein 305 | 13.399659 | 11.024014 | 1.2154973 |
| AT4G06688 | hypothetical protein | 2.8483894 | 2.343368 | 1.2155108 |

| | | | | |
|---|---|---|---|---|
| CDT1 | cadmium tolerance 1 | 2.9760635 | 2.446503 | 1.2164562 |
| LEA18 | late embryogenesis abundant group 1 protein | 21.917835 | 18.008322 | 1.2170948 |
| AT3G48240 | octicosapeptide/Phox/Bem1p domain-containing protein | 6.495038 | 5.3325987 | 1.2179874 |
| AT4G33905 | Peroxisomal membrane 22 kDa (Mpv17/PMP22) family protein | 12.659516 | 10.3938 | 1.2179874 |
| NTMC2T6.2 | calcium-dependent lipid-binding domain-containing protein | 4.165236 | 3.4179142 | 1.2186486 |
| HAI3 | putative protein phosphatase 2C 24 | 509.75458 | 418.1764 | 1.2189941 |
| UBQ3 | polyubiquitin 3 | 2.8028557 | 2.2989187 | 1.2192061 |
| INVH | invertase H | 3.67299 | 3.012369 | 1.2193029 |
| NAC019 | NAC domain-containing protein 19 | 19.593796 | 16.051931 | 1.2206504 |
| AT1G11925 | stigma-specific stig1-like protein | 3.0285997 | 2.481085 | 1.2206755 |
| AT3G17400 | F-box protein | 2.7180097 | 2.2264786 | 1.2207662 |
| AT3G17770 | Dihydroxyacetone kinase | 3.8969157 | 3.1907916 | 1.2213006 |
| CSLD1 | cellulose synthase-like protein D1 | 13.635419 | 11.153275 | 1.2225485 |
| ProT3 | proline transporter 3 | 3.870038 | 3.1643646 | 1.2230064 |
| UGE5 | UDP-D-glucose/UDP-D-galactose 4-epimerase 5 | 3.1042955 | 2.5365236 | 1.2238386 |
| AT5G13880 | hypothetical protein | 3.116898 | 2.5463667 | 1.2240571 |
| AT5G13200 | GEM-like protein 5 | 4.2727766 | 3.490663 | 1.2240586 |
| BIM1 | transcription factor BIM1 | 2.8531575 | 2.3283477 | 1.2254002 |
| PP2-B11 | F-box protein PP2-B11 | 4.6371756 | 3.7836304 | 1.2255889 |
| MYB2 | myb domain protein 2 | 3.3979163 | 2.7714539 | 1.2260411 |
| EDA9 | D-3-phosphoglycerate dehydrogenase | 2.913004 | 2.374837 | 1.2266122 |
| AT1G30280 | chaperone DnaJ-domain-containing protein | 2.7322283 | 2.2271342 | 1.226791 |
| TIP2;2 | putative aquaporin TIP2-2 | 2.9032602 | 2.3664594 | 1.2268372 |
| AT4G38030 | Rhamnogalacturonate lyase family protein | 2.6018617 | 2.120687 | 1.2268956 |
| ASL9 | LOB domain-containing protein 3 | 5.2906547 | 4.308767 | 1.2278814 |
| AT1G32860 | glucan endo-1,3-beta-glucosidase 11 | 4.7005563 | 3.8278756 | 1.2279804 |
| AT2G14290 | F-box protein | 4.78942 | 3.898029 | 1.2286775 |
| AT2G21130 | Peptidyl-prolyl cis-trans isomerase CYP19-2 | 3.3350537 | 2.7135038 | 1.229058 |
| AT3G49200 | O-acyltransferase (WSD1-like) family protein | 2.9677339 | 2.4133499 | 1.2297155 |

| | | | | |
|---|---|---|---|---|
| AT1G07870 | putative serine/threonine-protein kinase RLCKVII | 2.4617667 | 2.0006943 | 1.2304562 |
| RABA6b | RAB GTPase homolog A6B | 3.0625 | 2.4872217 | 1.2312936 |
| AT4G19870 | putative F-box/kelch-repeat protein | 2.8483589 | 2.3116186 | 1.2321924 |
| AT4G14810 | hypothetical protein | 2.9126728 | 2.3599694 | 1.2341994 |
| AT2G22590 | UDP-glycosyltransferase-like protein | 3.1921325 | 2.5859854 | 1.2343968 |
| RD26 | NAC domain-containing protein 72 | 13.311709 | 10.778733 | 1.2349976 |
| LSU3 | response to low sulfur 3 protein | 11.908008 | 9.640079 | 1.2352605 |
| MYB79 | myb domain protein 79 | 40.918556 | 33.108242 | 1.2359024 |
| BOR4 | boron transporter 4 | 6.053803 | 4.8969693 | 1.2362345 |
| GolS4 | galactinol synthase 4 | 9.668084 | 7.8051867 | 1.2386743 |
| AT1G52990 | thioesterase-like protein | 9.10927 | 7.3494678 | 1.2394463 |
| AT4G29930 | transcription factor bHLH27 | 5.7332225 | 4.623246 | 1.240086 |
| AT1G32950 | Subtilase-like protein | 6.0159845 | 4.849748 | 1.2404737 |
| AT5G24870 | RING/U-box domain-containing protein | 3.0807018 | 2.4777145 | 1.2433643 |
| AT1G02030 | C2H2-like zinc finger protein | 3.409178 | 2.7363014 | 1.2459074 |
| AT3G56080 | dehydration-responsive protein-like protein | 2.8349 | 2.2740946 | 1.246606 |
| AT3G19920 | hypothetical protein | 4.509637 | 3.6174362 | 1.246639 |
| AT4G35560 | transducin/WD40 domain-containing protein | 4.597613 | 3.682522 | 1.2484957 |
| AT5G53710 | hypothetical protein | 46.123013 | 36.935677 | 1.2487388 |
| LTI78 | low-temperature-responsive protein 78/desiccation-responsive protein 29A | 36.52818 | 29.240479 | 1.2492334 |
| AT1G22290 | 14-3-3 family protein | 2.9284112 | 2.3399024 | 1.25151 |
| AT4G18280 | glycine-rich cell wall protein-like protein | 4.8712506 | 3.88465 | 1.2539741 |
| NAP | NAC domain-containing protein 29 | 2.780815 | 2.2166123 | 1.2545338 |
| AT1G23450 | pentatricopeptide repeat-containing protein-like protein | 3.007073 | 2.3953218 | 1.255394 |
| AT5G53710 | hypothetical protein | 55.140335 | 43.880043 | 1.2566154 |
| AT4G30350 | heat shock-related protein | 2.5273533 | 2.003382 | 1.2615434 |
| AT5G59490 | haloacid dehalogenase-like hydrolase domain-containing protein | 2.7441437 | 2.1748896 | 1.2617394 |
| AtIPCS1 | inositol phosphorylceramide synthase 1 | 3.853695 | 3.0497243 | 1.2636207 |
| AT2G29500 | HSP20 family protein | 3.5820584 | 2.833905 | 1.2640009 |

| | | | | |
|---|---|---|---|---|
| ABF3 | abscisic acid-insensitive 5-like protein 6 | 8.007179 | 6.333902 | 1.264178 |
| MAPKKK15 | mitogen-activated protein kinase kinase kinase 15 | 3.2776606 | 2.59016 | 1.265428 |
| AT4G22980 | hypothetical protein | 2.914517 | 2.3009543 | 1.2666557 |
| AT1G79520 | Cation efflux family protein | 8.028088 | 6.334635 | 1.2673324 |
| CYP89A5 | cytochrome P450, family 89, subfamily A, polypeptide 5 | 2.8156412 | 2.2200599 | 1.2682728 |
| AT1G75860 | hypothetical protein | 2.793068 | 2.2019744 | 1.2684379 |
| AT1G80450 | VQ motif-containing protein | 2.6401143 | 2.0809405 | 1.268712 |
| NF-YC2 | nuclear transcription factor Y subunit C-2 | 4.605859 | 3.6295838 | 1.2689772 |
| AT1G49450 | WD40 domain-containing protein | 34.732895 | 27.365553 | 1.2692196 |
| LSU1 | response to low sulfur 1 protein | 8.9673 | 7.0602317 | 1.2701143 |
| AT3G21320 | hypothetical protein | 3.2183516 | 2.5332966 | 1.2704203 |
| AT2G04420 | RNase H domain-containing protein | 2.898132 | 2.2807863 | 1.2706723 |
| AT1G51770 | Core-2/I-branching beta-1,6-N-acetylglucosaminyltransferase family protein | 6.1049123 | 4.7996097 | 1.2719603 |
| SIP4 | CBL-interacting serine/threonine-protein kinase 11 | 6.2254243 | 4.887158 | 1.2738333 |
| AT5G04250 | OTU-like cysteine protease family protein | 4.6075644 | 3.616297 | 1.2741112 |
| COR47 | dehydrin COR47 | 2.7671063 | 2.168047 | 1.276313 |
| SKP2B | F-box/LRR-repeat protein 5 | 3.4377475 | 2.6929097 | 1.2765923 |
| DREB2A | dehydration-responsive element-binding protein 2A | 11.60414 | 9.089159 | 1.2767011 |
| ZF2 | zinc-finger protein 2 | 3.83027 | 2.999478 | 1.2769789 |
| AT5G17850 | Sodium/calcium exchanger family protein | 11.147094 | 8.716741 | 1.2788144 |
| HSA32 | Aldolase-type TIM barrel family protein | 3.2881532 | 2.5708604 | 1.2790089 |
| AtS40-3 | protein AtS40-3 | 4.334894 | 3.3881652 | 1.2794224 |
| TIP2;3 | aquaporin TIP2-3 | 21.143965 | 16.524345 | 1.2795645 |
| RAN4 | GTP-binding nuclear protein Ran-4 | 3.1913872 | 2.4940856 | 1.279582 |
| CYP706A7 | cytochrome P450, family 706, subfamily A, polypeptide 7 | 4.6979265 | 3.6708953 | 1.2797768 |
| AT1G76470 | Rossmann-fold NAD(P)-binding domain-containing protein | 2.7645924 | 2.1569483 | 1.2817147 |
| MSL5 | mechanosensitive channel of small conductance-like 5 | 4.1471663 | 3.2338688 | 1.2824165 |
| PDLP1 | cysteine-rich repeat secretory protein 56 | 2.9130993 | 2.271473 | 1.2824715 |
| AT4G34400 | AP2/B3 domain-containing protein | 4.6599145 | 3.6330838 | 1.2826334 |

| | | | | |
|---|---|---|---|---|
| AT2G34990 | RING-H2 finger protein ATL38 | 4.2761064 | 3.3324423 | 1.2831749 |
| AT5G23950 | calcium-dependent lipid-binding domain-containing protein | 4.0756063 | 3.1759741 | 1.2832619 |
| AT1G64380 | ethylene-responsive transcription factor ERF061 | 7.124656 | 5.54967 | 1.2837981 |
| AT5G54420 | hypothetical protein | 2.966461 | 2.3100643 | 1.2841465 |
| FH8 | formin-like protein 8 | 2.8104384 | 2.1874723 | 1.284788 |
| AT5G56160 | sec14p-like phosphatidylinositol transfer-like protein | 2.6635425 | 2.070697 | 1.2863023 |
| AT3G49130 | SWAP (Suppressor-of-White-APricot)/surp RNA-binding domain-containing protein | 16.93192 | 13.1600485 | 1.2866153 |
| AT3G61900 | SAUR-like auxin-responsive protein | 4.422149 | 3.436823 | 1.2866969 |
| AT4G20320 | putative CTP synthase | 3.8301764 | 2.9729528 | 1.2883408 |
| AT2G21320 | B-box zinc finger-like protein | 2.8001277 | 2.1732152 | 1.2884724 |
| AT1G78840 | putative F-box/FBD/LRR-repeat protein | 2.6390636 | 2.0467825 | 1.2893717 |
| AT2G25690 | hypothetical protein | 3.973685 | 3.0769622 | 1.2914313 |
| AT3G53230 | cell division control protein 48-D | 4.5142794 | 3.4954522 | 1.2914722 |
| MAPKKK17 | mitogen-activated protein kinase kinase kinase 17 | 7.6180534 | 5.89711 | 1.2918283 |
| AT5G38240 | Protein kinase family protein | 3.193938 | 2.47218 | 1.2919521 |
| AT2G34030 | calcium-binding EF-hand-containing protein | 2.6133347 | 2.0216582 | 1.2926688 |
| AT4G02950 | Ubiquitin family protein | 20.45135 | 15.81007 | 1.2935647 |
| NAC080 | NAC domain containing protein 80 | 6.628264 | 5.1162148 | 1.2955406 |
| AT4G23450 | RING/U-box domain-containing protein | 3.5169442 | 2.7138069 | 1.2959449 |
| MYB74 | myb domain protein 74 | 9.845511 | 7.5962043 | 1.2961093 |
| UGT75B1 | indole-3-acetate beta-glucosyltransferase 1 | 4.5053596 | 3.4743507 | 1.2967486 |
| UGT84A1 | UDP-glycosyltransferase-like protein | 2.8617415 | 2.2063003 | 1.2970771 |
| MES9 | methyl esterase 9 | 2.626153 | 2.0233161 | 1.297945 |
| AT3G01830 | putative calcium-binding protein CML40 | 7.5291576 | 5.798533 | 1.298459 |
| TIP2;3 | aquaporin TIP2-3 | 14.651527 | 11.274096 | 1.2995745 |
| AT3G43270 | pectinesterase 32 | 3.412841 | 2.6259933 | 1.299638 |
| AT1G67856 | RING/U-box domain-containing protein | 8.415128 | 6.4617357 | 1.3023015 |
| AT1G16850 | hypothetical protein | 7.184984 | 5.515233 | 1.3027526 |

| | | | | |
|---|---|---|---|---|
| AFP3 | Ninja-family protein AFP3 | 7.427146 | 5.7004957 | 1.3028947 |
| CYP81D1 | cytochrome P450 CYP81D11 | 5.500738 | 4.2159734 | 1.3047374 |
| AT2G40435 | hypothetical protein | 2.6752682 | 2.0501606 | 1.3049065 |
| AT3G17130 | plant invertase/pectin methylesterase inhibitor domain-containing protein | 5.7310777 | 4.388123 | 1.306043 |
| AT1G52855 | hypothetical protein | 6.411806 | 4.901335 | 1.3081754 |
| CIPK18 | CBL-interacting serine/threonine-protein kinase 18 | 2.975723 | 2.2736242 | 1.3088017 |
| HSP70 | heat shock protein 70-4 | 3.4307554 | 2.619421 | 1.3097382 |
| AT4G39360 | hypothetical protein | 6.2939343 | 4.800522 | 1.3110938 |
| AT5G35320 | hypothetical protein | 2.7345736 | 2.083586 | 1.3124361 |
| LCR6 | putative defensin-like protein 126 | 4.0966916 | 3.1182525 | 1.313778 |
| AT1G68500 | hypothetical protein | 6.4678617 | 4.9194894 | 1.3147424 |
| RLP33 | receptor like protein 33 | 3.9084527 | 2.9716895 | 1.3152292 |
| AT2G20625 | hypothetical protein | 8.549455 | 6.4991913 | 1.3154644 |
| ABF3 | abscisic acid-insensitive 5-like protein 6 | 7.0449123 | 5.3510985 | 1.3165357 |
| ERF4 | ethylene-responsive transcription factor 4 | 3.1781518 | 2.4132621 | 1.3169526 |
| AT2G21237 | hypothetical protein | 4.2366695 | 3.2151127 | 1.3177359 |
| AT4G39360 | hypothetical protein | 6.0873685 | 4.6191893 | 1.3178436 |
| AT4G35655 | putative nitrate-responsive NOI protein | 7.0149503 | 5.318484 | 1.3189756 |
| AT1G11925 | stigma-specific stig1-like protein | 2.7161043 | 2.0576062 | 1.320031 |
| SNRK2.7 | serine/threonine-protein kinase SRK2F | 2.8907666 | 2.18977 | 1.3201236 |
| TLP3 | Tubby-like F-box protein 3 | 2.8254642 | 2.1402564 | 1.3201523 |
| AT5G44065 | hypothetical protein | 4.289636 | 3.2486322 | 1.3204439 |
| AT3G22920 | peptidylprolyl isomerase | 2.7625847 | 2.0899024 | 1.3218726 |
| CYP71B10 | cytochrome P450 71B10 | 4.0675874 | 3.0747805 | 1.3228871 |
| AT3G03272 | hypothetical protein | 2.7888222 | 2.1079986 | 1.3229716 |
| DEAR3 | ethylene-responsive transcription factor ERF008 | 2.814374 | 2.1205456 | 1.3271934 |
| UGT76E1 | UDP-glucosyl transferase 76E1 | 4.152731 | 3.1269348 | 1.3280517 |
| AT4G25433 | peptidoglycan-binding LysM domain-containing protein | 16.330198 | 12.29054 | 1.3286804 |
| AT5G01200 | duplicated SANT DNA-binding domain-containing protein | 3.160988 | 2.3790386 | 1.328683 |

| | | | | |
|---|---|---|---|---|
| NCED9 | 9-cis-epoxycarotenoid dioxygenase NCED9 | 4.941269 | 3.7163959 | 1.3295863 |
| AT5G05600 | oxidoreductase, 2OG-Fe(II) oxygenase family protein | 2.7006607 | 2.0252042 | 1.3335252 |
| AT3G19240 | Vacuolar import/degradation, Vid27-related protein | 2.7784445 | 2.0789125 | 1.3364894 |
| MYB41 | myb domain protein 41 | 21.358395 | 15.972192 | 1.3372238 |
| AT5G12010 | hypothetical protein | 3.6866632 | 2.75688 | 1.3372592 |
| AT3G12977 | no apical meristem-domain containing transcriptional regulator | 2.9508674 | 2.206649 | 1.3372618 |
| AT1G73510 | hypothetical protein | 12.784129 | 9.535602 | 1.3406734 |
| NF-YC2 | nuclear transcription factor Y subunit C-2 | 4.674577 | 3.4841218 | 1.3416802 |
| SESA1 | seed storage albumin 1 | 3.0451596 | 2.269434 | 1.3418145 |
| NAC046 | NAC domain containing protein 46 | 3.6959527 | 2.7542117 | 1.3419276 |
| AT3G21660 | UBX domain-containing protein | 14.320535 | 10.664126 | 1.34287 |
| MC3 | metacaspase 3 | 3.0099447 | 2.2398033 | 1.3438432 |
| AT3G17770 | Dihydroxyacetone kinase | 5.272687 | 3.9232934 | 1.3439441 |
| SIP4 | CBL-interacting serine/threonine-protein kinase 11 | 5.359159 | 3.9864373 | 1.344348 |
| MAP65-8 | microtubule-associated protein 65-8 | 3.2169602 | 2.3926167 | 1.3445363 |
| AT3G22100 | transcription factor bHLH117 | 4.939729 | 3.67149 | 1.345429 |
| AT1G57590 | Pectin lyase-like protein | 4.048264 | 3.0080192 | 1.3458239 |
| AT3G16800 | putative protein phosphatase 2C 41 | 3.7878346 | 2.8143573 | 1.3458968 |
| AT3G08750 | F-box protein | 25.784407 | 19.140497 | 1.3471128 |
| AFP1 | Ninja-family protein AFP1 | 8.242834 | 6.1154766 | 1.3478645 |
| TET15 | tetraspanin15 | 3.4233532 | 2.5366175 | 1.3495741 |
| CPK15 | calcium-dependent protein kinase 15 | 4.5184917 | 3.3443966 | 1.3510634 |
| AT1G68620 | alpha/beta-hydrolase domain-containing protein | 3.3599057 | 2.4865115 | 1.3512528 |
| AT4G01535 | hypothetical protein | 4.090873 | 3.0259984 | 1.3519086 |
| TIP2;2 | putative aquaporin TIP2-2 | 4.979997 | 3.681734 | 1.3526227 |
| AT1G18830 | transducin/WD-40 repeat-containing protein | 3.545566 | 2.6203291 | 1.3530995 |
| VHA-E2 | V-type proton ATPase subunit E2 | 4.053068 | 2.9901192 | 1.355487 |
| AT1G80160 | lactoylglutathione lyase / glyoxalase I-like protein | 2.8248732 | 2.078727 | 1.3589438 |
| XERO1 | dehydrin xero 1 | 4.9995604 | 3.6665835 | 1.3635473 |

| | | | | |
|---|---|---|---|---|
| AT5G44310 | late embryogenesis abundant domain-containing protein | 2.9486415 | 2.1577263 | 1.3665503 |
| AT2G17660 | RPM1-interacting protein 4-like protein | 5.1581874 | 3.7727687 | 1.3672154 |
| AT5G20690 | leucine-rich repeat protein kinase-like protein | 5.7099957 | 4.1738024 | 1.3680562 |
| AT3G46070 | C2H2-type zinc finger protein | 3.4475856 | 2.51795 | 1.3692033 |
| AT4G02655 | hypothetical protein | 6.925421 | 5.0545588 | 1.3701336 |
| AT3G56260 | hypothetical protein | 7.0118327 | 5.116861 | 1.3703388 |
| GA2OX7 | gibberellin 2-beta-dioxygenase 7 | 6.182223 | 4.5108714 | 1.3705163 |
| LSU1 | response to low sulfur 1 protein | 8.022602 | 5.8500137 | 1.3713819 |
| AT1G32860 | glucan endo-1,3-beta-glucosidase 11 | 3.3205132 | 2.4185865 | 1.3729147 |
| AT3G52060 | Core-2/I-branching beta-1,6-N-acetylglucosaminyltransferase family protein | 42.049057 | 30.565174 | 1.3757178 |
| AT5G16980 | 2-alkenal reductase | 2.9397154 | 2.1338406 | 1.377664 |
| AT1G62975 | transcription factor bHLH125 | 3.0153136 | 2.1886208 | 1.3777231 |
| AT3G57760 | protein kinase family protein | 3.4525642 | 2.5043335 | 1.378636 |
| MAN1 | mannan endo-1,4-beta-mannosidase 1 | 8.835598 | 6.396333 | 1.3813535 |
| AT3G04200 | germin-like protein subfamily 1 member 6 | 6.383215 | 4.6198316 | 1.3816987 |
| AT5G52660 | myb family transcription factor | 2.8170505 | 2.0336463 | 1.3852215 |
| BTI2 | reticulon-like protein B2 | 3.9919698 | 2.881146 | 1.3855493 |
| AT1G30250 | hypothetical protein | 7.0630302 | 5.0945096 | 1.3864005 |
| MAN1 | mannan endo-1,4-beta-mannosidase 1 | 7.1563687 | 5.147941 | 1.3901418 |
| AT5G11410 | protein kinase family protein | 2.9629235 | 2.1307383 | 1.3905619 |
| AT1G19500 | hypothetical protein | 7.39062 | 5.3138976 | 1.3908097 |
| MYB84 | transcription factor RAX3 | 3.9424112 | 2.829031 | 1.3935553 |
| AT2G34360 | MATE efflux family protein | 2.9155068 | 2.0892308 | 1.395493 |
| AT3G56600 | phosphatidylinositol 3- and 4-kinase-like protein | 4.3211346 | 3.0882082 | 1.3992368 |
| AT2G02061 | nucleotide-diphospho-sugar transferase domain-containing protein | 4.9957595 | 3.5681186 | 1.4001102 |
| COR413IM1 | cold regulated 314 inner membrane 1 | 5.4992332 | 3.9221673 | 1.4020904 |
| AT4G30830 | hypothetical protein | 7.106876 | 5.06717 | 1.4025335 |
| AT4G34400 | AP2/B3 domain-containing protein | 6.012957 | 4.2786427 | 1.4053422 |
| ABI5 | protein abscisic acid-insensitive 5 | 4.9324985 | 3.5068018 | 1.4065518 |

| | | | | |
|---|---|---|---|---|
| HOS3-1 | GNS1/SUR4 membrane protein | 3.6023514 | 2.5604708 | 1.4069098 |
| AT5G16960 | 2-alkenal reductase | 39.47992 | 28.044939 | 1.4077377 |
| UGT76E12 | UDP-glucosyl transferase 76E12 | 8.623215 | 6.1214056 | 1.4086984 |
| AT5G24155 | FAD/NAD(P)-binding oxidoreductase family protein | 4.2844133 | 3.0365613 | 1.4109426 |
| AT1G21722 | hypothetical protein | 10.492814 | 7.4293876 | 1.4123391 |
| AT3G03341 | hypothetical protein | 42.769104 | 30.236946 | 1.4144651 |
| AT3G27025 | hypothetical protein | 7.334677 | 5.183223 | 1.4150804 |
| AT4G14730 | Bax inhibitor-1 family protein | 3.7547178 | 2.6523736 | 1.4156067 |
| ABI2 | protein phosphatase 2C 77 | 5.8843026 | 4.1531386 | 1.4168326 |
| AT2G25890 | Oleosin-like protein | 6.4949746 | 4.574924 | 1.4196901 |
| LTI78 | low-temperature-responsive protein 78/desiccation-responsive protein 29A | 40.03432 | 28.182861 | 1.4205201 |
| MYB85 | myb domain protein 85 | 3.320974 | 2.3377407 | 1.4205914 |
| BAM1 | beta-amylase 1 | 7.1663666 | 5.030447 | 1.4245983 |
| ATZIP4 | ZIP4-like protein | 4.3318768 | 3.0354464 | 1.4270971 |
| ASY1 | DNA-binding HORMA-like protein | 13.679837 | 9.559736 | 1.4309847 |
| NIG1 | transcription factor bHLH28 | 10.2736 | 7.176291 | 1.4316031 |
| AT5G35110 | hypothetical protein | 3.0541728 | 2.130992 | 1.4332166 |
| AT4G17550 | MFS transporter, OPA family, solute carrier family 37 | 3.0122356 | 2.1005712 | 1.4340079 |
| AT1G21940 | hypothetical protein | 10.148507 | 7.0765324 | 1.4341074 |
| PDR10 | ABC transporter G family member 38 | 3.6513178 | 2.5436463 | 1.435466 |
| AT1G73510 | hypothetical protein | 17.931911 | 12.485918 | 1.4361707 |
| AT1G07860 | hypothetical protein | 17.35277 | 12.074489 | 1.4371432 |
| AT3G12510 | MADS-box family protein | 4.581384 | 3.1797743 | 1.4407892 |
| AT3G20340 | hypothetical protein | 4.2654023 | 2.9597483 | 1.4411368 |
| DREB2B | dehydration-responsive element-binding protein 2B | 7.7986965 | 5.3975487 | 1.4448589 |
| AT1G10585 | basic helix-loop-helix domain-containing protein | 3.556125 | 2.458095 | 1.4466996 |
| AT2G38250 | DNA-binding protein | 3.4266055 | 2.3565593 | 1.4540715 |
| AT3G12510 | MADS-box family protein | 4.4246726 | 3.0386868 | 1.4561136 |
| JRG21 | jasmonate-regulated protein | 2.937831 | 2.0153737 | 1.4577103 |

| | | | | |
|---|---|---|---|---|
| AT3G14670 | hypothetical protein | 6.250919 | 4.286813 | 1.4581739 |
| MYB121 | myb proto-oncogene protein | 74.905846 | 51.325943 | 1.459415 |
| AT5G62360 | plant invertase/pectin methylesterase inhibitor domain-containing protein | 3.6481657 | 2.4994216 | 1.459604 |
| AT5G16360 | NC domain-containing protein-like protein | 4.5939484 | 3.1471608 | 1.4597121 |
| FLA14 | fasciclin-like arabinogalactan protein 14 | 29.014442 | 19.86224 | 1.4607841 |
| AT3G61900 | SAUR-like auxin-responsive protein | 4.8273764 | 3.2978225 | 1.4638072 |
| AT4G12005 | hypothetical protein | 4.5561633 | 3.1072395 | 1.4663059 |
| CYP94B3 | cytochrome P450, family 94, subfamily B, polypeptide 3 | 7.3077326 | 4.982819 | 1.466586 |
| CBF1 | dehydration-responsive element-binding protein 1B | 8.474016 | 5.7684813 | 1.4690204 |
| AT3G12977 | no apical meristem-domain containing transcriptional regulator | 2.9781435 | 2.0259058 | 1.4700307 |
| SVL3 | protein SEUSS-like 3 | 11.535331 | 7.8440185 | 1.4705895 |
| AT1G70640 | octicosapeptide/Phox/Be.1 domain-containing protein | 33.39988 | 22.6809 | 1.4725993 |
| CYP89A6 | cytochrome P450, family 87, subfamily A, polypeptide 6 | 5.053147 | 3.4141498 | 1.4800601 |
| MYB102 | MYB-like 102 | 10.73974 | 7.2555337 | 1.4802136 |
| AT2G13640 | transcription factor IIS-like protein | 12.099832 | 8.16726 | 1.4815043 |
| UPS4 | ureide permease 4 | 6.290484 | 4.2409086 | 1.4832869 |
| AT2G31930 | hypothetical protein | 3.276021 | 2.2051055 | 1.4856527 |
| AT5G52940 | hypothetical protein | 4.2895584 | 2.8790214 | 1.4899362 |
| EM1 | Em-like protein GEA1 | 4.076982 | 2.7361567 | 1.4900397 |
| AT2G31940 | hypothetical protein | 7.3791823 | 4.951629 | 1.4902534 |
| ERD2 | heat shock protein-70 cognate protein | 3.422777 | 2.295 | 1.491406 |
| RAP2.6 | ethylene-responsive transcription factor RAP2-6 | 4.1466317 | 2.7714522 | 1.4961946 |
| BOR4 | boron transporter 4 | 12.349424 | 8.227757 | 1.5009466 |
| CYP72A9 | cytochrome P450, family 72, subfamily A, polypeptide 9 | 5.6798587 | 3.761273 | 1.5100895 |
| AT3G15200 | pentatricopeptide repeat-containing protein | 3.095525 | 2.0426583 | 1.5154395 |
| AT1G16515 | hypothetical protein | 4.3544683 | 2.872668 | 1.5158272 |
| AT5G45630 | hypothetical protein | 7.229941 | 4.7679477 | 1.5163634 |
| CIPK19 | CBL-interacting serine/threonine-protein kinase 19 | 13.173505 | 8.676855 | 1.518235 |
| AT3G27025 | hypothetical protein | 8.031424 | 5.28716 | 1.5190431 |

| | | | | |
|---|---|---|---|---|
| AT3G17130 | plant invertase/pectin methylesterase inhibitor domain-containing protein | 4.3692636 | 2.8723989 | 1.5211201 |
| AT5G42053 | hypothetical protein | 4.0707297 | 2.675913 | 1.5212487 |
| AT1G76530 | auxin efflux carrier-like protein | 3.7658942 | 2.4731598 | 1.5227056 |
| NAC080 | NAC domain containing protein 80 | 6.540648 | 4.29086 | 1.524321 |
| DGK2 | diacylglycerol kinase 2 | 3.2300851 | 2.1159668 | 1.5265293 |
| AT1G69480 | phosphate transporter PHO1-10 | 7.938423 | 5.1905036 | 1.529413 |
| CDT1 | cadmium tolerance 1 | 3.4548404 | 2.253353 | 1.5331998 |
| AT2G04190 | TRAF-like family protein | 6.241515 | 4.061037 | 1.5369264 |
| SAG29 | senescence-associated protein 29 | 4.159686 | 2.7001376 | 1.540546 |
| AT4G21930 | hypothetical protein | 17.028833 | 11.052691 | 1.5406957 |
| APX1 | L-ascorbate peroxidase | 3.2338538 | 2.0928485 | 1.5451926 |
| AT2G17660 | RPM1-interacting protein 4-like protein | 5.285411 | 3.4177897 | 1.5464412 |
| MYB107 | myb domain protein 107 | 4.867742 | 3.1425166 | 1.5489949 |
| KNAT3 | homeobox protein knotted-1-like 3 | 10.234946 | 6.5986586 | 1.5510646 |
| AT1G57850 | Toll-Interleukin-Resistance domain-containing protein | 8.089746 | 5.210834 | 1.5524858 |
| AT3G27210 | hypothetical protein | 3.2977266 | 2.1232457 | 1.5531536 |
| AT1G55240 | hypothetical protein | 3.3325162 | 2.1442406 | 1.5541708 |
| RAS1 | response to ABA and salt 1 | 6.0293117 | 3.8519757 | 1.5652517 |
| AT1G29680 | hypothetical protein | 28.237911 | 18.010012 | 1.5679008 |
| FBW2 | F-box protein FBW2 | 3.3434107 | 2.129072 | 1.5703605 |
| AT3G62730 | hypothetical protein | 5.2821383 | 3.356498 | 1.5737052 |
| AT5G24080 | protein kinase family protein | 27.669205 | 17.532507 | 1.5781659 |
| MSL5 | mechanosensitive channel of small conductance-like 5 | 6.357161 | 4.023804 | 1.5798882 |
| AT2G34610 | hypothetical protein | 7.321857 | 4.630152 | 1.5813426 |
| HSFA6B | heat stress transcription factor A-6b | 3.1913033 | 2.0109563 | 1.5869582 |
| PDR11 | ABC transporter G family member 39 | 3.2026644 | 2.017856 | 1.5871621 |
| AT4G30830 | hypothetical protein | 6.251365 | 3.9357727 | 1.588345 |
| AT2G33280 | major facilitator protein | 6.872174 | 4.323966 | 1.5893217 |
| AT1G62320 | ERD (early-responsive to dehydration stress) family protein | 3.5254471 | 2.2053263 | 1.5986056 |

| | | | | |
|---|---|---|---|---|
| AT1G05340 | hypothetical protein | 4.993011 | 3.1143792 | 1.6032122 |
| AT1G14048 | GCK domain-containing protein | 4.39448 | 2.7285247 | 1.6105702 |
| P5CS1 | Gamma-glutamyl phosphate reductase | 28.83455 | 17.824625 | 1.6176807 |
| AT1G19490 | bZIP transcription factor-like protein | 4.0224957 | 2.4864063 | 1.6177951 |
| RNS1 | ribonuclease 1 | 3.2753043 | 2.0114527 | 1.6283277 |
| AT1G56660 | hypothetical protein | 3.8419716 | 2.350414 | 1.6345935 |
| AT2G01300 | hypothetical protein | 5.3740425 | 3.268434 | 1.6442255 |
| AT3G23220 | ethylene-responsive transcription factor ERF095 | 4.2497444 | 2.5539305 | 1.6640016 |
| AT1G15010 | hypothetical protein | 3.8125608 | 2.2887805 | 1.6657608 |
| AT5G03210 | hypothetical protein | 21.69327 | 12.97107 | 1.6724349 |
| RRTF1 | ethylene-responsive transcription factor ERF109 | 6.654209 | 3.9599447 | 1.6803793 |
| AT4G21926 | hypothetical protein | 4.164758 | 2.4706843 | 1.68567 |
| AT3G13130 | hypothetical protein | 4.610067 | 2.7076697 | 1.7025958 |
| AT3G50373 | hypothetical protein | 4.856672 | 2.8348396 | 1.7132088 |
| NAMT1 | S-adenosyl-L-methionine-dependent methyltransferase-like protein | 5.6540337 | 3.2949603 | 1.7159641 |
| AT5G39560 | F-box/kelch-repeat protein | 8.194002 | 4.7654805 | 1.7194494 |
| AT1G76965 | hypothetical protein | 11.157238 | 6.4663954 | 1.7254184 |
| AT2G33690 | Late embryogenesis abundant protein, group 6 | 5.1374893 | 2.9766388 | 1.7259364 |
| AT1G77680 | Ribonuclease II/R-like protein | 4.6789036 | 2.6460967 | 1.7682285 |
| CYP72A13 | cytochrome P450, family 72, subfamily A, polypeptide 13 | 7.0993257 | 3.9592865 | 1.7930821 |
| AT3G53040 | putative late embryogenesis abundant protein | 5.4607406 | 3.0329754 | 1.8004568 |
| AT5G43285 | defensin-like protein 217 | 4.8080454 | 2.6312218 | 1.8273052 |
| AT5G62360 | plant invertase/pectin methylesterase inhibitor domain-containing protein | 4.706977 | 2.5645268 | 1.8354174 |
| AT5G62360 | plant invertase/pectin methylesterase inhibitor domain-containing protein | 4.749228 | 2.5422428 | 1.8681254 |
| AT1G80133 | epidermal patterning factor-like protein 8 | 4.2646356 | 2.2712288 | 1.8776776 |
| NAC080 | NAC domain containing protein 80 | 9.045411 | 4.7808905 | 1.8919928 |
| SIP1 | putative galactinol--sucrose galactosyltransferase 5 | 7.5245953 | 3.9636688 | 1.8983915 |
| SPS2F | sucrose phosphate synthase 2F | 6.4162793 | 3.3616407 | 1.908675 |
| AT3G09440 | protein heat shock protein 70-3 | 4.013204 | 2.097905 | 1.9129581 |

| | | | | |
|---|---|---|---|---|
| CBF4 | dehydration-responsive element-binding protein 1D | 31.357681 | 16.305992 | 1.9230771 |
| AT5G62360 | plant invertase/pectin methylesterase inhibitor domain-containing protein | 4.368792 | 2.23942 | 1.9508588 |
| HSP90.1 | heat shock protein 81-1 | 4.804528 | 2.4238973 | 1.9821502 |
| AT4G18650 | transcription factor-related protein | 8.718043 | 4.386015 | 1.9876912 |
| AT1G30250 | hypothetical protein | 4.0762215 | 2.0505886 | 1.9878298 |

**Supplementary Data S6.** Categorization and abundance of degradome reads from the WT+S (wild-type Col-0, NaCl-free), MIR5-S (*35S::miRNVL5*, NaCl-free) and MIR5+S (*35S::miRNVL5*, NaCl-treated) libraries of wild-type and transgenic plants over-expressing miRNVL5. Clean reads are those after removal of low quality reads from raw data.

| | | WT+S | MIR5-S | MIR5+S |
|---|---|---|---|---|
| Raw reads | Total | 30469415 | 24932306 | 27299578 |
| Clean reads | Total | 30200852 | 24739290 | 27064875 |
| | Distinct reads | 6682605 | 7785259 | 6118742 |
| Reads mapping to cDNA | cDNA sense | 17971327 | 16612018 | 17491882 |
| | cDNA antisense | 323197 | 374907 | 224288 |
| | Total number | 18294524 | 16986925 | 17716170 |
| | Total % of clean reads | 60.58% | 68.66% | 65.46% |
| | Distinct reads number | 3734847 | 5266712 | 3641861 |
| | Distinct reads % of clean reads | 55.89% | 67.65% | 59.52% |
| Reads mapping to Genome | Total number | 24750367 | 22232483 | 22361737 |
| | Total % of clean reads | 81.95% | 89.87% | 82.62% |
| | Distinct read number | 4715269 | 6506758 | 4405498 |
| | Distinct reads % of clean reads | 70.56% | 83.58% | 72.00% |

**Supplementary Data S7.** The number of differentially expressed genes in differential expression libraries as assessed by microarray.

| Treatments/Control | Number of up-regulated genes | Number of down-regulated genes |
|---|---|---|
| miR5+S/Col-0+S | 760 | 861 |
| miR5+S/Col-0-S | 3206 | 2938 |
| miR5+S/miR5-S | 2628 | 2442 |
| miR5-S/Col-0-S | 508 | 441 |
| Col-0+S/Col-0-S | 2815 | 2388 |

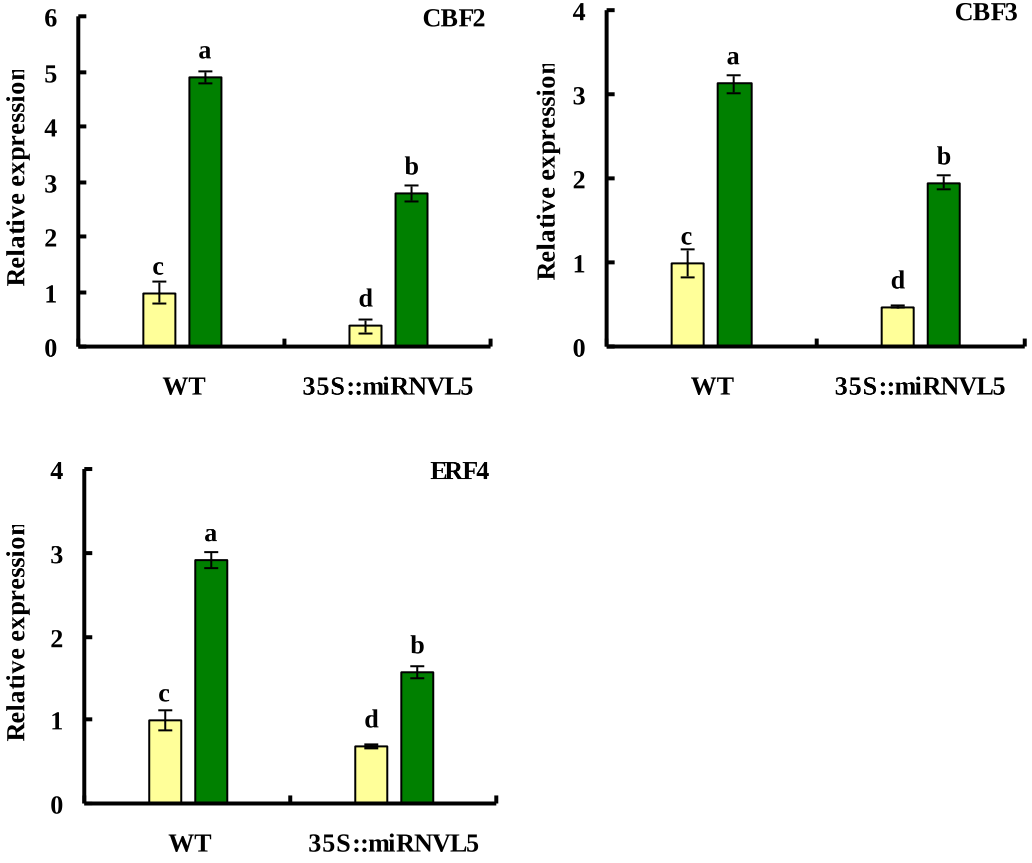


**Supplementary Data S8.** Validation of selected genes from microarray using qRT-PCR. Three week-old wild-type (WT) and *35S::miRNVLU5*

seedlings were treated with 200 mM NaCl exposure for 1 h. Vertical bars represent SD of the mean with three replicates. Data with different letters are significant different ($p<0.05$).

**Supplementary Data S9. Genes co-expressed with At2g44380**

| | | | |
|---|---|---|---|
| 1 | 0.68 | At2g44370* | Cysteine/Histidine-rich C1 domain family protein |
| 2 | 0.61 | At2g27660 | Cysteine/Histidine-rich C1 domain family protein |
| 4 | 0.59 | At1g51850 | Leucine-rich repeat protein kinase family protein |
| 5 | 0.57 | At3g55150 | exocyst subunit exo70 family protein H1 |
| 5 | 0.54 | At2g42350 | RING/U-box superfamily protein |
| 6 | 0.59 | At1g51790 | Leucine-rich repeat protein kinase family protein |
| 8 | 0.58 | At5g64100 | Peroxidase superfamily protein |
| 10 | 0.51 | At5g43520 | Cysteine/Histidine-rich C1 domain family protein |
| 10 | 0.55 | At2g39530 | Uncharacterised protein family (UPF0497) |
| 10 | 0.55 | At3g51330* | Eukaryotic aspartyl protease family protein |
| 11 | 0.53 | At2g44790 | uclacyanin 2 |

| | | | |
|---|---|---|---|
| 11 | 0.51 | At2g39380 | exocyst subunit exo70 family protein H2 |
| 12 | 0.51 | At5g44910 | Toll-Interleukin-Resistance (TIR) domain family protein |
| 12 | 0.53 | At3g46280 | protein kinase-related |
| 12 | 0.48 | At3g46690** | UDP-Glycosyltransferase superfamily protein |
| 12 | 0.5 | At2g36690 | 2-oxoglutarate (2OG) and Fe(II)-dependent oxygenase superfamily protein |
| 12 | 0.51 | At5g48430 | Eukaryotic aspartyl protease family protein |
| 13 | 0.54 | At1g02360 | Chitinase family protein |
| 14 | 0.42 | At4g32870** | Polyketide cyclase/dehydrase and lipid transport superfamily protein |
| 15 | 0.54 | At1g52200 | PLAC8 family protein |
| 16 | 0.5 | At5g24230 | Lipase class 3-related protein |
| 16 | 0.49 | At3g02240 | |
| 18 | 0.49 | At5g53110 | RING/U-box superfamily protein |

| | | | |
|---|---|---|---|
| 18 | 0.48 | At1g51860 | Leucine-rich repeat protein kinase family protein |
| 20 | 0.51 | At5g44380 | FAD-binding Berberine family protein |
| 22 | 0.47 | At4g32650 | potassium channel in Arabidopsis thaliana 3 |
| 22 | 0.51 | At2g44010 | |
| 22 | 0.38 | At4g40070** | RING/U-box superfamily protein |
| 25 | 0.47 | At3g03000** | EF hand calcium-binding protein family |
| 25 | 0.4 | At2g29740 | UDP-glucosyl transferase 71C2 |
| 25 | 0.47 | At1g03850 | Glutaredoxin family protein |
| 25 | 0.47 | At3g45410** | Concanavalin A-like lectin protein kinase family protein |
| 26 | 0.49 | At4g18430 | RAB GTPase homolog A1E |
| 26 | 0.44 | At5g67450 | zinc-finger protein 1 |
| 27 | 0.46 | At4g01720 | WRKY family transcription factor |
| 28 | 0.43 | At1g78000 | sulfate transporter 1;2 |
| 29 | 0.5 | At1g51800 | Leucine-rich repeat protein kinase family protein |

| | | | |
|---|---|---|---|
| 29 | 0.39 | At5g06570 | alpha/beta-Hydrolases superfamily protein |
| 31 | 0.49 | At2g39200 | Seven transmembrane MLO family protein |
| 32 | 0.39 | At1g30110 | nudix hydrolase homolog 25 |
| 33 | 0.43 | At4g16350 | calcineurin B-like protein 6 |
| 33 | 0.47 | At2g42060 | Cysteine/Histidine-rich C1 domain family protein |
| 35 | 0.4 | At1g14780 | MAC/Perforin domain-containing protein |
| 35 | 0.4 | At2g29995 | |
| 36 | 0.46 | At5g64120 | Peroxidase superfamily protein |
| 37 | 0.46 | At2g35000** | RING/U-box superfamily protein |
| 37 | 0.34 | At1g13100** | cytochrome P450, family 71, subfamily B, polypeptide 29 |
| 37 | 0.44 | At1g17860 | Kunitz family trypsin and protease inhibitor protein |
| 38 | 0.39 | At1g22500 | RING/U-box superfamily protein |
| 40 | 0.5 | At5g63600 | flavonol synthase 5 |
| 42 | 0.45 | At1g65610 | Six-hairpin glycosidases superfamily protein |

| | | | |
|---|---|---|---|
| 43 | 0.48 | At1g30700** | FAD-binding Berberine family protein |
| 43 | 0.41 | At4g11530 | cysteine-rich RLK (RECEPTOR-like protein kinase) 34 |
| 43 | 0.49 | At4g20110 | VACUOLAR SORTING RECEPTOR 7 |
| 45 | 0.47 | At1g29280 | WRKY DNA-binding protein 65 |
| 47 | 0.47 | At2g21045 | Rhodanese/Cell cycle control phosphatase superfamily protein |
| 49 | 0.45 | At4g25110 | metacaspase 2 |
| 50 | 0.47 | At3g09490 | Tetratricopeptide repeat (TPR)-like superfamily protein |

*genes that are predicted to interact with At2g44380
**genes that do not participate in the network

## Supplementary Data S10. Primers used for this study by RT-PCR

| Genes | Primers |
|---|---|
| Cotton-18S | F: 5’- TGAGAAACGGCTACCACA -3’<br>R: 5’- ACCCACCCTAAGTCCAAC -3’ |
| (q)RT-Pre-miRNVL5 | F: 5’- GATCTGGGCTTGGGTGCCTATAAAT -3’<br>R: 5’- AATAAAGCAGTACGCAGCACA -3’ |
| 5TF<br>5TR<br>5MF<br>5MR<br>(GhCHR cDNA cloning) | 5’- GAAGATCTATGGAGGAGTCTAACAATTA -3’<br>5’ -GGACTAGTTTACATGCGCCACCACAAAA -3’<br>5’- GCTCTAGAGATCTGGGCTTGGGTGCCT -3’<br>5’- TCCCCCGGGGAGGCAAAGATAATAAAG -3’ |
| RT- GhCHR | F: 5’- TCATCAAAAGCAAGTGGCA -3’<br>R: 5’- CTGAAATAAGGGCAAGAGGTT -3’ |
| GhUB2 | F: 5’- CAAGGAGGGTATCCCCCCA -3’<br>R: 5’- GCAGCCGCAACACCAAGTG -3’ |
| qRT- GhCHR | F: 5’- CTTCTTTCACTCACTGCTCATA -3’<br>R: 5’- CCAACGATTTGTATCTCGACTT -3’ |
| 3'-RACE-Adapt | 5’- GCGAGCACAGAATTAATACGACTCACTATAGGTTTTTTTTTTTTVN -3’ |
| qRT-miRNVL5 | 5’- TTTCTTCACAAATATCACAATA -3’ |
| 3'-Outer | 5’- GCGAGCACAGAATTAATACGACT -3’ |
| Pre-miRNVL5 | F: 5’- GCTCTAGAGATCTGGGCTTGGGTGCCT -3’<br>R: 5’- TCCCCCGGGGAGGCAAAGATAATAAAG -3’ |
| ORF-GhCHR | F: 5’- GCTCTAGAATGGAGGAGTCTAACAATTATGGCC -3’<br>R: 5’- CGAGCTCTTACATGCGCCACCACAAAAAAC -3’ |

| | |
|---|---|
| mGhCHR | F: 5’- AGTCATTACTGTGACATCTGCGAGGAGAGTCGAGATACAAATCGT -3’<br>R: 5’- TCTCGACTCTCCTCGCAGATGTCACAGTAATGACTTTCTAAATAT -3’ |
| pBI121(JD) | 5’- GCGAGAAAGGAAGGGAAGAAA -3’ |
| Actin2 | F: 5’- CATCAGGAAGGACTTGTACGG -3’<br>R: 5’- GATGGACCTGACTCGTCATAC -3’ |
| qRT-RD29A | F: 5’- GTTACTGATCCCACCAAAGAAGA -3’<br>R: 5’- GGAGACTCATCAGTCACTTCCA -3’ |
| qRT-RD22 | F: 5’- AGGGCTGTTTCCACTGAGG -3’<br>R: 5’- CACCACAGATTTATCGTCAGACA -3’ |
| qRT-KIN1 | F: 5’- TGGAGCTGGAGCACAACA -3’<br>R: 5’- GACCCGAATCGCTACTTGTTC -3’ |
| qRT-SOS1 | F: 5’- CACTTCTGGGAAATGGTTGCA -3’<br>R: 5’- TGCCTTCAGCAATGACAACAC -3’ |
| qRT-NHX1 | F: 5’- GTATGGATTTTGGGGTGTTGTCATCA -3’<br>R: 5’- CAGCACAGTGGTGTTTGGTATGCT -3’ |
| qRT-AVP1 | F: 5’- GGGTCAAAACATCTACGGTGTGGAT -3’<br>R: 5’- GGAAACACCACTGCTGCTATTGGAA -3’ |
| qRT-P5CS1 | F: 5’- AGCAGCCTGTAATGCGATGG -3’<br>R: 5’- AAGTGACGCCTTTGGTTTGC -3’ |
| qRT-CBF1 | F: 5’- GCATGTCTCAACTTCGCTGA -3’<br>R: 5’- ATCGTCTCCTCCATGTCCAG -3’ |
| qRT-CBF2 | F: 5’- TGACGTGTCCTTATGGAGCTA -3’<br>R: 5’- CTGCACTCAAAAACATTTGCA -3’ |
| qRT-CBF3 | F: 5’- GATGACGACGTATCGTTATGGA -3’<br>R: 5’- TACACTCGTTTCTCAGTTTTACAAAC -3’ |
| qRT-ERF4 | F: 5’- TCAGCCTCCTCCTGTGACATC -3’<br>R: 5’- ACGACCGATGACGAATCAGAGT -3’ |

| | |
|---|---|
| qRT-AT3G22920 | F: 5'- CGTTGTGATCGGCAAAGTTGTT -3'<br>R: 5'- GGCGTCCTTGTTGTCGTAGTT -3' |
| qRT-AT3G49200 | F: 5'- CATAGAGAACGCAAACGCAGAC -3'<br>R: 5'- CGGCTTGGAGGTGTCTAACG -3' |
